# Supplementary material for: Leveraging publicly available coronavirus data to identify new therapeutic targets for COVID-19
Source: PLoS One. 2021 Sep 29;16(9):e0257965. doi: 10.1371/journal.pone.0257965 (PMC8480897; doi:10.1371/journal.pone.0257965)
Supplement: S1 Table — (PDF) [file pone.0257965.s001.pdf]

Leveraging publicly available coronavirus data to identify new therapeutic targets for COVID-19

Supplemental information

S1 Table. (pages 1–32, genes 1–691)
S2 References. (pages 33–84)

S1 Table. Predicted gene targets of miRNAs that are dysregulated by coronavirus infection and their pro-viral/antiviral functions

Number	Gene	Gene name	Function (curated from peer-reviewed studies)	OMIM	Refs
**(1-36) Predicted (miRDB) target genes of miR-4697-5p that is downregulated by MERS-CoV; target genes are predicted to be upregulated**					
1	WNT1	Wnt1 family member 1	- Negative regulator of virus-induced innate immune responses	* 164820	[*1*]
2	EFNB1	Ephrin B1	- Regulates T cell development and antiviral immune responses.	* 300035	[*2*]
3	PDGFB	Platelet derived growth factor subunit B	- Role in modulating inflammation	* 190040	[*3*]
4	FOXP4	Forkhead box P4	- Transcriptional repressor with role in lung epithelial injury response.	* 608924	[*4*]
5	NRG1	Neuroregulin-1	- Role in mediating cell-cell signaling, elicits immune response	* 142445	[*5*]
6	RHOG	Ras homolog family member G	- Immune response gene activated by viral infection	* 179505	[*6*]
7	TRIM25	Tripartite motif containing protein 25	- Hub gene involved in interferon-inducible innate antiviral defense	* 600453	[*7*]
8	THPO	Thrombopoietin	- Involved in platelet production	* 600044	[*8*]
9	CRTC1	CREB regulated transcription coactivator 1	- Facilitates hepatitis B virus transcription and replication	* 607536	[*9*]
10	CPLX2	Complexin 2	- Expressed in B cells, regulates secretion of immunoglobulins from antibody-secreting cells.	* 605033	[*10*]
11	DLG4 (aka PSD-95)	Discs large MAGUK scaffold protein 4	- Possible link to the cognitive dysfunction documented in Covid-19 patients, PSD-95 is increased in degenerating brains	* 602887	[*11*]
12	MMP15	Matrix metallopeptidase 15	- Inflammation-related gene induced by interleukin-1	* 602261	[*12*]
13	OSMR	Oncostatin M receptor	- Neuroinflammatory gene involved in innate immunity	* 601743	[*13*]
14	CHGA	ChromograninA	- Innate immunity gene	* 118910	[*14*]
15	L1CAM	L1 cell adhesion molecule	- Involved in innate immunity, downregulation alleviates inflammatory neuronal injury	* 308840	[*15*]
16	SLC6A3	Solute carrier family 6 member 3	- Role in innate and adaptive immune response	* 126455	[*16*]
17	HMGA1 [aka HMG-I(Y)]	High mobility group AT-hook 1	- A critical protein cofactor in viral infection and replication, also involved in cardiomyopathy;<br>- Transcriptional activation of the interferon beta gene in response to virus infection requires the assembly of an enhance some, consisting of the transcriptional activators NF-kappa B, IRF1, ATF2/c-Jun, and the architectural protein HMG I(Y)	* 600701	[*17*]<br>[*18*]
18	MSI1	Musashi RNA binding protein 1	- Neural RNA-binding protein that enables replication of another positive-sense RNA virus, Zika, and is thought to be involved in Zika-induced microcephaly.	* 603328	[*19*]

19	SOX3	SRY-box 3	- Master regulator implicated in innate immune function	* 313430	[20]
20	SST	Somatostatin	- Involved in innate immunity pathways	* 182450	[21]
21	SLC12A4	Solute carrier family 12 member 4	- Involved in hepatitis C virus replication	* 604119	[22]
22	NDRG1	N-myc downstream regulated 1	- Facilitates influenza A virus replication by suppressing canonical NF-κB signaling.	* 605262	[23]
23	FOXO6	Forkhead box O6	- Increased with Zika virus infection	* 611457	[24]
24	ATP1A3	ATPase Na+/K+ transporting subunit alpha 3	- Expression linked to neurocognitive disorders	* 182350	[25]
25	Myo18A	Myosin XVIIIA	- Role in activation of innate immune receptors on macrophages.	* 610067	[26]
26	CLEC10A	C-type lectin domain containing 10A	- Innate immune modulator	* 605999	[27]
27	PALM	Paralemmin	- Involved in plasma membrane dynamics and toll-like receptor-mediated inflammatory injury in lungs	* 608134	[28]
28	AP3D1	Adaptor related protein complex 3 subunit delta 1	- Downregulation can reduce viral release from infected cells	* 607246	[29]
29	EIF5A	Eukaryotic translation initiation factor 5A	- Note: the idea about targeting hypusination of EIF5A- which has a role in viral replication- as a broad antiviral strategy	* 600187	[30]
30	TNRC6B	Trinucleotide repeat containing 6B	- Component of host factor complex involved in hepatitis C virus replication	* 610740	[31]
31	PRKCQ	Protein kinase C theta	- Mediator of T cell activation involved in HIV replication	* 600448	[32]
32	KMT2D	Lysine methyltransferase 2D	- Role in immune function	* 602113	[33]
33	FKBP5	FKBP prolyl isomerase 5	- Involved in immunoregulation, innate immune response<br>- Knockdown increases influenza A virus infection	* 602623	[34]<br>[35]
34	IL2RB	Interleukin 2 receptor subunit beta	- Instigator of T cell exhaustion during chronic viral infections	* 146710	[36]
35	C1QTNF3	C1q and TNF related 3	- Associated with increased cardiometabolic risk	* 612045	[37]
36	USP21	Ubiquitin specific peptidase 21	- Negative regulator of antiviral response. i.e. high expression suppresses antiviral interferon signaling.	* 604729	[38]
**(37-58) Predicted (miRDB) gene targets of miR-139-3p that is downregulated by SARS-CoV-1 and SARS- CoV-2; gene targets are predicted to be upregulated**					
37	ARFGEF2	ADP ribosylation factor guanine nucleotide exchange factor 2	- Role in poliovirus replication	* 605371	[39]
38	SH2D3C	SH2 domain containing 3C	- Role in adaptive immunity, regulates B cell development and function	* 604722	[40]
39	VAPB	VAMP associated protein B and C	- Facilitates hepatitis C virus and norovirus replication	* 605704	[41]
40	DCLK1	Doublecortin like kinase 1	- Positively regulates hepatitis C virus replication and virus-induced chronic liver inflammation	* 604742	[42]
41	UVRAG	UV radiation resistance associated gene	- Required for Ebola and other filovirus entry into host cells	* 602493	[43]
42	SIRPA	Signal regulatory protein alpha	- Role in negative regulation of innate immunity, increased expression induced by hepatitis E virus to downregulate expression of type 1 interferons	* 602461	[44]
43	CD177	CD177 molecule	- Role in neutrophil activation and innate and adaptive immune response	* 162860	[45]
44	ARHGDIB	Rho GDP dissociation inhibitor beta	- Negative regulator of HIV replication	* 602843	[46]
45	SDK1	Sidekick cell adhesion molecule 1	- Immunoglobulin family member with role in HIV-associated nephropathy	* 607216	[47]
46	POLDIP3 (aka SKAR)	DNA polymerase delta interacting protein 3	- Role in interferon signaling	* 611520	[48]
47	SPSB1	SplA/ryanodine receptor domain and SOCS box containing 1	- Role in promoting HIV production	* 611657	[49]

48	CD6	CD6 molecule	- Marker of T cells and other immune cells; role in innate and adaptive immunity, overexpression linked to T cell dysfunction	* 186720	[*50*]
49	ATP11A	ATPase phospholipid transporting 11A	- Lipid flippase that is a novel element of the innate immune response that is essential in attenuating the inflammatory response	* 605868	[*51*]
50	SLC2A2 (aka GLUT-2)	Solute carrier family 2 member 2	- Glucose transporter affected in diabetes; possible role in virus-induced diabetes	* 138160	[*52*]
51	ARHGEF28	Rho guanine nucleotide exchange factor 28	- Role in T-cell-dependent humoral immune response	* 612790	[*53*]
52	PACSIN1	Protein kinase C and casein kinase substrate in neurons 1	- Antiviral role in suppressing hepatitis C virus infection<br>- Regulates/activates interferon signaling in dendritic cells	* 606512	[*54*]<br>[*55*]
53	ATL1	Atlastin GTPase 1	- Host protein that is coopted to promote HIV replication and<br>- Zika virus replication	* 606439	[*56*]<br>[*57*]
54	PRDM9	PR/SET domain 9	- Role in antiviral immunity in innate immune response	* 609760	[*58*]
55	TBC1D20	TBC1 domain family member 20	- Role in hepatitis C virus replication and assembly in host cells<br>- Role in HIV replication	* 611663	[*59*]<br>[*60*]
56	RELT	RELT; TNF receptor	- Member of TNFR superfamily that activates NF-κB antiviral immune signaling	* 611211	[*61*]
57	BAZ2B	Bromodomain adjacent to zinc finger domain 2B	- Role in hepatitis delta virus replication	* 605683	[*62*]
58	PLP1	Proteolipid protein 1	- Modulator of innate immune response	* 300401	[*63*]
**(59-302) Predicted (miRDB) gene targets of miR-1290 that is upregulated by SARS-CoV-1 and SARS-CoV-2; gene targets are predicted to be downregulated**					
59	RTKN2	Rhotekin 2	- Role in lymphocyte development	* 618450	[*64*]
60	ACER3	Alkaline ceramidase 3	- Role in mediating innate immune responses; deficiency augments expression of pro-inflammatory cytokines	* 617036	[*65*]
61	GTF2I	General transcription factor IIi	- Role in viral replication	* 601679	[*66*]
62	CFTR	Cystic fibrosis transmembrane conductance regulator	- Critical for lung homeostasis; role in antiviral immunity	* 602421	[*67*]
63	ITGA4	Integrin subunit alpha 4	- Role in immune response	* 192975	[*68*]
64	ATF7IP (aka MCAF1)	Activating transcription factor 7 interacting protein	- Role in activating transcription of Epstein-Barr virus lytic genes	* 613644	[*69*]
65	OGN	Osteoglycin	- Role in cardiac inflammation and injury in viral myocarditis	* 602383	[*70*]
66	AKAP7	A-kinase anchoring protein 7	- May have a proviral effect	* 604693	[*71*]
67	CBLL1	Cbl proto-oncogene like 1	- Role in flavivirus infection	* 606872	[*72*]
68	KDM5A	Lysine demethylase 5A	- Role in innate immune response of natural killer cells	* 180202	[*73*]
69	TNFRSF8	TNF receptor superfamily member 8	- Role in immune regulation	* 153243	[*74*]
70	MSR1	Macrophage scavenger receptor 1	- Role in maintenance of immune homeostasis; promotes pathogenesis of virus-induced fulminant hepatitis; inhibition of this gene would mitigate FH	* 153622	[*75*]
71	NRXN1	Neurexin 1	- Host gene targeted by hepatitis B virus	* 600565	[*76*]
72	KLC1	Kinesin light chain 1	- Motor protein with role in uncoating viral genome and promoting viral infection	* 600025	[*77*]
73	STAG2	Stromal antigen 2	- Loss of this protein leads to interferon signaling and inhibition of viral infection	* 300826	[*78*]
74	CUL4A	Cullin 4A	- Pro-viral function	* 603137	[*79*]
75	PSMC2	Proteasome 26S subunit; ATPase 2	- Role in hepatitis B virus replication	* 154365	[*80*]
76	POU2F2 (aka OCT2)	POU class 2 homeobox 2	- Role in antiviral response of B cells	* 164176	[*81*]
77	PTGER4	Prostaglandin E receptor 4	- Role in inflammation and autoimmunity	* 601586	[*82*]
78	DPT	Dermatopontin	- Novel immune response gene	* 125597	[*83*]
79	ITPR2	Inositol 1,4,5-trisphosphate receptor type 2	- Decreased expression leads to increased innate immunity	* 600144	[*84*]

80	SP1	Sp1 transcription factor	- Transcriptional regulator of myeloid and B cell development and host transcriptional factor that drives viral gene expression	* 189906	[85]
81	STK17A (aka DRAK1)	serine/threonine kinase 17a	- Role in apoptosis of virus-infected cells	* 604726	[86]
82	CREB5	cAMP responsive element binding protein 5	- Negative regulator of hepatitis B virus replication	* 618262	[87]
83	MS4A3	Membrane spanning 4-domains A3	- Macrophage marker with a potential role in respiratory syncytial virus latent infection	* 606498	[88]
84	THSD7A	Thrombospondin type 1 domain containing 7A	- Role in autoimmunity	* 612249	[89]
85	LY75	Lymphocyte antigen 75	- Innate immunity receptor; role in viral infection	* 604524	[90]
86	RGS5	Regulator of G protein signaling 5	- Role in restricting neutrophil chemotaxis and trafficking to inflamed tissues	* 603276	[91]
87	DLG1	Discs large MAGUK scaffold protein 1	- Developmental scaffolding protein with roles in proliferation and function of immune response cells (B, T, dendritic cells), is a negative regulator of HIV-1 infectivity, i.e. decreased expression enhances infectivity	* 601014	[92]
88	USP38	Ubiquitin specific peptidase 38	- Role in negative regulation (inhibition) of antiviral Type 1 interferon signaling	* 618322	[93]
89	RANBP1	RAN binding protein 1	- Role in HIV replication, inhibition leads to decrease in virus	* 601180	[94]
90	ERBB4	Erb-b2 receptor tyrosine kinase 4	- Protective role in liver<br>- Protective role in heart	* 600543	[95]<br>[96]
91	RALGDS	Ral guanine nucleotide dissociation stimulator	- Role in host cell response to viral infection	* 601619	[97]
92	CD164	CD164 molecule	- Interferon-inducible antiviral factor known to suppress HIV production	* 603356	[98]
93	COG5	Component of oligomeric golgi complex 5	- Host response factor that plays a role in HIV replication	* 606821	[99]
94	CD1E	CD1e molecule	- Role in immune response	* 188411	[100]
95	SOCS4	Suppressor of cytokine signaling 4	- Regulator of innate and adaptive immunity that is a critical regulator of antiviral immunity (protects against severe cytokine storm and enhances influenza viral clearance)	* 616337	[101]
96	IL5RA	Interleukin 5 receptor subunit alpha	- Role in innate immune response to influenza virus infection	* 147851	[102]
97	CAMK4	Calcium/calmodulin dependent protein kinase IV	- Role in activating transcription factors in a wide range of immune cells including T cells and antigen-presenting cells	* 114080	[103]
98	TGFB2	Transforming growth factor beta 2	- Multifunctional growth factor and cytokine with role in regulating immune response genes and responses to viral infection	* 190220	[104]
99	VEGFD	Vascular endothelial growth factor D	- Role in formation of lymphatic vessels, immune function, and recently shown to be a potential biomarker of Covid-19 progression- increased levels shown to correlate with severity of Covid-19.	* 300091	[105]
100	KLF9	Kruppel like factor 9	- Role in secondary antibody responses<br>- Dexamethasone-induced KLF9 regulates hepatic glucose metabolism	* 602902	[106]<br>[107]
101	OTOGL	Otogelin like protein	- Interferon-stimulated gene, associated with inner ear function, that may mediate antiviral defenses in bats	* 614925	[108]
102	CALCR	Calcitonin receptor	- Marker of immune function; role in infection and spread of herpes simplex virus type 1	* 114131	[109]
103	MAGOH	Mago homolog, exon junction complex subunit	- Host factor that is recruited to promote viral RNA transport through the cellular mRNA export pathway	* 602603	[110]
104	PHF19	PHD finger protein 19	- Transcriptional regulator with a role in epigenetic reprogramming of T cells	* 609740	[111]
105	RORA	RAR-related orphan receptor A	- Regulator of development, immunity and metabolism	* 600825	[112]

106	TRDMT1 (aka DNMT2)	tRNA aspartic acid methyltransferase 1	- Coopted by HIV (and hepatitis B virus) to increase the stability and survival of the infectious virus genome in host cells.	* 602478	[*113*]
107	ANTXR1	ANTXR cell adhesion molecule 1	- Role in immune response and cellular receptor for Seneca Valley virus, an oncolytic picornavirus	* 606410	[*114*]
108	SMC4	Structural maintenance of chromosomes 4	- Role in organizing and compacting chromosomes and shown to promote inflammatory innate immune response	* 605575	[*115*]
109	XDH	Xanthine dehydrogenase	- Role in innate immune response, including influenza virus infection	* 607633	[*116*]
110	CSTB	Cystatin B	- Role in chemotaxis, antigen presentation and immune and defense response	* 601145	[*117*]
111	ACKR4	Atypical chemokine receptor 4	- Role in controlling chemokine levels and a regulator of early activated B cell differentiation	* 606065	[*118*]
112	APOBEC4	Apolipoprotein B mRNA editing enzyme catalytic polypeptide like 4	- Role in innate and adaptive immunity, has shown both pro-viral i.e. enhances HIV replication and<br>- Antiviral functions	* 609908	[*119*]<br>[*120*]
113	TNIK	TRAF2 and NCK interacting kinase	- Regulator of effector and memory T cell differentiation, role in T cell function during viral infection	* 610005	[*121*]
114	PRKACB	Protein kinase cAMP-activated catalytic subunit beta	- Host response kinase involved in HIV-1 infection and replication	* 176892	[*122*]
115	RAB18	RAB18, member RAS oncogene family	- Host response gene essential for viral infection, replication and assembly	* 602207	[*123*]
116	CAPN2	Calpain 2	- Essential for replication of echovirus 1	* 114230	[*124*]
117	PICALM	Phosphatidylinositol binding clathrin assembly protein	- Host response gene that facilitates viral infection and transport (enterovirus)<br>- Role in herpes simplex virus life cycle	* 603025	[*125*]<br>[*126*]
118	C6orf120	Chromosome 6 open reading frame 120	- Gene linked to type 1 diabetes susceptibility	* 616987	[*127*]
119	PDS5A	PDS5 cohesin associated factor A	- Cell cycle gene with a role in HIV- induced cellular pathogenesis	* 613200	[*128*]
120	UQCRC1	Ubiquinol-cytochrome c reductase core protein 1	- Mitochondrial respiratory chain component (Complex III) linked to mitochondrial dysfunction and cell death induced by Marek's disease virus.	* 191328	[*129*]
121	CLOCK	Clock circadian regulator	- Role in immune function and host-pathogen interactions<br>- Circadian clock dysfunction in SARS-CoV-2 infection	* 601851	[*130*]<br>[*131*]
122	DCX	Doublecortin	- Developmental gene involved in neural progenitor proliferation, differentiation and migration, downregulated by Zika virus and linked to defects in brain development after Zika virus infection	* 300121	[*132*]
123	SNX9	Sorting nexin 9	- Role as a marker sensing immunosuppressed conditions	* 605952	[*133*]
124	JARID2	Jumonji and AT-rich interaction domain containing 2	- Transcriptional repressor with role in embryonic development which increases susceptibility to West Nile virus neuroinvasive disease	* 601594	[*134*]
125	SCP2	Sterol carrier protein 2	- A critical host factor for dengue virus infection	* 184755	[*135*]
126	EBF1	EBF transcription factor 1	- Essential role in B cell development and function<br>- Plays a role in Epstein-Barr virus infection of B cells.	* 164343	[*136*]<br>[*137*]
127	MALT1	MALT1 paracaspase	- Enhances BCL 10-induced activation of NF-κB, role in T cell antigen receptor function, controls rabies virus infection by inducing inflammation and T cell activation	* 604860	[*138*]
128	TBC1D23	TBC1 domain family member 23	- Role in inhibiting innate immunity pathways	* 617687	[*139*]
129	KCNK2 (aka TREK1)	Potassium two pore domain channel subfamily K member 2	- Role in immune cell trafficking into the CNS<br>- Reduced expression in patients with heart failure	* 603219	[*140*]<br>[*141*]
130	RASGRF2	Ras protein specific guanine nucleotide releasing factor 2	- Role in T cell signaling	* 606614	[*142*]
131	P2RY1	Purinergic receptor P2Y1	- Role in platelet function and<br>- Mast cell function in innate immunity	* 601167	[*143*]<br>[*144*]
132	USP49	Ubiquitin specific peptidase 49	- Critical role in terminating innate antiviral responses	* 617470	[*145*]

133	SPATA9	Spermatogenesis associated 9	- Identified as having a role in lung function, possible link to chronic obstructive pulmonary disease	* 608039	[*146*]
134	SH2D1A	SH2 domain containing 1A	- Important role in T cell signaling and antiviral immunity	* 300490	[*147*]
135	ACVR2A	Activin A receptor type 2A	- Role in immune-induced hepatic fibrosis	* 102581	[*148*]
136	COLEC12	Collectin subfamily member 12	- Role in host defense, innate immune modulator	* 607621	[*27*]
137	INHBC	Inhibin subunit beta C	- Possible role in pathogenesis of diabetic nephropathy	* 601233	[*149*]
138	TCN1	Transcobalamin 1	- Role in innate immune responses	* 189905	[*150*]
139	MAP3K2 (aka MEKK2)	Mitogen-activated protein kinase kinase kinase 2	- Role as hub of inflammatory signaling, biomarker of respiratory syncytial virus infection	* 609487	[*151*]
140	ZBTB24	Zinc finger and BTB domain containing 24	- Implicated in autosomal recessive immune disorder	* 614064	[*152*]
141	CNOT7	CCR4-NOT transcription complex subunit 7	- Role in negatively regulating interferon-inducible innate immune function (i.e. knockdown of CNOT7 increases protection against viral infection and reduces viral replication)	* 604913	[*153*]
142	USP15	Ubiquitin specific peptidase 15	- Role in antiviral innate immune signaling	* 604731	[*154*]
143	SEMA6A	Semaphorin 6A	- Role in papillomavirus induced immune response	* 605885	[*155*]
144	EIF5	Eukaryotic translation initiation factor 5	- Role in translation of hepatitis C virus	* 601710	[*156*]
145	ETV3	ETS variant 3	- Transcriptional repressor that contributes to anti-inflammatory effects of IL-10	* 164873	[*157*]
146	FPGT	Fucose-1-phosphate guanylyltransferase	- Role in viral myocarditis	* 603609	[*158*]
147	EPCAM	Epithelial cell adhesion molecule	- Role in immune function and hepatitis B virus infection	* 185535	[*159*]
148	CNTN6	Contactin 6	- Host gene targeted by hepatitis B virus and associated with liver cancers	* 607220	[*76*]
149	RICTOR	RPTOR independent companion of MTOR complex 2	- Role in cell growth and survival, role in dendritic cell function<br>- Co-opted by influenza virus to promote viral replication	* 609022	[*160*]<br>[*161*]
150	REST	RE1 silencing transcription factor	- Role in herpes simplex virus infection and latency	* 600571	[*162*]
151	BECN1	Beclin 1	- Autophagy gene with role in crosstalk between interferon signaling and autophagy	* 604378	[*163*]
152	EID1	EP300 interacting inhibitor of differentiation 1	- Cell cycle gene that is translationally repressed by human cytomegalovirus miRNA	* 605894	[*164*]
153	RNF19A	Ring finger protein 19A, RBR E3 ubiquitin protein ligase	- Immune response gene associated with cross reactions between viral (HBV, HPV) antigens and human proteins that are linked to neuropsychiatric, cardiovascular and metabolic diseases	* 607119	[*165*]
154	DYRK1A	Dual specificity tyrosine phosphorylation regulated kinase 1A	- Role in T cell differentiation and immune homeostasis<br>- Role in human cytomegalovirus infection in placenta<br>- Inhibitors of DYRK have strong antiviral activity	* 600855	[*166*]<br>[*167*]<br>[*168*]
155	CHUK (aka IKK1)	Conserved helix-loop-helix ubiquitous kinase	- Role in interferon-regulated host antiviral signaling	* 600664	[*169*]
156	ATF1	Activating transcription factor 1	- Role in regulating HCMV DNA polymerase promoter during viral infection	* 123803	[*170*]
157	MED23	Mediator complex subunit 23	- Antiviral gene whose altered expression is a risk for cognitive decline and dementia<br>- Antiviral effect against HSV-1 replication	* 605042	[*171*]<br>[*172*]
158	MAZ	MYC associated zinc finger protein	- Transcription factor with role in pro-inflammatory responses, role in influenza virus-induced inflammation in macaque lungs	* 600999	[*173*]
159	GPR34	G protein-coupled receptor 34	- Role in mononuclear cells of immune system<br>- Role in microglial function (microglial cells are the main reservoir of HIV-1 in CNS)	* 300241	[*174*]<br>[*175*]
160	CAMK2D	Calcium/calmodulin dependent protein kinase II delta	- Interferon-induced gene with role in coronavirus infection<br>- Possible role in cardiovascular disease	* 607708	[*176*]<br>[*177*]

161	FOXA2	Forkhead box A2	- Transcriptional regulator of liver-specific genes that also regulates/reduces inflammatory responses in other tissues (e.g. muscle)	* 600288	[178]
162	UHRF2 (aka NIRF)	Ubiquitin like with PHD and ring finger domains 2	- Regulator of cell cycle and genomic stability that inhibits hepatitis B virus replication	* 615211	[179]
163	FYB1 (aka ADAP)	FYN binding protein 1	- Immune adaptor protein with role in T cell signaling, in platelet activation and regulation of interleukin-2 expression, shown to regulate TGFβ1 signaling to protect from influenza virus infection	* 602731	[180]
164	CCNG2	Cyclin G2	- Regulator of cell cycle progression in B cells	* 603203	[181]
165	FBXW7	F-box and WD repeat domain containing 7	- Part of a ubiquitin protein ligase complex with a role in antiviral immunity	* 606278	[182]
166	EME1	Essential meiotic structure-specific endonuclease 1	- Role in DNA damage repair and maintaining genomic stability, role in innate immunity	* 610885	[183]
167	CCNA2	Cyclin A2	- Cell cycle regulator with role in viral pathogenesis	* 123835	[184]
168	ITGAV	Integrin subunit alpha V	- Role in promoting Zika virus infection and a marker of neural stem cells affected by Zika virus	* 193210	[185]
169	MUC21	Mucin 21 cell surface associated	- Important role in inflammatory and innate immune responses to respiratory syncytial virus and human metapneumovirus	* 616991	[186]
170	PLCB1	Phospholipase C beta 1	- Role in lipid metabolism and cancer, prognostic marker of hepatitis B virus-associated hepatocellular carcinoma	* 607120	[187]
171	SFTPB	Surfactant protein B	- Role in alveolar stability, marker of lung inflammation and damage	* 178640	[188]
172	BCL7A	BCL7A, BAF complex component	- Role in Epstein-Barr virus replication and target of an antiviral drug, cordycepin, which increases BCL7A methylation (turns gene expression off)	* 601406	[189]
173	BCCIP	BRCA2 and CDKN1A Interacting protein	- Role in promoting persistent hepatitis C virus infection and replication	* 611883	[190]
174	PRKAA2	Protein kinase AMP-activated catalytic subunit alpha 2	- Role in cardiac metabolism, cardioprotective effect of metformin in individuals who are susceptible to infection-induced myocardial dysfunction is associated, in part, with normalization of PRKAA2 expression which is downregulated by pathogens<br>- Known to be inhibited by hepatitis C virus infection	* 600497	[191]<br>[192]
175	RIPK2	Receptor interacting serine/threonine kinase 2	- Essential role in modulation of innate and adaptive immune responses, role in modulating human cytomegalovirus replication	* 603455	[193]
176	SLTM	SAFB like transcription modulator	- Host protein that is modulated by herpes simplex virus infection		[194]
177	STK3, (aka MST2)	Serine/threonine kinase 3	- Role in maintaining immune system homeostasis, involved in T cell function, role in mediating innate immune response against pathogens and viruses<br>- Host response kinase with role in viral replication	* 605030	[195]<br>[196]
178	GRHL2	Grainyhead like transcription factor 2	- Role in lung epithelial cell morphogenesis and differentiation, role in human papillomavirus-induced oral and pharyngeal cancer	* 608576	[197]
179	IGF2	Insulin like growth factor 2	- Growth factor with implicated role in viral replication	* 147470	[198]
180	PIK3C2A	Phosphatidylinositol-4-phosphate 3-kinase catalytic subunit type 2 alpha	- Host response factor important for production of human cytomegalovirus virions	* 603601	[199]
181	GSTCD	Glutathione S-transferase C-terminal domain containing	- Role in pulmonary function<br>- Genome-wide association studies implicate variants of this gene in chronic obstructive pulmonary disease	* 615912	[200]<br>[201]
182	MVB12B	Multivesicular body subunit 12B	- Role in regulation of HIV budding	* 300188	[202]
183	ZFP36L2	ZFP36 ring finger protein like 2	- Role in translational repression of pre-formed cytokine-encoding mRNAs to prevent chronic activation of memory T cells in the absence of infection	* 612053	[203]
184	SPPL3	Signal peptide peptidase like 3	- Role in natural killer (NK) cell maturation and innate immune response	* 608240	[204]

185	GRIA4	Glutamate ionotropic receptor AMPA type subunit 4	- Glutamate receptor with role in host-directed antiviral response	* 138246	[205]
186	GFRA1	GDNF family receptor alpha 1	- Hepatocellular carcinoma susceptibility gene in patients with hepatitis C virus	* 601496	[206]
187	GPR176	G protein-coupled receptor 176	- Gene expressed in suprachiasmatic nucleus that sets the pace of circadian behavior	* 612183	[207]
188	IGF1	Insulin like growth factor 1	- Growth factor with role in host responses to diverse viruses including influenza A virus (IAV), HIV, RSV, plays important immune function in IAV-mediated acute inflammatory lung injury	* 147440	[208]
189	CYTH2, (aka ARNO)	Cytohesin 2	- Role in interleukin receptor signaling that results in inflammatory cell activation and recruitment and disruption of vascular stability	* 602488	[209]
190	IDE	Insulin degrading enzyme	- Role in terminating insulin activity, functions as a varicella zoster virus receptor<br>- Enhances virus infectivity and stability	* 146680	[210]<br>[211]
191	NDUFA5	NADH:ubiquinone oxidoreductase subunit A5	- Mitochondrial Complex 1 gene, decreased expression linked to insulin resistance and type 2 diabetes	* 601677	[212]
192	KPNA1	Karyopherin subunit alpha 1	- Role in nuclear protein import, role in increasing repression of antiviral interferon-regulated host responses	* 600686	[213]
193	SUMO2	Small ubiquitin-like modifier 2	- Essential negative regulator (inhibitor) of noncanonical type 1 interferon response	* 603042	[214]
194	TRIM58	Tripartite motif containing 58	- Novel negative mediator of innate immune control (negatively regulates TLR2 signaling)	* 616148	[215]
195	TEDDM1	Transmembrane epididymal protein 1	- Marker associated with risk of hepatitis B virus-related hepatocellular carcinoma	* 608336	[216]
196	TRIM33	Tripartite motif containing 33	- Role in innate immunity and antimicrobial infection, transcriptional activator of monocyte/macrophage mediated inflammation	* 605769	[217]
197	FOXC1	Forkhead box C1	- Master regulator of the immune response, regulates toll-like receptors in myocardial ischemia	* 601090	[218]
198	MAPK6, (aka ERK3)	Mitogen-activated protein kinase 6	- Downregulated in hepatitis B virus-induced carcinogenesis<br>- Atypical member of MAP kinase family that is a new regulator of T cell receptor-induced T cell activation	* 602904	[219]<br>[220]
199	CACNB4	Calcium voltage-gated channel auxiliary subunit beta 4	- Role in promoting expression of interferon-related genes in cardiac muscle cells, thereby reducing viral infection	* 601949	[221]
200	SPARCL1	SPARC like 1	- Prosynaptogenic factor with role in Alzheimer's disease	* 606041	[222]
201	TRIM9	Tripartite motif containing 9	- Brain-specific protein involved in synaptic physiology with a role in resolving neuroinflammation and promoting recovery and repair after brain injury, host protein is downregulated by virus infection<br>- Enhances the expression of type 1 interferon as well as interferon-stimulated genes in response to viral infection	* 606555	[223]<br>[224]
202	ABCC4	ATP binding cassette subfamily C member 4	- Multi-drug resistance host protein with role in drug responses of hepatitis B virus patients	* 605250	[225]
203	GALNT13	Polypeptide N-acetylgalactosaminyltransferase 13	- Pulmonary hypertension biomarker	* 608369	[226]
204	SLC11A2	Solute carrier family 11 member 2	- Role in iron transport, role in host metal withholding against invading pathogens	* 600523	[227]
205	SORL1	Sortilin related receptor 1	- Role in endocytosis and protein sorting, gene variant has role in late onset Alzheimer's disease	* 602005	[228]
206	NLGN3	Neuroligin 3	- Involved in synaptic plasticity and implicated in neurodevelopmental disorders such as autism	* 300336	[229]

207	BACE1	Beta-secretase 1	- Role in proteolytic processing of amyloid precursor protein, gene deletion alters immune and inflammatory pathways<br>- Role in HIV-mediated amylodoisis	* 604252	[230]<br>[231]
208	APPBP2	Amyloid beta precursor protein binding protein 2	- Role in processing APP, one of the host genes induced by herpes virus infection in brain cells and implicated in the onset of Alzheimer's disease	* 605324	[232]
209	ERCC8	ERCC excision repair 8, CSA ubiquitin ligase complex subunit	- Role in transcription-coupled nucleotide excision repair, facilitates replication of herpes simplex virus type1	* 609412	[233]
210	GAS2L3	Growth arrest specific 2 like 3	- Cell cycle protein and cytoskeleton orchestrator, essential for genome stability<br>- Essential for brain development<br>- Role in cardiomyocyte cytokinesis during heart development	* 617224	[234]<br>[235]<br>[236]
211	KLK7	Kallikrein related peptidase 7	- Postulated role in virus-mediated central nervous system inflammatory demyelinating disease and in the innate and adaptive immune response	* 604438	[237]
212	ACSL4	Acyl-CoA synthetase long chain family member 4	- Role in fatty acid metabolism in immune cells<br>- Downregulation lead to reduced prostaglandin E2 release from human arterial smooth muscle cells	* 300157	[238]<br>[239]
213	STXBP5, (aka TOMOSYN)	Syntaxin binding protein 5	- Role in exocytosis and neurotransmitter release, controls the dendritic stability of neurons and surface expression of AMPA receptors	* 604586	[240]
214	CDH6	Cadherin 6	- Host protein activated by Epstein-Barr virus to drive metastasis of nasopharyngeal carcinoma	* 603007	[241]
215	IGFBP3	Insulin like growth factor binding protein 3	- Role in protecting against corneal inflammation and scarring caused by herpes simplex virus corneal infection	* 146732	[242]
216	HLTF	Helicase like transcription factor	- Role in DNA repair in host cells, restricts HIV-1 replication in dividing T cells, HIV-1 proteins counteract this restriction	* 603257	[243]
217	INKA2	Inka box actin regulator 2	- Involved in cell migration and neuronal polarity, downregulated in fibroblasts of idiopathic pulmonary fibrosis patients		[244]
218	SLC25A32	Solute carrier family 25 member 32	- Role in transporting folate across the inner mitochondrial membrane, dysregulation associated with multiple nervous system diseases	* 610815	[245]
219	PARVA	Parvin alpha	- Actin-binding protein, required for normal development of embryonic cardiovascular system, role in cardiac myocyte hypertrophy	* 608120	[246]
220	LGR4	Leucine rich repeat containing G protein-coupled receptor 4	- Host protein with role in facilitating vesicular stomatitis virus infection<br>- Governs pro-inflammatory program in macrophages to antagonize post-infarction cardiac repair<br>- Recently identified as part of a lung signature in SARS-CoV and SARS-CoV-2 infected ferrets and a potential treatment target	* 606666	[247]<br>[248]<br>[249]
221	ZNF502	Zinc finger protein 502	- Host factor important for respiratory syncytial virus replication	* 606234	[250]
222	MMP16	Matrix metallopeptidase 16	- Variant of this gene is implicated in predisposition to rhinovirus lower respiratory tract infections in premature infants<br>- Downregulation is part of host defense response in asymptomatic dengue virus infected individuals	* 602262	[251]<br>[252]
223	SHANK1	SH3 and multiple ankyrin repeat domains 1	- Required for development and function of neuronal synapses, mutations or dysregulated expression associated with neuropsychiatric disorders	* 604999	[253]
224	WBP1L	WW domain binding protein 1	- Like candidate gene for schizophrenia	* 611129	[254]
225	CUL2	Cullin 2	- Core component of E3 ubiquitin-protein ligase complex, also a marker of HIV infection	* 603135	[255]

226	TNFRSF11A, (aka RANK)	TNF receptor superfamily member 11a	- Involved in regulation of interactions between T cells and dendritic cells, role in cardiovascular and bone disease associated with HIV infection	* 603499	[*256*]
227	CRNDE	Colorectal neoplasia differentially expressed	- Role as a non-coding RNA involved in regulating genes involved in metabolism, overexpression attenuates cardiac fibrosis and enhances cardiac function	* 615624	[*257*]
228	MAP3K7, (aka TAK1)	Mitogen-activated protein kinase kinase kinase 7	- Role in TGF-β and BMP signaling, essential role in innate immune responses<br>- Regulates inflammatory cytokine production in natural killer cells<br>- Role in TRIM5 signaling to block infection by HIV and other retroviruses	* 605101	[*258*]<br>[*259*]<br>[*260*]
229	HHEX	Hematopoietically expressed homeobox	- Transcription factor with role in developmental processes, overexpression induced by human cytomegalovirus infection	* 604420	[*261*]
230	KLHL12	Kelch like family member 12	- Role as negative regulator of Wnt signaling, role in downregulation of enterovirus IRES-mediated translation in infected cells	* 614522	[*262*]
231	PTK2, (aka FAK)	Protein tyrosine kinase 2	- Essential role in cell migration and adhesion, and in embryonic development, role in host defense to infection,<br>- Dysregulation of this gene together with Sox11 plays a role in ventilator-induced lung injury<br>- Regulates/promotes the activity of multiple influenza A virus subtypes	* 600758	[*263*]<br>[*264*]<br>[*265*]
232	LDHA	Lactate dehydrogenase A	- One of five isoforms of a key enzyme involved in anaerobic glycolysis, suppression of LDHA can enhance virus replication	* 150000	[*266*]
233	NMU	Neuromedin U	- Neuropeptide involved in pain, stress, immune-mediated inflammatory diseases and feeding regulation, promotes type 2 innate lymphoid cell (ILC2)-driven allergic lung inflammation<br>- Mediates the neuronal regulation of type 2 innate lymphoid cells that regulate inflammation, tissue repair and metabolic homeostasis	* 605103	[*267*]<br>[*268*]
234	MSTN	Myostatin	- Role as a negative regulator of muscle development, expression found increased in HIV-positive men	* 601788	[*269*]
235	FIGN	Fidgetin, microtubule severing factor	- Gene variant has a role in protection against congenital heart disease	* 605295	[*270*]
236	HSPA5, (aka GPR78)	Heat shock protein family A (Hsp70) member 5	- Endoplasmic reticulum chaperone that plays a key role in protein folding, role in innate immune signaling,<br>- Host receptor that facilitates viral entry of Zika virus	* 138120	[*271*]<br>[*272*]
237	PHLDA1	pleckstrin homology like domain family A member 1	- Role in regulation of apoptosis, role in oxidative stress-induced cardiomyocyte injury and myocardial ischemia reperfusion injury<br>- Promotes microglia-mediated neuroinflammation	* 605335	[*273*]<br>[*274*]
238	FBXW11	F-box and WD repeat domain containing 11	- Part of ubiquitin-protein ligase complex which mediates ubiquitination and subsequent proteosomal degradation of target proteins, host protein that is co-opted by Rift Valley fever virus to degrade the host antiviral protein kinase PKR which is involved in type 1 interferon responses	* 605651	[*275*]
239	FADS1	fatty acid desaturase 1	- Role in lipid metabolism, key enzyme in endogenous fatty acid synthesis, gene variants are associated with 15 cardiovascular diseases	* 606148	[*276*]
240	SRGAP2	SLIT-ROBO Rho GTPase activating protein 2	- Gene found only in humans, essential for development of cerebral cortex, changes in expression linked to neurodevelopment disorders and inflammation	* 606524	[*277*]

241	PRLR	Prolactin receptor	- Receptor for the anterior pituitary hormone prolactin, plays important roles in human immune responses by influencing the expression of costimulatory molecules and cytokines in T lymphocytes	* 176761	[*278*]
242	SFXN1	Sideroflexin 1	- Host gene linked to pathogenesis of respiratory syncytial virus and metapneumovirus	* 615569	[*279*]
243	PAK2	p21 (RAC1) activated kinase 2	- Role in a variety of signaling pathways, essential for function of FoxP3-positive regulatory T cells,<br>- Mediates cardioprotective endoplasmic reticulum stress response	* 605022.	[*280*]<br>[*281*]
244	USP13	Ubiquitin specific peptidase 13	- Deubiquitinase involved in autophagy and ER associated degradation<br>- Identified as a protein that interacts with SARS-CoV-2 in immune cells	* 603591	[*282*]<br>[*283*]
245	POU3F2	POU class 3 homeobox 2	- Transcription factor that plays a key role in neuronal differentiation, also associated with left ventricular remodeling in hypertension<br>- Shown to interact with herpes simplex virus alpha gene promoters	* 600494	[*284*]<br>[*285*]
246	SPON1	Spondin 1	- Role in cell adhesion, immune response and inflammation, candidate gene for hypertension, upregulated in kidney of humans with diabetic neuropathy and increased in kidney of HIV-1 transgenic mice	* 604989	[*286*]
247	BBS9	Bardet-Biedl syndrome 9	- Potential role in adenovirus-infected B cells	# 615986	[*287*]
248	AP5B1	Adaptor related protein complex 5 subunit beta 1	- Role in trafficking of HIV-2 in host cells	* 614367	[*288*]
249	CLIC4	Chloride intracellular channel 4	- Role in membrane trafficking, apoptosis, angiogenesis and cell differentiation, shown to have role in host innate responses to bacterial lipopolysaccharide in macrophages<br>- Required for efficient genome replication of chikungunya virus<br>- Implicated as a key host cell factor that contributes to Merkel cell polyomavirus-mediated cellular transformation	* 606536	[*289*]<br>[*290*]<br>[*291*]
250	OTUD4	OTU deubiquitinase 4	- A marker for multiple cancers, induction of expression by viral infection promotes innate antiviral response, i.e. knockdown leads to decreased levels of type 1 interferons and proinflammatory cytokines and potentiates viral replication	* 615712	[*292*]
251	NLRP3	NLR family pyrin domain containing 3	- Crucial role in innate immunity and inflammation as the sensor component of the NLRP3 inflammasome, involved in innate immune sensing of influenza A virus and other viruses<br>- NLRP3 inflammasome is dysregulated in severe Covid-19 patients<br>- Drugs targeting NLRP3 inflammasome suggested as potential treatments for Covid-19 patients	* 606416	[*293*]<br>[*294*]<br>[*295*]
252	TNIP1, (aka ABIN-1)	TNFAIP3 interacting protein 1	- Role as a suppressor of innate immune response<br>- Increased expression contributes to dampening of M1 macrophage polarization during hepatitis C virus infection<br>- Role in inhibition of antiviral signaling	* 607714	[*296*]<br>[*297*]<br>[*298*]
253	TPH2	Tryptophan hydroxylase 2	- Catalyzes the first and rate limiting step in serotonin biosynthesis, gene variant implicated in interferon-induced depression in chronic hepatitis C virus patients	* 607478	[*299*]
254	DGAT1	Diacylglycerol O-acyltransferase 1	- Critical role in biosynthesis of endogenous triglycerides and essential host factor for hepatitis C virus (HCV) production, DGAT inhibitor suppresses HCV genome replication and particle production	* 604900	[*300*]

255	PXMP4	Peroxisomal membrane protein 4	- Ubiquitously expressed in lung, plays role in modulating expression of natural killer T cells	* 616397	[301]
256	MTMR4	Myotubularin related protein 4	- Role in dephosphorylating proteins, negatively regulates innate immune responses, i.e. in macrophage cells with MTMR4 gene knocked out, there is increased type 1 interferon production	* 603559	[302]
257	ERCC4	ERCC excision repair 4, endonuclease catalytic subunit	- Role in DNA repair, possible role in hepatitis B virus-related hepatocellular carcinoma	* 133520	[303]
258	KIAA1958	KIAA1958	- Possible association with childhood asthma and risk variant for chronic obstructive pulmonary disease (COPD) based on genome-wide association studies	* 617390	[304]
259	DCP2	Decapping mRNA 2	- Decapping enzyme required for degradation of mRNAs, important modulator (negative regulator) of genes involved in type 1 interferon response, particularly interferon regulatory factor-7, a key transcription factor, a negative feedback mechanism to restore homeostasis following viral infection	* 609844	[305]
			- Pro-viral role as a host gene co-opted by HIV-1 and Feline immunodeficiency virus for viral capsid assembly in host cells		[306]
260	MAP3K1, (aka MEKK1)	Mitogen-activated protein kinase kinase kinase 1	- Activator of several signal transduction pathways including ERK and JNK pathways, and numerous signaling pathways involved in inflammation and apoptosis, gene variant associated with a greater inflammatory response, i.e. decreased ventilator-free days in patients with acute respiratory distress syndrome	# 613762	[307]
			- Role in antiviral pathway, required for optimal induction of type 1 interferons		[308]
261	CXCR3	C-X-C motif chemokine receptor 3	- Receptor for three interferon-inducible chemokines, important for innate immunity	* 300574	[309]
			- Upregulated by SARS-CoV infection in lungs of infected mice		[310]
			- Because of its expression on the X chromosome, may play a role in the differential response of women and men to SARS-CoV-2 infection		[311]
262	TAB3	TGF-beta activated kinase 1 (MAP3K7) binding protein 3	- Role in NF-kappaB signal transduction pathway, host gene required for virus replication	* 300480	[312]
263	CHMP4B	Charged multivesicular body protein 4B	- Role in sorting of endocytosed cell-surface receptors into multivesicular endosomes, host protein co-opted by HIV to mediate virus release from infected cells	* 610897	[313]
264	FAM120B	Family with sequence similarity 120B	- Role in adipogenesis, identified via genome-wide meta-analysis as part of a gene network predisposing to type 1 diabetes	* 612266	[127]
265	KPNA6	Karyopherin subunit alpha 6	- Role in nuclear protein import, recruited by influenza virus to enter host cell nucleus and promote viral replication	* 610563	[314]
			- Interaction with Ebola virus blocks type 1 interferon signaling		[315]
			- Also required for replication of Zika and porcine reproductive and respiratory syndrome virus		[316]
266	SEC62	SEC62 homolog preprotein translocation factor	- Role in post-translational transport of precursor polypeptides across the endoplasmic reticulum, role in innate antiviral immune responses	* 602173	[317]
267	MT1H	Metallothionein 1H	- Metal-binding protein induced by oxidative stress, role in hepatitis C virus-associated systemic vasculitis neuropathy	* 156354	[318]
268	EN2	Engrailed homeobox 2	- Role in controlling development, gene knockout is a mouse model of autism spectrum disorder	* 131310	[319]
			- Role in inducing antiviral activity in the stroma		[320]

269	HOXC8	Homeobox C8	- Transcription factor that plays an important role in morphogenesis in all organisms, repressed by Epstein-Barr virus latency protein	* 142970	[321]
270	NRAS	NRAS proto-oncogene GTPase	- Role as an oncogene, also has role in antiviral immune response and T cell function and development	* 164790	[322]
271	CDC27	Cell division cycle 27	- Role in cell cycle regulation, downregulated by chikungunya virus infection	* 116946	[323]
272	PDGFD	Platelet derived growth factor D	- Role as a growth factor that plays an essential role in regulation of development, cell proliferation, cell migration, cell survival and chemotaxis, dysregulation as part of a diagnostic signature for myocardial infarction<br>- Induced by cytomegalovirus	* 609673	[324]<br>[325]
273	HELZ	Helicase with zinc finger	- Possible role in cardiomyocyte death under hypoxic conditions	* 606699	[326]
274	TMEM106B	Transmembrane protein 106B	- Required for dendrite morphogenesis and maintenance, loss of expression leads to severe lysosomal abnormalities and neurodegeneration in mice	* 613413	[327]
275	CASP1, (aka IL1BC)	Caspase 1	- Central role in execution phase of cell apoptosis, role in infection by diverse viruses such as Zika virus<br>- Role in influenza A virus infection<br>- Role in Mayaro virus infection	* 147678	[328]<br>[329]<br>[330]
276	CDK6	Cyclin dependent kinase 6	- Key role in cell cycle regulation, shown to have antiviral effects against SARS-CoV	* 603368	[331]
277	HGF	Hepatocyte growth factor	- Regulates cell growth, motility and morphogenesis in a broad spectrum of tissues and cell types, recently identified as a marker of Covid-19 disease severity	* 142409	[332]
278	STAM2	Signal transducing adaptor molecule 2	- Role in intracellular signal transduction mediated by cytokines and growth factors, highly expressed in neurons and may be involved in T cell development	* 606244	[333]
279	BTD	Biotinidase	- Role in recycling protein bound biotin, deficiency leads to neurological and cellular immunological abnormalities<br>- Deficiency also enhances the innate inflammatory responses of dendritic cells	* 609019	[334]<br>[335]
280	MYOCD	Myocardin	- Transcriptional co-activator of serum response factor (SRF), modulates expression of cardiac and smooth muscle-specific SRF-target genes, deregulation implicated in coronary artery disease<br>- Downregulated expression associated with intracranial aneurysm	* 606127	[336]<br>[337]
281	KIF13A	Kinesin family member 13A	- Microtubule-dependent motor protein with role in intracellular transport, involved in intracellular trafficking of influenza A virus ribonucleoproteins<br>- Role in intracellular trafficking of Lassa virus matrix protein	* 605433	[338]<br>[339]
282	RALB	RAS like proto-oncogene B	- Multifunctional GTPase involved in a variety of cellular processes, role in immune response of natural killer cells to viral infection	* 179551	[340]
283	C9	Complement C9	- Constituent of membrane attack complex that plays a key role in innate and adaptive immune response, involved in coronavirus induced demyelination<br>- Downregulated expression of C9 in chronic hepatitis C virus liver may be mechanism of immune evasion by HCV	* 120940	[341]<br>[342]
284	CASP14	Caspase 14	- Non-apoptotic caspase involved in epidermal differentiation, increased expression triggered by chronic hepatitis C virus infection	* 605848	[343]
285	TSPYL4	TSPY like 4	- Single nucleotide polymorphisms (SNPs) in this gene are associated with pulmonary function and		[344]

			susceptibility to chronic obstructive pulmonary disease		
286	POU2F1, (aka OCT1)	POU class 2 homeobox 1	- Transcription factor, has role in T cell differentiation<br>- Important role in facilitating HIV infection<br>- Role in herpes simplex virus infection	* 164175	[345]<br>[346]<br>[347]
287	MARCHF3	Membrane associated ring-CH-type finger 3	- Critical negative regulator (attenuates) of IL-1β-triggered inflammation<br>- Loci associated with type 2 diabetes mellitus	* 613333	[348]
288	KSR2	Kinase suppressor of ras 2	- Highly expressed in brain, regulates energy balance and glucose homeostasis	* 610737	[349]
289	COL21A1	Collagen type XXI alpha 1 chain	- Recently identified as having a role in dilated cardiomyopathy	* 610002	[350]
290	TET2	Tet methylcytosine dioxygenase 2	- Involved in myelopoiesis and immune function, degraded by HIV-1 Vpr to enhance HIV-1 replication in macrophages	* 612839	[351]
291	CLVS1	Cavesin 1	- Required for normal morphology of late endosomes and/or lysosomes in neurons, genetic association with schizophrenia and bipolar disorder	* 611292	[352]
292	CAPZA1	Capping actin protein of muscle Z-line subunit alpha 1	- Cytoskeletal host protein that binds to Chikungunya virus	* 601580	[353]
293	IRF1	Interferon regulatory factor 1	- Transcriptional regulator of innate and acquired immune responses, including interferon and interferon-inducible genes, important in antiviral defense against a broad spectrum of viral pathogens, i.e. influenza<br>- Hyperactivation contributes to more robust antiviral response	* 147575	[354]<br>[355]
294	CUX1	Cut like homeobox 1	- Transcription factor involved in neuronal differentiation, dendrite development and branching, role in repressing human cytomegalovirus gene expression essential for viral replication	* 116896	[356]
295	ITPA	Inosine triphosphatase	- Pyrophosphatase which is shown to have a role in the antiviral actions of ribavirin, an antiviral drug against respiratory syncytial virus, hepatitis C virus and other viruses	* 147520	[357]
296	DNAJC30	DNAJ heat shock protein family (Hsp40) member C30	- Mitochondrial protein enriched in neurons that has a role in regulating mitochondrial respiration	* 618202	[358]
297	PTPRA	Protein tyrosine phosphatase, receptor type A	- Role in immune cell function	* 176884	[359]
298	NLK	Nemo like kinase	- Role in regulating a number of transcription factors with key roles in cell fate determination, role in antiviral innate immune response, depletion of NLK promotes antiviral cytokine production and decreases viral replication	* 609476	[360]
299	STC1	Stanniocalcin 1	- Role in preventing hypercalcemia by stimulating renal phosphate reabsorption, multifunctional glycoprotein with antioxidant and anti-inflammatory properties, shown to attenuate ischemic cardiac injury by limiting the effects of inflammatory stimuli on monocytes and macrophages	* 601185	[361]
300	SDC2	Syndecan 2	- Role in cell proliferation and migration and cell-matrix interactions, role as attachment receptor for hepatitis C virus	* 142460	[362]
301	SOD2	Superoxide dismutase 2	- Role in destroying superoxide anion radicals which are toxic to cells, role in cardioprotective effects of several drugs<br>- Facilitates the antiviral innate immune response	* 147460	[363]<br>[364]
302	VPS36	Vacuolar protein sorting 36 homolog	- Role as part of a protein complex that functions in sorting of ubiquitinated membrane proteins during endocytosis, role in HIV trafficking and assembly	* 610903	[365]

**(303-515) Predicted (miRDB) gene targets of miR-301a-5p that is downregulated by both SARS-CoV-1 and SARS-CoV-2; gene targets are predicted to be upregulated**					
303	NR2C2, (aka TR4)	Nuclear receptor subfamily 2 group C member 2	- Orphan nuclear receptor that represses or activates transcription, can induce transcription of HIV<br>- But also suppresses hepatitis B virus core gene expression	* 601426	[366]<br>[367]
304	DHX9	DExH-box helicase 9	- Multifunctional ATP-dependent nucleic acid helicase that unwinds DNA and RNA duplexes and plays a central role in many cellular processes, role in DNA-mediated innate immunity<br>- Also hijacked by various RNA viruses, i.e. Chikungunya, to assist in replication of viral genome<br>- Involved in many critical steps in HIV-1 life cycle	* 603115	[368]<br>[369]<br>[370]
305	NFYB	Nuclear transcription factor Y subunit beta	- Transcriptional regulator with a role in restricting replication of human T cell lymphotropic virus-2 in infected host cells	* 189904	[371]
306	ZEB1	Zinc finger E-box binding homeobox 1	- Transcriptional regulator with role in memory T cell survival and function<br>- Downregulated expression associated with protection against idiopathic pulmonary fibrosis<br>- Role in mediating repression of ACE2 and found to be induced by SARS-CoV-2 in lung cancer cells	* 189909	[372]<br>[373]<br>[374]
307	NAMPT	Nicotinamide phosphoribosyltransferase	- Metabolic enzyme involved in NAD biosynthesis, also a mediator of inflammation, increased in obesity, diabetes and cancer<br>- Role in macrophage interferon antiviral cascade, thus suggested augmentation as a protective host response to infection	* 608764	[375]<br>[376]
308	PNMA1	PNMA family member 1	- Neuron- and testis-specific gene, role in autoimmune paraneoplastic neurological disorders	* 604010	[377]
309	TCF3	Transcription factor 3	- Transcriptional regulator involved in neuronal differentiation, role in neuroimmune homeostasis	* 147141	[378]
310	ZFX	Zinc finger protein X-linked	- Postulated role as a transcriptional regulator for renewal of stem cells, also found to transactivate HIV-1 long terminal repeat (LTR), used by the virus to insert the genetic material into host genomes	* 314980	[379]
311	TXNIP	Thioredoxin interacting protein	- Role as a major regulator of cellular redox signaling, role in pathogenesis of various diseases	* 606599	[380]
312	ATP8A2	ATPase phospholipid transporting 8A2	- Role in maintaining asymmetry in membrane lipids, required for normal auditory and visual function	* 605870	[381]
313	RECK	Reversion inducing cysteine rich protein with kazal motifs	- Plays key role in Wnt7 signaling, dysregulated expression linked to Epstein-Barr virus infection	* 605227	[382]
314	RAB5A	RAB5A, member RAS oncogene family	- Role in intracellular membrane trafficking, role in regulation of T cell motility	* 179512	[383]
315	ANKS1B	Ankyrin repeat and sterile alpha motif domain containing 1B	- Predominately expressed in brain and testis, role in brain development and in pathogenesis of Alzheimer's disease, one of the host genes that is a viral integration site for hepatitis B virus	* 607815	[384]
316	EIF2S2	Eukaryotic translation initiation factor 2 subunit beta	- Functions in the early steps of protein synthesis, role in reactivation of pseudorabies virus	* 603908	[385]
317	XPO6	Exportin 6	- Role in nuclear export of actin complexes, host protein that mediates nuclear release of glycoprotein M of herpes simplex virus type 1	* 608411	[386]
318	CIAO2A	Cytosolic iron-sulfur assembly component 2A	- Mediates incorporation of iron-sulfur clusters into proteins such as viperin, an interferon-stimulated antiviral protein	* 618382	[387]
319	HNF1B	HNF1 homeobox B	- Transcription factor with role in nephron and pancreas development, activity is regulated by hepatitis B virus proteins	* 189907	[388]
320	HIC2	HIC ZBTB transcriptional repressor 2	- Host gene that is negatively associated with HIV-1 replication	* 607712	[389]<br>[390]

			- Novel dosage-dependent regulator of heart development, gene hemizygosity is linked to congenital heart disease		
321	TBC1D5	TBC1 domain family member 5	- GTPase-activating protein, plays a role in immune evasion by herpes viruses<br>- Role in cellular trafficking of human papillomavirus	* 615740	[391]<br>[392]
322	RUFY3	RUN and FYVE domain containing 3	- Role in generation of neuronal polarity and axon growth	* 611194	[393]
323	SNRNP27 (aka RY1)	Small nuclear ribonucleoprotein U4/U6.U5 subunit 27	- Role in mRNA splicing, genome-wide association with genetic susceptibility for atrial fibrillation<br>- host gene that is the proviral integration site for human T cell lymphotropic virus type 1	* 611594	[394]<br>[395]
324	PTPN12	Protein tyrosine phosphatase, non-receptor type 12	- Role in dephosphorylating a range of proteins and regulating cell signaling cascades, a key regulator in dendritic cell control of T cell-dependent immunity	* 600079	[396]
325	CALB1	Calbindin 1	- Role in buffering cytosolic calcium, gene overexpression protects the brain from transient cerebral ischemia<br>- Confers resistance to HIV-1 envelope (gp120) toxicity	* 114050	[397]<br>[398]
326	AHCYL1	Adenosylhomocysteinase like 1	- Role in regulation of several essential cellular functions, identified as a host protein that modulates virus infection, knockdown reduced herpes simplex virus-1 replication	* 607826	[399]
327	ZNF322	Zinc finger protein 322	- Role as transcriptional activator, of MAPK signaling pathways and in embryonic stem cells, identified as part of a biomarker signature of respiratory syncytial virus infection in human bronchial epithelial cells	* 610847	[151]
328	GABPA	GA binding protein transcription factor subunit alpha	- Transcription factor with a role in mitochondrial function that is a critical regulator of B lymphocyte development	* 600609	[400]
329	TRAF3	TNF receptor associated factor 3	- Role in regulation of NF-kappaB and MAPK pathways, role in innate immune response to viral infection	* 601896	[401]
330	EMC3	ER membrane protein complex subunit 3	- Role in ER-associated protein degradation, found essential for neuronal cell death induced by West Nile Virus		[402]
331	BMP2	Bone morphogenetic protein 2	- TGF-β family member, transcriptional regulator with role in bone formation, also shown to have role in HIV-1 infection<br>- Role in myocardial infarction<br>- Linked to liver disease	* 112261	[403]<br>[404]<br>[405]
332	HK2	Hexokinase 2	- Essential role in glycolysis, catalyzing the first step in glucose metabolism, expressed decreased by high-dose dexamethasone treatment<br>- Host gene expression increased by a broad array of viruses, including Dengue virus	* 601125	[406]<br>[407]
333	CDH17	Cadherin 17	- Calcium-dependent cell adhesion protein, role in long-term survival of memory B cells	* 603017	[408]
334	GPR35	G protein-coupled receptor 35	- Acts as a receptor for kynurenic acid, gene activation protects against cerebral ischemia<br>- However, suppression of GPR35 protects the heart from myocardial infarction	* 602646	[409]<br>[410]
335	FFAR2, (aka GPR43)	Free fatty acid receptor 2	- Role in regulation of whole-body energy homeostasis and intestinal immunity, role in inducing α-defensin from intestinal Paneth cells and contributing to enteric innate immunity<br>- On the other hand, FFAR2 is a host factor coopted by influenza virus for virus entry into host cells	* 603823	[411]<br>[412]
336	SGMS1	Sphingomyelin synthase 1	- Brain protein, role in B cell activation and lupus-like autoimmunity<br>- Role in infection and transport of several viruses, including Japanese encephalitis virus	* 611573	[413]<br>[414]

337	CCM2	CCM2 scaffold protein	- Role in heart and vessel formation and integrity, potential biomarker of chronic lung inflammatory	* 607929	[***415***]
338	CCNT2	Cyclin T2	- Cell cycle regulator, role in HIV viral replication	* 603862	[***416***]
339	PTGFR	Prostaglandin F receptor	- G-protein coupled prostaglandin receptor with role in parturition, possible role in preterm labor associated with viral infections	* 600563	[***417***]
340	RRAS2	RAS related 2	- Small GTPase that regulates multiple cellular processes, identified as a common retrovirus insertion site and possible oncogene, overexpression may lead to tumorigenesis	* 600098	[***418***]
341	MASP1	Mannan binding lectin serine peptidase 1	- Functions in lectin pathway of complement, key role in innate immunity, role in mobilization of hematopoietic stem cells as part of innate immune response to injury and infection<br>- Induced by hepatitis C virus (HCV) infection and has proinflammatory role in accelerating fibrosis progression in HCV liver disease	* 600521	[***419***]<br>[***420***]
342	RAB1A	RAB1A, member RAS oncogene family	- Regulator of intracellular membrane trafficking, proviral host factor coopted for virus assembly (herpes simplex virus 1)<br>- Role in vaccinia virus replication<br>- Required for assembly of classical swine fever virus<br>- Increased expression along with ACE2 in lungs of Covid-19 patients<br>- Expression of Rab1a and other genes associated with Covid-19 outcome shown to be decreased by treatment with Irisin, an exercise-induced myokine which is the cleavage product of the fibronectin type III domain containing 5 (FNDC5) gene	* 179508	[***421***]<br>[***422***]<br>[***423***]<br>[***424***]<br>[***425***]
343	PITPNB	Phosphatidylinositol transfer protein beta	- Host cytoplasmic protein that is essential for Aichi virus (positive-strand RNA picornavirus) replication	* 606876	[***426***]
344	ERLIN1	ER lipid raft associated 1	- Role in degradation of IP3 receptors, regulation of cholesterol homeostasis, host factor required for efficient hepatitis C virus infection	* 611604	[***427***]
345	BDP1	B double prime 1, subunit of RNA polymerase III transcription initiation factor IIIB	- Role as activator of RNA polymerase III transcription, induced by Epstein-Barr virus to stimulate expression of viral genes	* 607012	[***428***]
346	TNPO1, (aka TRN)	Transportin 1	- Role in nuclear protein import, along with other nuclear proteins, required for interferon-mediated antiviral functions, i.e. HIV restriction<br>- On the other hand, also found to bind to capsid protein of HIV and mediate viral nuclear import by triggering uncoating (timely release of the viral genome from the capsid)	* 189880	[***429***]<br>[***430***]
347	OSBPL3	Oxysterol binding protein like 3	- Intracellular lipid receptor which is also involved in regulation of the actin cytoskeleton, overexpression rescues familial amyotrophic lateral sclerosis phenotype<br>- In brain, consistently upregulated by glucocorticoids such as dexamethasone	* 606732	[***431***]<br>[***432***]
348	GTF3C2	General transcription factor IIIC subunit 2	- Required for RNA polymerase III transcription, altered expression associated with acute myocardial infarction	* 604883	[***433***]
349	DAZAP2	DAZ associated protein 2	- Role in spermatogenesis, cell signaling and transcription regulation, role in inhibiting IL-25-mediated allergic airway inflammation	* 607431	[***434***]
350	WWTR1, (aka TAZ)	WW domain containing transcription regulator 1	- Role in Hippo signaling, key role in organ size control, role in regulating alveolar mechanics and immune responses in the lung<br>- Role in pulmonary hypertension	* 607392	[***435***]<br>[***436***]<br>[***437***]

			- Role in Zika-induced microcephaly, Zika virus infection increases methylation (turns gene expression off) of WWTR1		
351	XPNPEP3	X-prolyl aminopeptidase 3	- Increased expression linked to neuropsychiatric disorders	* 613553	[*438*]
352	TOM1L1	Target of myb1 like 1 membrane trafficking protein	- Adaptor protein involved in various signaling pathways, together with CCM2, a potential biomarker of chronic lung inflammatory diseases	* 604701	[*415*]
353	PPP2R2D	Protein phosphatase 2 regulatory subunit Bdelta	- Role in cell cycle, mitosis entry and exit, gene knockdown inhibits T cell apoptosis and enhances T cell proliferation in immunosuppressive tumors	* 613992	[*439*]
354	PLPPR1, (aka PRG-3)	Phospholipid phosphatase related 1	- Role in brain development and neuronal plasticity	* 606814	[*440*]
355	UBE2L6	Ubiquitin conjugating enzyme E2 L6	- Part of cellular complex that targets proteins for degradation, role in interferon's regulation of host antiviral response	* 603890	[*441*]
356	GLRX5	Glutaredoxin 5	- Required for iron homeostasis and regulation of hemoglobin synthesis, role in protection against heart failure	* 609588	[*442*]
357	PPP2R5C	Protein phosphatase 2 regulatory subunit B'gamma	- Role in the negative control of cell growth and division, host protein that is hijacked by human T cell leukemia virus type 1 to help in integrating viral DNA into host chromosomes	* 601645	[*443*]
358	HMGN2, (aka HMG17)	High mobility group nucleosomal binding domain 2	- Role in maintaining an open chromatin configuration around transcribable genes, antiviral role in inhibiting hepatitis B virus expression and replication	* 163910	[*444*]
359	SSR3	Signal sequence receptor subunit 3	- Role in protein translocation across the ER membrane, upregulated in activated natural killer cells as part of an enhanced innate immune response	* 606213	[*445*]
360	SLC44A1, (aka CTL1)	Solute carrier family 44 member 1	- Choline transporter with role in membrane synthesis, involved in phospholipid production for the generation of lung surfactants, role in response to viral infections and asthma in bronchial epithelial cells	* 606105	[*446*]
			- Important role in macrophage innate immune response		[*447*]
			- Role in hepatitis C virus infection		[*448*]
361	CD84	CD84 molecule	- Immunoglobulin gene with role in regulation of innate and adaptive immune responses, role in activation of T cells.	* 604513	[*449*]
362	B4GALT5	Beta-1,4-galactosyltransferase 5	- Role in neuronal maturation and axonal and myelin formation, expression positively correlated with diabetes and obesity, downregulation alleviates insulin resistance and reduces inflammation	* 604016	[*450*]
363	CPB2	Carboxypeptidase B2	- Cleaves C-terminal amino acid residues from biologically active peptides, possible role as a biomarker of liver fibrosis in chronic hepatitis B infection	* 603101	[*451*]
364	DHX32	DEAH-box helicase 32 (putative)	- Novel RNA helicase with role in regulating T cell response to apoptotic stimuli	* 607960	[*452*]
			- Possibly dysregulated in diabetic neuropathy		[*453*]
365	HIF1A	Hypoxia inducible factor 1 subunit alpha	- Master transcriptional regulator of adaptive response to hypoxia, possible role in virus-induced metabolic changes that lead to type 2 diabetes	* 603348	[*454*]
366	ATG7	Autophagy related 7	- Role in autophagy and mitophagy, inhibits inflammation, role in natural killer cell development and innate immunity	* 608760	[*455*]
			- Role in protection against Zika virus infection via type 1 interferon signaling		[*456*]
367	CLCN6	Chloride voltage-gated channel 6	- Gene variants associated with cardiac dysfunction	* 602726	[*457*]

368	ZNF347	Zinc finger protein 347	- Role in transcriptional regulation, identified by genome-wide association studies as a susceptibility gene for coronary artery disease		[*458*]
369	BRD3	Bromodomain containing 3	- Role in chromatin remodeling and transcription regulation, promotes innate immune response in macrophages via increasing type 1 interferon production	* 601541	[*459*]
370	ZNF268	Zinc finger protein 268	- Role as transcriptional repressor, positive role in regulation of virus-induced (Sendai virus/vesicular stomatitis virus) pro-inflammatory cytokine production	* 604753	[*460*]
371	PTPN14	Protein tyrosine phosphatase, non-receptor type 14	- Role in variety of cellular processes including cell growth, host cellular protein that is inactivated by human papillomavirus (HPV) proteins to enable HPV-mediated oncogenesis	* 603155	[*461*]
372	ITCH	Itchy E3 ubiquitin protein ligase	- Role in protein degradation, role in many aspects of immune response including T cell activation and T helper cell differentiation	* 606409	[*462*]
373	IRS2	Insulin receptor substrate 2	- Role in mediating effects of insulin on various cellular processes, hypophosphorylation implicated in insulin resistance induced by hepatitis C virus infection	* 600797	[*463*]
374	BCAT1	Branched chain amino acid transaminase 1	- Role in catabolism of essential branch chain amino acids (leucine, isoleucine, valine), associated with metabolic programming in activated macrophages and with inflammatory diseases<br>- Role in antiviral interferon responses	* 113520	[*464*]<br>[*465*]
375	GPR158	G protein-coupled receptor 158	- Orphan receptor associated with stress-induced depression<br>- Gene variant has possible role in spontaneous clearance of hepatitis C virus	* 614573	[*466*]<br>[*467*]
376	SSTR2	Somatostatin receptor 2	- Role in regulating release of many hormones and secretory proteins, including those involved in immune responses	* 182452	[*468*]
377	ELOVL6	ELOVL fatty acid elongase 6	- Role in synthesis of long-chain fatty acids, role in insulin resistance and diabetes (deficiency improves glycemic control)	* 611546	[*469*]
378	CD80	CD80 molecule	- Role in T cell production and cytokine production, role in immune response to various viruses, including Ebola	* 112203	[*470*]
379	VAV3	Vav guanine nucleotide exchange factor 3	- Role in pathways leading to cytoskeletal and transcriptional alterations, part of an immune signature for gliomas	* 605541	[*471*]
380	ALDH1A3	Aldehyde dehydrogenase 1 family member A3	- Role in estrogen regulation of metabolism, possible role in sex-specific differences in metabolic diseases and inflammation and certain cancers	* 600463	[*472*]
381	SHISA6	Shisa family member 6	- Role in maintenance of high-frequency synaptic transmission	* 617327	[*473*]
382	SHMT2	Serine hydroxymethyltransferase 2	- Role in intracellular glycine synthesis, role in type 1 interferon signaling	* 138450	[*474*]
383	PLEKHA8 (aka FAPP2)	Pleckstrin homology domain containing A8	- Role as cargo transport protein, host factor coopted for hepatitis C virus infectivity and genome replication	* 608639	[*475*]
384	CUL3	Cullin 3	- Role in protein degradation, component of E3 ubiquitin ligase, hijacked and repurposed by rotavirus to antagonize host innate immune responses by degrading cellular proteins crucial for interferon expression<br>- Also a negative regulator of HIV-1 transcription	* 603136	[*476*]<br>[*477*]
385	NAB1	NGFI-A binding protein 1	- Role as transcriptional repressor, involved in interferon and epidermal growth factor signaling, implicated in immune-mediated inflammatory diseases	* 600800	[*478*]

386	ZFYVE1	Zinc finger FYVE-type containing 1	- Role in membrane trafficking and formation of lipid droplets, negative regulator of melanoma differentiation-associated gene 5 (MDA5)-mediated innate antiviral response	* 605471	[479]
387	TICAM2	Toll like receptor adaptor molecule 2	- Role in type 1 interferon signaling<br>- Host gene shown to contribute to SARS-CoV pathogenesis	* 608321	[480]<br>[481]
388	TCF7	Transcription factor 7	- Involved in T cell lymphocyte differentiation and antiviral immunity	* 189908	[482]
389	RBFOX1	RNA binding fox-1 homolog 1	- Recurrent targeted gene of hepatitis B virus<br>- Role in synaptic function	* 605104	[76]<br>[483]
390	PLEKHM1	Pleckstrin homology and RUN domain containing M1	- Part of autophagy machinery hijacked by coxsackievirus to enhance viral replication	* 611466	[484]
391	TBL1XR1	Transducin beta like 1 X-linked receptor 1	- Role in transcriptional activation, B cell function	* 608628	[485]
392	SLFN11	Schlafen family member 11	- Interferon-induced inhibitor of DNA replication, inhibits retrovirus protein synthesis	* 614953	[486]
393	ZXDC	ZXD family zinc finger C	- Role in transcription of major histocompatibility complex genes, role in interferon-γ production following tuberculous infection	* 615746	[487]
394	GCNT1	Glucosaminyl (N-acetyl) transferase 1	- A key enzyme in glycan biosynthesis, role in modulation of immunity and disease, Gcnt1 deficient mice show bone marrow, blood and lung neutrophilia	* 600391	[488]
395	SEMA5A	Semaphorin 5A	- Role in axon guidance, involved in immune cell regulation and implicated in autoimmune disorders	* 609297	[489]
396	PLPPR4 (aka PRG-1)	Phospholipid phosphatase related 4	- Role in axon outgrowth and regenerative sprouting	* 607813	[490]
397	VANGL2	VANGL planar cell polarity protein 2	- Role in morphogenesis and cell polarity, role in Epstein-Barr virus-induced gastric carcinoma	* 600533	[491]
398	ETS2	ETS proto-oncogene 2, transcription factor	- Transcriptional activator in development and apoptosis, transcriptional repressor of HIV-1	* 600533	[492]
399	CDC23	Cell division cycle 23	- Role in cell cycle progression, role in pathogenesis of HTLV-1 and HCV and chikungunya	* 603462	[323]
400	MLLT11	MLLT11, transcription factor 7 cofactor	- Role in lymphoid development, promotes T cell differentiation	* 604684	[493]
401	SYT17	Synaptotagmin 17	- Role in synaptic physiology		[494]
402	ZNF267	Zinc finger protein 267	- Transcriptional regulator, represses MMP-10	* 604752	[495]
403	RHEB	Ras homolog, mTORC1 binding	- Role in regulating growth and cell cycle progression, role in innate immunity	* 601293	[496]
404	EPHB2	EPH receptor B2	- Role in axon guidance during development, implicated in platelet activation and blood coagulation, role in HIV-1 induced neurocognitive effects	* 600997	[497]
405	TMEM237	Transmembrane protein 237	- Cancer-related gene that is the host integration site for human papillomavirus	* 614423	[498]
406	SEZ6L	Seizure related 6 homolog like	- Implicated in cardiovascular comorbidities	* 607021	[499]
407	EIF4E2	Eukaryotic translation initiation factor 4E family member 2	- Implicated in coronavirus-host protein-protein interactions	* 605895	[500]
408	SNTG1	Syntrophin gamma 1	- Role in subcellular protein localization, gene variant implicated in arterial hypertension	* 608714	[501]
409	CACNA1E	Calcium voltage-gated channel subunit alpha1 E	- Role in entry of calcium into excitable cells and in a variety of calcium-dependent processes, role in porcine deltacoronavirus replication	* 601013	[502]
410	ZPR1	ZPR1 zinc finger	- Role in communicating growth signals from cytoplasm to nucleus, gene variant associated with risk of cardiovascular disease	* 603901	[503]
411	RIPOR2	RHO family interacting cell polarization regulator 2	- Role in immune-related functions	* 611410	[504]
412	KLF12	Kruppel like factor 12	- Transcriptional regulator, gene variant associated with differential viral clearance and adverse response to interferon-ribavirin treatment in HCV patients	* 607531	[505]

413	MOBP	Myelin-associated oligodendrocyte basic protein	- Role in stabilizing the myelin sheath, role in immune response to viral infection	* 600948	[506]
414	ZNF189	Zinc finger protein 189	- Predicted to be one of the viral integration sites for human papillomavirus	* 603132	[507]
415	TRIM39	Tripartite motif containing 39	- An E3 ubiquitin ligase with potential role in interferon-inducible responses	* 605700	[508]
416	AAK1	AP2 associated kinase 1	- Regulates endocytosis, pathological role in colon cancer and in coronavirus infection<br>- Also implicated in RSV, rabies virus and dengue virus infections	* 616405	[509]<br>[510]
417	SEMA4A	Semaphorin 4A	- Role in cell-cell signaling and immunomodulation, role in regulatory T cell expansion and protection against viral bronchiolitis	* 607292	[511]
418	MXD1	MAX dimerization protein 1	- Transcriptional repressor and hub gene involved in immune function<br>- Role in pathogenesis of avian influenza viruses	* 600021	[512]<br>[513]
419	SPAG8	Sperm associated antigen 8	- Role in human papillomavirus-induced cancers	* 605731	[514]
420	TUSC2	Tumor suppressor 2, mitochondrial calcium regulator	- Role in modulating inflammatory responses and mitochondrial function	* 607052	[515]
421	HIF3A	Hypoxia inducible factor 3 subunit alpha	- Role in type 1 interferon responses, development of immune system and lupus nephritis	* 609976	[516]
422	AP2B1	Adaptor related protein complex 2 subunit beta 1	- Host factor coopted for role in influenza A virus replication	* 601025	[412]
423	JMJD8	Jumonji domain containing 8	- Role in TNF-induced signaling		[517]
424	GFRA2 (aka Neurturin Receptor Alpha)	GDNF family receptor alpha 2	- Role in dampening allergic inflammation	* 601956	[518]
425	GPR39	G protein-coupled receptor 39	- Host factor coopted for hepatitis B virus proliferation, potential druggable target for antiviral therapies	* 602886	[519]
426	KPNB1	Karyopherin subunit beta 1	- Host innate immune response factor with role in nuclear protein import, this function disrupted by SARS-CoV to facilitate viral infection	* 602738	[520]
427	PPARA	Peroxisome proliferator activated receptor alpha	- Key regulator of lipid metabolism, pro-viral role in promoting viral replication (HBV, HCV)	+ 170998	[521]
428	FABP3	Fatty acid binding protein 3	- Positive regulator of B cell activation		[522]
429	CAMK1D	Calcium/calmodulin dependent protein kinase ID	- Role in CREB-dependent transcription, role in immune response of dendritic cells to influenza virus	* 607957	[523]
430	MED14	Mediator complex subunit 14	- Role in RNA polymerase II transcriptional machinery, role in vitamin D-mediated stimulation of glucocorticoid anti-inflammatory effects in human monocytes<br>- Role in HIV-1 transcription	* 300182	[524]<br>[525]
431	PMEPA1	Prostate transmembrane protein, androgen induced 1	- Role in immunosuppression	* 60656	[526]
432	ZDHHC21	Zinc finger DHHC-type containing 21	- Role in modifying and regulating sex steroid hormone receptors, role in mediating endothelial dysfunction in systemic inflammatory response syndrome	* 614605	[527]
433	GLIPR2 (aka GAPR-1)	GLI pathogenesis related 2	- Role in enhancing type 1 interferon signaling<br>- Possible role in diabetic neuropathy	* 607141	[528]<br>[529]
434	MDM4, DM4	p53 regulator	- Regulator of p53, possible neuroprotective role in preventing HIV-induced neuronal damage	* 602704	[530]
435	PLCH1	Phospholipase C eta 1	- Dysregulated expression implicated in liver damage	* 612835	[531]
436	KCND2 (aka KV4.2)	Potassium voltage-gated channel subfamily D member 2	- Dysregulated expression linked to acute myocardial infarction	* 605410	[532]
437	SSH2	Slingshot protein phosphatase 2	- Role in neutrophil chemotaxis	* 606779	[533]
438	KCNIP1	Potassium voltage-gated channel interacting protein 1	- Genetic association with risk of coronary artery disease	* 604660	[534]
439	NAA50	N(alpha)-acetyltransferase 50, NatE catalytic subunit	- Role in T lymphocyte function	* 610834	[535]
440	EGR1	Early growth response 1	- Role in promoting systemic inflammatory responses	* 128990	[536]

			- Role as host factor that promotes viral latency (human cytomegalovirus)		[537]
441	KAT6A	Lysine acetyltransferase 6A	- Role in determining memory T cell diversity during immune responses	* 601408	[538]
442	TCF12 (aka HTF4)	Transcription factor 12	- Role in neuronal differentiation, regulates gene expression in B and T cells, role in HIV-1 gene expression	* 600480	[539]
443	SLC7A11	Solute carrier family 7 member 11	- Role as transporter, has antioxidant activity, host factor involved in VSV infection	* 607933	[540]
444	SEC24C	SEC24 homolog C, COPII coat complex component	- Role in vesicle trafficking, host factor used in Ebola virus intracellular transport<br>- Key host factor that regulates entry of hepatitis C virus	* 607185	[541]<br>[542]
445	KITLG (aka stem cell factor)	KIT ligand	- Essential role in cell survival, proliferation, development, differential role in hematopoietic progenitor cell homeostasis in HIV patients treated with antiretroviral drugs	* 184745	[543]
446	HSPD1	Heat shock protein family D (Hsp60) member 1	- Chaperonin implicated in mitochondrial protein import and macromolecular assembly, altered expression associated with age-related changes that impact Covid-19 patients	* 118190	[544]
447	ZNF566	Zinc finger protein 566	- Transcriptional regulator with potential role in atrial fibrillation-related stroke		[545]
448	AR	Androgen receptor	- Multifunctional steroid hormone receptor with roles in regulating gene expression in target tissues, role in male susceptibility to Covid-19	* 313700	[546]
449	CASR	Calcium sensing receptor	- Role in sensing extracellular calcium in most tissues, role in modulating immune response	* 601199	[547]
450	ZNF721	Zinc finger protein 721	- Possible host integration site for HIV-1		[548]
451	SYNPO2	Synaptopodin 2	- Possible role in HIV-associated lung cancer		[549]
452	NKIRAS2	NFKB inhibitor interacting Ras like 2	- Role in Epstein-Barr virus associated nasopharyngeal carcinoma<br>- Potential target for cardiac repair following myocardial injury	* 604497	[550]<br>[551]
453	FLRT3	Fibronectin leucine rich transmembrane protein 3	- Part of biomarker signature of human papillomavirus-associated cancers	* 604808	[552]
454	SKP2	S-phase kinase associated protein 2	- Part of ubiquitin protein ligase complex involved in protein degradation, role in B cell function<br>- Host protein that is a target for new antiviral drugs (inhibition of SKP2 reduces MERS coronavirus infection)	* 601436	[553]<br>[554]
455	NFASC	Neurofascin	- Role in chronic inflammatory neuropathies<br>- Candidate gene in ischemic cardiomyopathy	* 609145	[555]<br>[556]
456	SEC14L3	SEC14 like lipid binding 3	- Expressed in airway epithelium and potential target for asthma therapies<br>- Over expression linked to hypertension and left ventricular remodeling	* 612824	[557]<br>[284]
457	FANCL	FA complementation group L	- Role in DNA damage response, role in immunity and mitophagy	* 608111	[558]
458	SMUG1	Single-strand-selective monofunctional uracil-DNA glycosylase 1	- Role in base excision repair, host target of HIV-1 protein VPR to promote viral replication	* 607753	[559]
459	NCR1	Natural cytotoxicity triggering receptor 1	- Possible role in innate immune function	* 604530	[560]
460	POTEM	POTE ankyrin domain family member M	- Associated with sudden onset type 1 diabetes	-	[561]
461	RABGEF1 (aka RABEX-5)	RAB guanine nucleotide exchange factor 1	- Role in membrane fusion and trafficking, negative regulator of type 1 interferon production	* 609700	[562]
462	PTH	Parathyroid hormone	- Role in regulating blood calcium and phosphate levels, high levels associated with severe Covid-19	* 168450	[563]

463	UBE2E3	Ubiquitin conjugating enzyme E2 E3	- Host protein required for replication of HIV-1	* 604151	[564]
464	TRAFD1	TRAF-type zinc finger domain containing 1	- Role in innate immune function	* 613197	[20]
465	FCHO2	FCH domain only 2	- Altered expression associated with heart failure	* 613438	[565]
466	MXRA7	Matrix remodeling associated 7	- Possible role in pathological processes in the eye<br>- Possible role in inflammation-induced liver injury	-	[566]<br>[567]
467	RETREG1 (aka FAM134B)	Reticulophagy regulator 1	- Role in ER membrane remodeling, host cell restriction factor for both dengue virus and Zika virus	* 613114	[568]
468	IL22RA2	Interleukin 22 receptor subunit alpha 2	- Role as IL22 antagonist in regulation of inflammatory responses, gene isoforms fine-tune IL-22 signaling like a rheostat, role in regulating progression of chronic liver disease in patients with hepatitis C virus or schistosome infections	* 606648	[569]
469	SEPTIN2	Septin 2	- Role in cytoskeletal function and cytokinesis, host protein in neural progenitor cells involved in Zika virus-induced neural toxicity	* 601506	[570]
470	CDH19	Cadherin 19	- Calcium-dependent cell adhesion protein, role in immune function	* 603016	[571]
471	RIOK3	RIO kinase 3	- Role in regulating type 1 interferon-dependent innate immune responses against DNA and RNA viruses	* 603579	[572]
472	STK4	Serine/threonine kinase 4	- Role in innate immunity and interferon production	* 604965	[573]
473	BTN2A1	butyrophilin subfamily 2 member A1	- Immunoglobulin involved in T cell immunity	* 613590	[574]
474	ELOVL7	ELOVL fatty acid elongase 7	- Role in lipid metabolism, host factor essential for human cytomegalovirus replication	* 614451	[575]
475	SRF	Serum response factor	- Transcriptional regulator of many immediate-early genes, host gene in T cells coopted in human T cell leukemia virus type 1 infection	* 600589	[576]
476	FAM129A	Family with sequence similarity 129 member A	- Implicated role in abdominal aortic aneurysm	-	[577]
477	PAN3	Poly(A) specific ribonuclease subunit PAN3	- Role in miRNA-mediated mRNA turnover, dysregulated by poliovirus infection	* 617448	[578]
478	SLC15A4	Solute carrier family 15 member 4	- Transporter activity, expressed in dendritic cells and role in controlling persistent viral infections	* 615806	[579]
479	C1orf21	Chromosome 1 open reading frame 21	- Gene locus identified as possible risk factor for cardiovascular disease	-	[580]
480	SPOPL	Speckle type BTB/POZ protein like	- Possible role in endocytic host cell entry of influenza A virus	-	[581]
481	DIXDC1	DIX domain containing 1	- Regulated by hepatitis B virus-encoded miRNA	* 610493	[582]
482	TP53INP2	Tumor protein p53 inducible nuclear protein 2	- Host factor regulated by polyomavirus BK	* 617549	[583]
483	IL1F10	Interleukin 1 family member 10	- Role in inflammation and host defense	* 615296	[584]
484	SETX	Senataxin	- Role in controlling (suppressing) the antiviral transcriptional response and viral biogenesis	* 608465	[585]
485	BTN3A2	Butyrophilin subfamily 3 member A2	- Role in T cell responses in the adaptive immune response<br>- Novel susceptibility gene for type 1 diabetes<br>- Host gene that plays a role in hepatitis C viral infection	* 613594	[586]<br>[587]<br>[588]
486	CD96	CD96 molecule	- Role in interaction of activated T and NK cells, role in viral pathogenesis	* 606037	[589]
487	CKAP4 (aka P63)	Cytoskeleton associated protein 4	- Role in H1N1 influenza virus-induced alveolar remodeling	* 618595	[590]
488	SCAF8	SR-related CTD associated factor 8	- Novel susceptibility locus for diabetic kidney disease	* 616024	[591]
489	IGF2R	Insulin like growth factor 2 receptor	- Role in progression of Covid-19	* 147280	[592]
490	PLXNA4	Plexin A4	- Role in negative regulation of T lymphocyte responses	* 604280	[593]

491	B3GLCT	Beta 3-glucosyltransferase	- add sugar molecules to proteins, role in T cell function	* 610308	[594]
492	EXO1	Exonuclease 1	- Role in DNA mismatch repair, host factor that restricts replication of HIV-1 in T cells	* 606063	[595]
493	ANKRD27	Ankyrin repeat domain 27	- Role in eosinophilic esophagitis	* 618957	[596]
494	EPOR	Erythropoietin receptor	- Role in innate antiviral immune function	* 133171	[597]
495	PLSCR2	Phospholipid scramblase 2	- Role in suppressing type 1 interferon responses	* 607610	[598]
496	DFFA	DNA fragmentation factor subunit alpha	- Role in apoptosis, role in human papillomavirus pathogenesis	* 601882	[599]
497	SGCD	Sarcoglycan delta	- Role in skeletal and cardiac muscle function, gene variants implicated in cardiovascular disease	* 601411	[600]
498	PATZ1	POZ/BTB and AT hook containing zinc finger 1	- Transcriptional repressor, role in HIV-1 infection	* 605165	[601]
499	CPS1	Carbamoyl-phosphate synthase 1	- Mitochondrial protein, non-invasive prognostic marker of chronic hepatitis C infection	* 608307	[602]
500	RNF128 (aka GRAIL)	Ring finger protein 128, E3 ubiquitin protein ligase	- Role in T cell activation<br>- Role in innate antiviral immune function	* 300439	[603]<br>[604]
501	G6PC2 (aka IGRP)	Glucose-6-phosphatase catalytic subunit 2	- Role in glucose metabolism, role in autoimmune diabetes	* 608058	[605]
502	ELOVL4	ELOVL fatty acid elongase 4	- Role in inflammatory responses	* 605512	[606]
503	RBBP4	RB binding protein 4, chromatin remodeling factor	- Role in repressing HIV-1 transcription	* 602923	[607]
504	SUCNR1 (aka GPR91)	Succinate receptor 1	- Role in immunomodulation, diabetes and cardiac hypertrophy<br>- Role in modulating pathophysiological processes in chronic diseases	* 606381	[608]<br>[609]
505	M6PR	Mannose-6-phosphate receptor, cation dependent	- Role in lysosome function, role in infection by diverse viruses such as herpesvirus<br>- And rotavirus	* 154540	[610]<br>[611]
506	TIMELESS	Timeless circadian regulator	- Role in circadian rhythms, DNA replication and genome stability, role in viral (herpesvirus) genome maintenance	* 603887	[612]
507	KNG1 (aka Bradykinin)	Kininogen 1	- Role in blood coagulation, role in hepatitis B-associated liver failure<br>- Role in pulmonary vascular endothelial injury and acute pulmonary hypertension caused by Covid-19	* 612358	[613]<br>[614]
508	RPL7L1	Ribosomal protein L7 like 1	- Possible role in myocardial/ischemia reperfusion injury	* 617417	[615]
509	ATXN1L	Ataxin 1 like	- Link to spinocerebellar ataxia 1 disease, role in lung alveolarization defects	* 614301	[616]
510	EFTUD2	Elongation factor Tu GTP binding domain containing 2	- Identified as a novel innate antiviral immune regulator of hepatitis C virus infection	* 614301	[617]
511	H6PD	Hexose-6-phosphate dehydrogenase/glucose 1-dehydrogenase	- Key role in metabolism, obesity and diabetes	* 138090	[618]
512	SLC9A1 (aka NHE1)	Solute carrier family 9 member A1	- Sodium/hydrogen antiporter that regulates pH homeostasis, targeted and downregulated by HIV-1	* 107310	[619]
513	RAP2A	RAP2A, member of RAS oncogene family	- Role in innate immune responses<br>- Upregulated by metformin to decrease insulin resistance in type 1 diabetes	* 179540	[620]<br>[621]
514	SLC1A2 (aka EAAT2)	Solute carrier family 1 member 2	- Main transporter that clears glutamate from extracellular spaces in synapses, targeted by HIV-1 and implicated in HIV-associated neurocognitive disorders	* 600300	[622]
515	MUC13	Mucin 13, cell surface associated	- Transmembrane protein involved in cell signaling, host gene differentially altered by respiratory syncytial virus infection	* 612181	[623]
**(516-691) Predicted (miRDB) gene targets of miR-4521 that is upregulated by MERS-CoV; gene targets are predicted to be downregulated**					

516	RRAS2	RAS related 2	- Role in activating signal transduction pathways involved in cell proliferation, role in metabolic programming of B cells	* 600098	[*624*]
517	KLF6	Kruppel like factor 6	- Role in B cell growth and development, role in promoting macrophage inflammatory and hypoxia response<br>- Role in viral infection<br>- Associated with diabetes mellitus	* 602053	[*625*]<br>[*626*]<br>[*627*]
518	GABARAPL2 (aka ATG8)	GABA type A receptor associated protein like 2	- Role in autophagy and mitophagy, host protein coopted for promoting viral pathogenesis	* 607452	[*628*]
519	UBE2C	ubiquitin conjugating enzyme E2 C	- Possible role in pathogenesis of hepatitis C virus-induced hepatocellular carcinoma	* 605574	[*629*]
520	VPS13C	Vacuolar protein sorting 13 homolog C	- Role in mitochondrial function, potential prognostic biomarker of early dengue virus infection	* 608879	[*630*]
521	TENM1	Teneurin transmembrane protein 1	- Signal transduction protein involved in neural development and olfaction, implicated in congenital anosmia (inability to smell), loss-of-function mutations linked to congenital general anosmia	* 300588	[*631*]
522	HIPK2	Homeodomain interacting protein kinase 2	- Transcriptional regulator, essential for type 1 interferon-mediated antiviral immunity	* 606868	[*632*]
523	FOXM1	Forkhead box M1	- Transcription factor involved in DNA replication and mitosis, role in human papillomavirus infection	* 602341	[*633*]
524	NCOA2	Nuclear receptor coactivator 2	- Transcriptional coactivator, role in herpesvirus lytic reactivation	* 601993	[*634*]
525	CDC37L1	Cell division cycle 37 like 1	- Role in hepatitis B virus-associated hepatocellular carcinoma	* 610346	[*635*]
526	LRRC19	Leucine rich repeat containing 19	- Pathogen recognition receptor that induces proinflammatory cytokine expression, promotes gut inflammation,	* 619068	[*636*]
527	PABPC5	Poly(A) binding protein cytoplasmic 5	- X-linked gene implicated in immune function and inflammation and Crohn's disease	* 300407	[*637*]
528	BCL11A	BCL11A, BAF complex component	- Transcriptional regulator, role in dendritic cell development	* 606557	[*638*]
529	ACAA2	Acetyl-CoA acyltransferase 2	- Role in HIV-1 pathogenesis	* 604770	[*639*]
530	CENPF	Centromere protein F	- Role in chromosome segregation, role in viral (herpesvirus) genome tethering and persistence	* 600236	[*640*]
531	SH2D4B	SH2 domain containing 4B	- Role in T cell antigen receptor signal transduction<br>- Gene locus implicated in cardiomyopathy and myodegenerative disorders	-	[*641*]<br>[*642*]
532	ATG3	Autophagy related 3	- Role in autophagy, role in pathogenesis of various viruses including influenza A virus-induced apoptosis and replication	* 609606	[*643*]
533	COPA	Coatomer protein complex subunit alpha	- Loss of function leads to enhanced type 1 interferon signaling	* 601924	[*644*]
534	TRABD	TraB domain containing	- Role in Epstein-Barr virus-associated gastric cancer	-	[*645*]
535	CPSF6	cleavage and polyadenylation specific factor 6	- Role in pre-RNA processing, host factor that plays a key role in HIV-1 infection	* 604979	[*646*]
536	PPP1R16B (aka TIMAP)	Protein phosphatase 1 regulatory subunit 16B	- Positive regulator of pulmonary endothelial cell barrier function	* 613275	[*647*]
537	ZBTB18 (aka RP58)	Zinc finger and BTB domain containing 18	- Transcriptional repressor, role in brain development and function, reduced RP58 levels involved in cognitive function impairment	* 608433	[*648*]
538	KLHL42	Kelch like family member 42	- Ubiquitin E3 ligase implicated in development of pulmonary fibrosis in systemic scleroderma, an autoimmune disease	* 618919	[*649*]
539	MBNL1	Muscleblind like splicing regulator 1	- Regulates pre-mRNA alternative splicing, loss of function linked to RNA misprocessing in thymus which is essential for T lymphocyte maturation	* 606516	[*650*]
540	EIF2AK3	Eukaryotic translation initiation factor 2 alpha kinase 3	- Metabolic stress-sensing protein kinase involved in unfolded protein response, a potential target for Covid-19 therapeutics	* 604032	[*651*]

541	NEDD4L	Neural precursor cell expressed, developmentally down-regulated 4-like, E3 ubiquitin protein ligase	- Host protein involved in replication of various viruses, including enterovirus 71	* 602278	[652]
542	TTC39A	Tetratricopeptide repeat domain 39A	- Novel potential type 2 diabetes gene mediating β cell loss and hyperglycemia	-	[653]
543	WAPL	WAPL cohesin release factor	- Role in mitosis, host protein involved in hepatitis C virus infection	* 610754	[654]
544	TAF5L	TATA-box binding protein associated factor 5 like	- Candidate gene for type 1 diabetes	* 612116	[655]
545	KLF5	Kruppel like factor 5	- Transcription factor, role in regulation of antiviral interferon-induced transmembrane proteins during H5N1 virus infection	* 602903	[656]
546	DOCK5	Dedicator of cytokinesis 5	- Role in regulation of intestinal epithelial cell spreading and migration, host factor implicated in promoting influenza virus infection, CRISPR knockout of DOCK5 reduced influenza virus replication	* 616904	[657]
547	ENO1	Enolase 1	- Glycolytic enzyme, with role in immunoglobulin production, one of several hub genes implicated in nonischemic cardiomyopathy<br>- Role in hepatitis B virus replication	* 172430	[658]<br>[659]
548	DENND1B	DENN domain containing 1B	- Role in regulation of cytokine production in TH2 lymphocytes	* 613292	[660]
549	CPNE4	Copine 4	- Gene variant implicated in coronary artery disease	* 604208	[661]
550	ABR	ABR, RhoGEF and GTPase activating protein	- Loss-of-function linked to pathogenesis of pulmonary hypertension	* 600365	[662]
551	FBXO28	F-box protein 28	- Role in protein ubiquination, genetic silencing of FBXO28 impairs pancreatic β-cell survival in diabetes	* 609100	[663]
552	KIT	KIT proto-oncogene receptor tyrosine kinase	- Cytokine receptor that plays essential roles in cell survival and proliferation, similar roles in various viral infections, including hepatitis C virus	* 164920	[664]
553	ARG1	Arginase 1	- Role in arginine metabolism which is a critical regulator of innate and adaptive immune responses, ARG1-expressing myeloid cells inhibit antiviral T cells	* 608313	[665]
554	DMGDH	Dimethylglycine dehydrogenase	- Role in catabolism of choline, deficiency linked to several metabolic diseases<br>- Deficiency linked to development of kidney disease<br>- Deficiency causally linked to development of diabetes	* 605849	[666]<br>[667]<br>[668]
555	NETO2	Neuropilin and tolloid like 2	- Role in glutamate signaling, auxiliary subunit of kainite receptors, loss-of-function linked to conditionability, a phenotype linked to PTSD	* 607974	[669]
556	ZEB2	Zinc finger E-box binding homeobox 2	- Role as transcriptional repressor, attenuates inflammation via suppression of NF-κB signaling<br>- Role in regulating fate of memory CD8+ T cells<br>- Host restriction factor degraded by hepatitis B virus regulatory HBx protein to promote viral replication	* 605802	[670]<br>[372]<br>[671]
557	WWOX	WW domain containing oxidoreductase	- Tumor suppressor with role in apoptosis, expression repressed by viral oncogene, HTLV-1 Tax https://pubmed.ncbi.nlm.nih.gov/21115974/,<br>- Loss of expression in lung linked to lung inflammation	* 605131	[672]<br>[673]
558	WNT10B	Wnt family member 10B	- Role in immune function, role in modulation of airway allergic response, loss-of-function leads to activation of CD4+ T helper 2 cells	* 601906	[674]
559	CDH8	Cadherin 8	- One of the host genes whose methylation profile may be a predictive marker of human papillomavirus-induced cervical cytopathology	* 603008	[675]
560	HNRNPA2B1	Heterogeneous nuclear ribonucleoprotein A2/B1	- RNA binding protein, role in host defense and inflammatory response	* 600124	[676]

			- Role in recognizing viruses and amplifying interferon production		[677]
561	GUCY1A1 (aka GUCY1A3)	Guanylate cyclase 1 soluble subunit alpha 1	- Part of the guanylate cyclase enzyme, gene variant associated with coronary artery disease risk	* 139396	[678]
562	ELAVL2	ELAV like RNA binding protein 2	- RNA-binding protein that binds to 3'UTR, host factor altered by human papillomavirus (HPV) which may be a biomarker for high-risk HPV-induced carcinogenesis	* 601673	[679]
563	MPIG6B	Megakaryocyte and platelet inhibitory receptor G6b	- Immunoglobulin family member that inhibits platelet aggregation	* 606520	[680]
564	ST8SIA4	ST8 alpha-N-acetyl-neuraminide alpha-2,8-sialyltransferase 4	- Role in modifying neural cell adhesion molecule and also role in modulating dendritic cell-T lymphocyte interactions	* 602547	[681]
565	PCDHA9	Protocadherin alpha 9	- Risk locus for congenital heart disease	* 606315	[682]
566	STXBP1	Syntaxin binding protein 1	- Role in release of neurotransmitters, also role in cytotoxicity of natural killer and CD8+ T cells	* 602926	[683]
567	KLHL14	Kelch like family member 14	- Role in regulating B cell receptor-dependent NF-κB signaling	* 613772	[684]
568	PTGR2	Prostaglandin reductase 2	- Role in metabolism of prostaglandins, loss-of-function modulates systemic inflammation and survival in sepsis	* 608642	[685]
569	MUC17	Mucin 17, cell surface associated	- Identified as putative anti-infection peptide, with bioactivity against HIV, HPV and bacterial infections	* 608424	[686]
570	GLIPR1	GLI pathogenesis related 1	- Host gene induced by HIV-1 to promote viral replication	* 602692	[687]
571	PDK3	Pyruvate dehydrogenase kinase 3	- Role in regulating glucose metabolism and aerobic respiration, host gene that regulates hepatitis C virus replication	* 300906	[688]
572	DCAF5	DDB1 and CUL4 associated factor 5	- Loss-of-function gene locus implicated in congenital heart defects and other disorders	* 603812	[689]
573	ZNFX1	Zinc finger NFX1-type containing 1	- Interferon-stimulated mitochondrial sensor involved in antiviral responses against RNA viruses	* 618931	[690]
574	OXCT1	3-oxoacid CoA-transferase 1	- Mitochondrial matrix protein involved in ketone body metabolism, possible role in heart failure in type 2 diabetes patients	* 601424	[691]
575	TANC2	Tetratricopeptide repeat, ankyrin repeat and coiled-coil containing 2	- Implicated in multiple comorbid neurologic disorders	* 615047	[692]
576	GIMAP4	GTPase, IMAP family member 4	- Role in thymocyte development, required for interferon-γ secretion during T helper lymphocyte differentiation<br>- Possible role in autoimmune diabetes, asthma and allergy	* 608087	[693]<br>[694]
577	CLIC2	Chloride intracellular channel 2	- Chloride intracellular channel, role in inhibiting the function of the ryanodine receptor in cardiac and skeletal muscle, mutations associated with cardiomegaly and congestive heart failure	* 300138	[695]
578	DNAH11	Dynein axonemal heavy chain 11	- Force generating protein of respiratory cilia, also linked to congenital heart disease	* 603339	[696]
579	ZFYVE16	Zinc finger FYVE-type containing 16	- Role in regulating membrane trafficking in the endosome, role in regulating proliferation of B lymphocytes	* 608880	[697]
580	RBM45	RNA binding motif protein 45	- Regulates expression of human parvovirus B19 protein	* 608888	[698]
581	MET	MET proto-oncogene, receptor tyrosine kinase	- Receptor for hepatocyte growth factor, immunosuppressive role<br>- Role in regulating/enhancing innate immune response to influenza A virus infection in human alveolar epithelial cells	* 164860	[699]<br>[700]
582	PDE5A	Phosphodiesterase 5A	- Regulates intracellular concentrations of cyclic nucleotides, important for smooth muscle	* 603310	[701]

			contraction in cardiovascular system, inhibition of gene expression can prevent cardiac hypertrophy and left ventricular dysfunction		
583	YTHDC2	YTH domain containing 2	- Possible role in facilitating hepatitis C virus genome replication	* 616530	[*702*]
584	MFN1	Mitofusin 1	- Role as mediator of mitochondrial fusion, role in antiviral defense, protein targeted for cleavage by dengue virus to promote viral infection<br>- Deficiency leads to decrease in induction of interferon-β signaling	* 608506	[*703*]<br>[*704*]
585	GPR37L1	G protein-coupled receptor 37 like 1	- Orphan receptor with postulated roles in brain and heart, deficiency associated with hypertension and left ventricular hypertrophy	* 617630	[*705*]
586	DAB2	DAB2, clathrin adaptor protein	- Role in regulating function of different immune cells<br>- Host gene involved in Ebola virus entry into target cells	* 601236	[*706*]<br>[*707*]
587	OLFM1	Olfactomedin 1	- Role in responses to olfactory stimuli, deficiency leads to defective olfaction and impaired female fertility	* 605366	[*708*]
588	TET1	Tet methylcytosine dioxygenase 1	- Role in active DNA demethylation, role in reactivation of Epstein-Barr virus from latency infection	* 607790	[*709*]
589	HDAC9	Histone deacetylase 9	- Transcriptional regulator, role in antiviral immunity<br>- Role in modulating function of T regulatory cells	* 606543	[*710*]<br>[*711*]
590	SLC36A1	Solute carrier family 36 member 1	- Proton-dependent small amino acid transporter, implicated in pathology of iminoglycinuria, defects in renal and intestinal transport of glycine and proline	* 606561	[*712*]
591	STT3A	STT3A, catalytic subunit of the oligosaccharyltransferase complex	- Host factor involved in infection by various viruses, including dengue virus	* 601134	[*713*]
592	KMT2C (aka MLL3)	Lysine methyltransferase 2C	- Histone methyltransferase, mutated in HTLV-1-associated T cell leukemia	* 606833	[*714*]
593	TLL1	Tolloid like 1	- Gene variant implicated in hepatocellular carcinoma after treatment of hepatitis C infection	* 606742	[*715*]
594	IDI1	Isopentenyl-diphosphate delta isomerase 1	- Role in interferon-regulated cell-intrinsic antiviral immunity	* 604055	[*716*]
595	CGGBP1	CGG triplet repeat binding protein 1	- Role in DNA damage repair, cytoprotective functions	* 603363	[*717*]
596	RFC5	Replication factor C subunit 5	- Role in DNA replication, role in restricting replication of orthopoxvirus	* 600407	[*718*]
597	MAPK1	Mitogen-activated protein kinase 1	- Essential component of MAP kinase signal transduction pathway, role in innate immune response<br>- Role in viral infections<br>- Role in SARS-CoV-2 pathogenesis	* 176948	[*719*]<br>[*720*]<br>[*721*]
598	TIPIN	TIMELESS interacting protein	- Role in control of DNA replication and maintenance of replication fork stability, role in maintenance of latent Epstein-Barr virus	* 610716	[*722*]
599	F2RL2 (aka PAR3)	Coagulation factor II thrombin receptor like 2	- Role in thrombin-mediated cleavage, role in hemostasis and thrombosis, implicated in pathophysiology of type 2 diabetes	* 601919	[*723*]
600	BICD2	BICD cargo adaptor 2	- Adaptor protein involved in dynein-mediated motility along microtubules, host factor that promotes trafficking and import of viral genomes, including HIV-1, during infection	* 609797	[*724*]
601	SALL4	Spalt like transcription factor 4	- Key role in maintenance and self-renewal of embryonic and hematopoietic stem cells, role in hepatocellular carcinoma associated with hepatitis B or C virus infection	* 607343	[*725*]
602	PDE1C	Phosphodiesterase 1C	- Role in regulating cardiomyocyte survival	* 602987	[*726*]
603	UNC5B	UNC-5 netrin receptor B	- Role in axon guidance, role in hypoxia-induced atherosclerosis and inflammation	* 607870	[*727*]

604	SGO1	Shugoshin 1	- Role in chromosome cohesion during mitosis, function impaired by viral infection	* 609168	[728]
605	MEGF10	Multiple EGF like domains 10	- Role in cell adhesion, motility and proliferation, gene deficiency causes early onset myopathy, areflexia, respiratory distress and dysphagia	* 612453	[729]
606	NT5DC1	5'-nucleotidase domain containing 1	- Role in susceptibility to COPD	-	[344]
607	GP6	Glycoprotein VI platelet	- Role in platelet adhesion and activation, role in viral infections, implicated in pathophysiology of Covid-19	* 605546	[730]
608	FBXO21	F-box protein 21	- Role in antiviral innate response	* 609095	[731]
609	AMOTL1	angiomotin like 1	- Component of tight junctions, host factor that is recruited by paramyxovirus M proteins to achieve particle release (budding)	* 614657	[732]
610	HSD17B4 (aka MFP-2)	Hydroxysteroid 17-beta dehydrogenase 4	- Role in peroxisomal beta-oxidation (catabolism) of fatty acids, gene knockdown upregulates pro-inflammatory proteins Cox-2 and TNF-α	* 601860	[733]
611	KCNN3 (aka SK3)	Potassium calcium-activated channel subfamily N member 3	- Possible role in mediating pathological effects of human endogenous retrovirus W family envelope gene	* 602983	[734]
612	MAOB	Monoamine oxidase B	- Role in metabolism of biogenic amines, role in smoke-induced oxidative stress and inflammation in airway epithelial cells<br>- Target of repurposed drugs for Covid-19	* 309860	[735]<br>[736]
613	TRIM38	Tripartite motif containing 38	- Role in negative regulation of innate immune and inflammatory responses<br>- Role in innate immune responses to RNA and DNA viruses	-	[737]<br>[738]
614	ACVR1C	Activin A receptor type 1C	- Role in antigen presentation by dendritic cells	* 608981	[739]
615	KCNJ3	potassium voltage-gated channel subfamily J member 3	- Gene variant involved in bradyarrhythmias and atrial fibrillation	* 601534	[740]
616	GRK5	G protein-coupled receptor kinase 5	- Role in cardiovascular and neurodegenerative disorders	* 600870	[741]
617	PRL	Prolactin	- Primary role in promoting lactation, role in regulating immune cell function, role in antiviral responses of natural killer cells	* 176760	[742]
618	TOR1AIP1 (aka LAP1)	Torsin 1A interacting protein 1	- Required for nuclear membrane integrity, absence of protein expression causally linked to cardiac failure and recessive limb-girdle muscular dystrophy	* 614512	[743]
619	SYT10	Synaptotagmin 10	- Implicated in heart rate response to exercise	-	[744]
620	SPTBN1	Spectrin beta, non-erythrocytic 1	- Host factor required for HIV-1 infection	* 182790	[745]
621	ITGB1BP2 (aka MELUSIN)	Integrin subunit beta 1 binding protein 2	- Role as a chaperone, cardioprotective	* 300332	[746]
622	SPRED2	Sprouty related EVH1 domain containing 2	- Role in negative regulation of Ras signaling and downstream MAP kinase pathways, role in regulating influenza A virus-induced pneumonia	* 609292	[747]
623	ABCB5	ATP binding cassette subfamily B member 5	- Role in ATP-dependent transmembrane transport of diverse molecules, including drugs, host factor that plays a role in pathophysiology of human diffuse cutaneous leishmaniasis	* 611785	[748]
624	HNRNPU (aka SAFA)	Heterogeneous nuclear ribonucleoprotein U	- DNA and RNA binding protein, role in activating antiviral enhancers and super-enhancers	* 602869	[749]
625	GCSAM (aka HGAL)	Germinal center associated signaling and motility	- Role in negative regulation of lymphocyte motility, role in B cell receptor signaling	* 607792	[750]
626	PROK2	Prokineticin 2	- Role in circadian rhythms of behavior, role in Merkel cell polyomavirus infection	* 607002	[751]
627	CRTAM	Cytotoxic and regulatory T cell molecule	- Role in regulation and activation of various T cell subsets, role in cytotoxic antiviral responses to Zika virus	* 612597	[752]

628	PPP1R14C	Protein phosphatase 1 regulatory inhibitor subunit 14C	- Potential susceptibility locus for Kawasaki disease and other cardiovascular diseases	* 613242	[753]
629	PRKD1	Protein kinase D1	- Implicated in congenital heart disease	* 605435	[754]
630	CLMP	CXADR like membrane protein	- Role in cell-cell adhesion, host factor involved in coxsackie B virus pathogenesis and implicated in acute and chronic virus-induced myocarditis	* 611693	[755]
631	PATL1	PAT1 homolog 1 processing body mRNA decay factor	- RNA-binding protein required for mRNA processing body assembly, host factor required for translation and replication of hepatitis C virus genomic RNA	* 614660	[756]
632	UFL1	UFM1 specific ligase 1	- Role in unfolded protein response (cellular stress response), key role in maintaining intestinal homeostasis and controlling gut inflammation	* 613372	[757]
633	DDX56	DEAD-box helicase 56	- Role in cellular processes involving alteration of RNA secondary structure, negative regulator of (inhibits) antiviral type 1 interferon signaling<br>- Host factor involved in infectivity of West Nile virus	* 608023	[758]<br>[759]
634	TRAF4	TNF receptor associated factor 4	- Adaptor protein involved in signal transduction from members of TNF superfamily, implicated in HIV-1 latency	* 602464	[760]
635	BTLA	B and T lymphocyte associated	- Immunoglobulin that suppresses the immune response, host factor exploited by HIV and other viruses to inhibit host immune function<br>- Recently found to have role in modulating immune function in Covid-19 patients	* 607925	[761]<br>[762]
636	CYCS	Cytochrome c, somatic	- Central component of electron transport chain in mitochondria, affected by Epstein-Barr virus infection	* 123970	[763]
637	ALG13	ALG13, UDP-N-acetylglucosaminyltransferase subunit	- Deficiency implicated in susceptibility to epilepsy	* 300776	[764]
638	ACAP2	ArfGAP with coiled-coil, ankyrin repeat and PH domains 2	- GTPase-activating protein, host factor that may interact with vaccinia virus	* 607766	[765]
639	MYH10	myosin heavy chain 10	- Role in cytokinesis and cell shape, deficiency associated with pulmonary disease	* 160776	[766]
640	COPS2	COP9 signalosome subunit 2	- Role in regulating the ubiquitin conjugation pathway, host factor implicated in influenza virus infections	* 604508	[767]
641	ZBTB3	Zinc finger and BTB domain containing 3	- Transcription factor with role in foot-and-mouth disease virus infection	-	[768]
642	ERO1β	Endoplasmic reticulum oxidoreductase 1 beta	- Deregulation implicated in pathogenesis of diabetes mellitus	* 615437	[769]
643	LPIN1	Lipin 1	- Role in controlling metabolism of fatty acids at different levels, role in insulin resistance, obesity and diabetes https://pubmed.ncbi.nlm.nih.gov/17950103/<br>- Host factor with role in hepatitis C virus replication	* 605518	[770]<br>[771]
644	GGA2	Golgi associated, gamma adaptin ear containing, ARF binding protein 2	- Role in protein sorting and trafficking, modulator of retrovirus (HIV-1) assembly and release	* 606005	[772]
645	PELO	Pelota mRNA surveillance and ribosome rescue factor	- Role in cell division and genome stability, required for high efficiency viral replication	* 605757	[773]
646	DDHD2	DDHD domain containing 2	- Role in mitochondrial integrity, loss-of-function associated with hereditary spastic paraplegia	* 615003	[774]
647	PNP	Purine nucleoside phosphorylase	- Role in catabolism of purine nucleotides, deficiency results in defective T cell and B cell immunity	* 164050	[775]
648	PDPK1	3-phosphoinositide dependent protein kinase 1	- Master kinase that regulates PI3/AKT signaling, role in host autophagy response to viral infection	* 605213	[776]
649	FBXO3	F-box protein 3	- Role in cytokine-driven inflammation<br>- Host factor coopted by viruses to suppress transcriptional upregulation of antiviral type 1 interferons	* 609089	[777]<br>[778]
650	SESTD1	SEC14 and spectrin domain containing 1	- Role in West Nile virus replication	-	[312]

651	CTNNA3	Catenin alpha 3	- Highly expressed in cardiomyocytes, possible role in allergic airway disease	* 607667	[779]
652	ELK3	ELK3, ETS transcription factor	- Negative regulator of transcription, role in inhibiting cell growth, host factor altered by adenovirus infection	* 600247	[780]
653	NAP1L4	Nucleosome assembly protein 1 like 4	- Role as histone chaperone in nucleosome assembly, host factor with role in Chikungunya virus replication	* 601651	[781]
654	NARS	Asparaginyl-tRNA synthetase	- Role in protein synthesis, also autoantigen in myositis (muscle inflammation), activates chemokine receptors on T lymphocytes and immature dendritic cells	* 108410	[782]
655	ABCA1	ATP binding cassette subfamily A member 1	- Role as lipid and cholesterol transporter (efflux), role in innate immune function<br>- Increased expression inhibits HIV infection<br>- Host factor suppressed by HIV to maintain high intracellular cholesterol levels	* 600046	[783]<br>[784]<br>[785]
656	VPS41	VPS41, HOPS complex subunit	- Neuroprotective role in neurodegenerative diseases	* 605485	[786]
657	MAPRE1 (aka EB1)	microtubule associated protein RP/EB family member 1	- Role in regulation of microtubule structures and chromosome stability, host factor that is dysregulated by HIV to impair macrophage function	* 603108	[787]
658	IL17RD	Interleukin 17 receptor D	- Role in human inflammatory diseases	* 606807	[788]
659	PRPS1	phosphoribosyl pyrophosphate synthetase 1	- Essential role in nucleotide synthesis, loss-of-function linked to hearing loss, an identified symptom of Covid-19	* 311850	[789]
660	LRRC59	Leucine rich repeat containing 59	- Role in modulating antiviral type 1 interferon signaling	* 614854	[790]
661	EIF2A	Eukaryotic translation initiation factor 2A	- Role in protein synthesis of a small number of mRNAs, role in translation of hepatitis C virus mRNA under stress conditions	* 609234	[791]
662	ERMN	Ermin	- Role in myelin formation, downregulated expression implicated in chronic inflammatory disease, i.e. multiple sclerosis	* 610072	[792]
663	FRMD3	FERM domain containing 3	- Implicated in diabetic nephropathy in type 1 diabetes	* 607619	[793]
664	EDN3	endothelin 3	- Role as vasoconstrictive peptide, possible biomarker of HIV infection	* 131242	[794]
665	ATF6	Activating transcription factor 6	- Role in activating target genes for the unfolded protein response during ER stress, such as after viral infections<br>- Involved in coronavirus-host interactions	* 605537	[795]<br>[796]
666	ELAVL4 (aka HUD)	ELAV like RNA binding protein 4	- Role in post-transcriptional regulation of mRNAs, role in Newcastle Disease Virus infections	* 168360	[797]
667	ULK2	UNC-51 like autophagy activating kinase 2	- Role in autophagy, role in dengue virus pathogenesis	* 608650	[798]
668	INA (aka TXBP-1)	Internexin neuronal intermediate filament protein alpha	- Host factor implicated in neurological disorders associated with human T cell leukemia virus	* 605338	[799]
669	SOS1	SOS Ras/Rac guanine nucleotide exchange factor 1	- Possible role in pathogenesis of Covid-19	* 182530	[800]
670	EMC6	ER membrane protein complex subunit 6	- Role in acinar cell apoptosis and inflammatory injury, possible target for treatment of pancreatic inflammatory disease, one of the recognized complications of Covid-19	-	[801]
671	SLC1A3 (aka EAAT1)	Solute carrier family 1 member 3	- Glutamate transporter, role in TLR-mediated inflammatory effects (i.e. caused by peripheral viral infections) on cognition and seizure susceptibility	* 600111	[802]
672	C20orf194	Chromosome 20 open reading frame 194	- Variant protective in patients who are being treated for hepatitis C virus infections	* 614146	[803]
673	DUSP4	Dual specificity phosphatase 4	- Role in regulating age-related decline in T cell-dependent B cell responses	* 602747	[804]
674	SELENOT	Selenoprotein T	- Possible antiviral activity	* 607912	[805]

675	PID1	phosphotyrosine interaction domain containing 1	- Possible biomarker of response to interferon gamma treatment in sepsis patients<br>- Role in regulating insulin-dependent glucose uptake	* 612930	[806]<br>[807]
676	HOXC4	Homeobox C4	- Role in regulating genes controlling lymphocytes of the T, B and NK cell lineages	* 142974	[808]
677	MPRIP	Myosin phosphatase Rho interacting protein	- Role in regulation of the actin cytoskeleton, host factor involved in human cytomegalovirus pathogenesis	* 612935	[809]
678	MECP2	Methyl-CpG binding protein 2	- Essential role in normal function of nerve cells, mutations are cause of Rett syndrome, role in regulating gene expression in microglia and macrophages in response to inflammatory stimuli<br>- Deregulated expression also implicated in pathogenesis of diverse viruses	* 300005	[810]<br>[811]
679	FGL2	Fibrinogen like 2	- Role in virus-induced fulminant hepatitis https://pubmed.ncbi.nlm.nih.gov/25200905/<br>- Target of treatments for hepatitis C virus infection	* 605351	[812]<br>[813]
680	ZNF350 (aka ZBRK1)	Zinc finger protein 350	- Antiviral role as host gene that represses HIV-1 LTR-mediated transcription	* 605422	[814]
681	DUSP3 (aka VHR)	Dual specificity phosphatase 3	- Role in negative regulation of MAP kinases, target of drugs for diverse human diseases	* 600183	[815]
682	KIF1B	Kinesin family member 1B	- Host factor with role in hepatitis B virus infection	* 605995	[816]
683	EGFR	Epidermal growth factor receptor	- Key role in many cancer types, roles in diverse viral infections, host factor with role in suppressing interferon-induced antiviral defenses	* 131550	[817]
684	RAB2B	RAB2B, member RAS oncogene family	- Role in innate immune responses	* 607466	[818]
685	TTLL4	Tubulin tyrosine ligase like 4	- Role in antiviral immunity		[819]
686	LRRC15	Leucine rich repeat containing 15	- Role in impeding adenoviral infection	-	[820]
687	RAB3A	RAB3A, member RAS oncogene family	- Role in HIV pathogenesis	* 179490	[821]
688	WDFY1	WD repeat and FYVE domain containing 1	- Deficiency impairs TLR3-mediated immune responses	* 618080	[822]
689	TXNDC5	Thioredoxin domain containing 5	- Chaperone with role in protein folding, role in diverse diseases associated with oxidative stress, role in proviral functions of the interferon-stimulated gene 15	* 616412	[823]
690	IPMK	Inositol polyphosphate multikinase	- Role in HIV infection and replication	* 609851	[824]
691	OBSL1	Obscurin like 1	- Role in regulating microtubule dynamics, role in human papillomavirus 16 infection	* 610991	[825]

S2 References

1. K. Wang *et al.*, Two Wnt genes regulate the expression levels of antimicrobial peptides during Vibrio infection in Macrobrachium nipponense. *Fish Shellfish Immunol* **101**, 225-233 (2020).
2. H. Luo *et al.*, Efnb1 and Efnb2 proteins regulate thymocyte development, peripheral T cell differentiation, and antiviral immune responses and are essential for interleukin-6 (IL-6) signaling. *J Biol Chem* **286**, 41135-41152 (2011).
3. R. Gallini, J. Huusko, S. Yla-Herttuala, C. Betsholtz, J. Andrae, Isoform-Specific Modulation of Inflammation Induced by Adenoviral Mediated Delivery of Platelet-Derived Growth Factors in the Adult Mouse Heart. *PLoS One* **11**, e0160930 (2016).
4. A. L. Chokas *et al.*, Foxp1/2/4-NuRD interactions regulate gene expression and epithelial injury response in the lung via regulation of interleukin-6. *J Biol Chem* **285**, 13304-13313 (2010).
5. A. Alizadeh, K. T. Santhosh, H. Kataria, A. S. Gounni, S. Karimi-Abdolrezaee, Neuregulin-1 elicits a regulatory immune response following traumatic spinal cord injury. *J Neuroinflammation* **15**, 53 (2018).
6. S. Mohammadi, R. R. Isberg, Yersinia pseudotuberculosis virulence determinants invasin, YopE, and YopT modulate RhoG activity and localization. *Infect Immun* **77**, 4771-4782 (2009).
7. N. R. Choudhury, G. Heikel, G. Michlewski, TRIM25 and its emerging RNA-binding roles in antiviral defense. *Wiley Interdiscip Rev RNA* **11**, e1588 (2020).
8. K. A. Metcalf Pate *et al.*, TGFbeta-Mediated Downregulation of Thrombopoietin Is Associated With Platelet Decline in Asymptomatic SIV Infection. *J Acquir Immune Defic Syndr* **65**, 510-516 (2014).
9. H. M. Tang *et al.*, Requirement of CRTC1 coactivator for hepatitis B virus transcription. *Nucleic Acids Res* **42**, 12455-12468 (2014).
10. E. Tsuru, K. Oryu, K. Sawada, M. Nishihara, M. Tsuda, Complexin 2 regulates secretion of immunoglobulin in antibody-secreting cells. *Immun Inflamm Dis* **7**, 318-325 (2019).
11. C. Fourie *et al.*, Differential Changes in Postsynaptic Density Proteins in Postmortem Huntington's Disease and Parkinson's Disease Human Brains. *J Neurodegener Dis* **2014**, 938530 (2014).
12. F. Rehren *et al.*, Induction of a broad spectrum of inflammation-related genes by Coxsackievirus B3 requires Interleukin-1 signaling. *Med Microbiol Immunol* **202**, 11-23 (2013).
13. I. Glezer, S. Rivest, Oncostatin M is a novel glucocorticoid-dependent neuroinflammatory factor that enhances oligodendrocyte precursor cell activity in demyelinated sites. *Brain Behav Immun* **24**, 695-704 (2010).
14. K. Lugardon *et al.*, Antibacterial and antifungal activities of vasostatin-1, the N-terminal fragment of chromogranin A. *J Biol Chem* **275**, 10745-10753 (2000).

15. L. Menzel *et al.*, Down-regulation of neuronal L1 cell adhesion molecule expression alleviates inflammatory neuronal injury. *Acta Neuropathol* **132**, 703-720 (2016).

16. A. Kavelaars, P. M. Cobelens, M. A. Teunis, C. J. Heijnen, Changes in innate and acquired immune responses in mice with targeted deletion of the dopamine transporter gene. *J Neuroimmunol* **161**, 162-168 (2005).

17. Z. L. Cai *et al.*, The effect of HMGA1 in LPS-induced Myocardial Inflammation. *Int J Biol Sci* **16**, 1798-1810 (2020).

18. M. Merika, A. J. Williams, G. Chen, T. Collins, D. Thanos, Recruitment of CBP/p300 by the IFN beta enhanceosome is required for synergistic activation of transcription. *Mol Cell* **1**, 277-287 (1998).

19. P. L. Chavali *et al.*, Neurodevelopmental protein Musashi-1 interacts with the Zika genome and promotes viral replication. *Science* **357**, 83-88 (2017).

20. A. Doostparast Torshizi, K. Wang, Deconvolution of Transcriptional Networks in Post-Traumatic Stress Disorder Uncovers Master Regulators Driving Innate Immune System Function. *Sci Rep* **7**, 14486 (2017).

21. A. L. Liu *et al.*, Comparative analysis of selected innate immune-related genes following infection of immortal DF-1 cells with highly pathogenic (H5N1) and low pathogenic (H9N2) avian influenza viruses. *Virus Genes* **50**, 189-199 (2015).

22. T. I. Ng *et al.*, Identification of host genes involved in hepatitis C virus replication by small interfering RNA technology. *Hepatology* **45**, 1413-1421 (2007).

23. L. Chen *et al.*, N-myc downstream-regulated gene 1 facilitates influenza A virus replication by suppressing canonical NF-kappaB signaling. *Virus Res* **252**, 22-28 (2018).

24. C. R. Ojha *et al.*, Toll-like receptor 3 regulates Zika virus infection and associated host inflammatory response in primary human astrocytes. *PLoS One* **14**, e0208543 (2019).

25. G. Pendyala, J. L. Buescher, H. S. Fox, Methamphetamine and inflammatory cytokines increase neuronal Na+/K+-ATPase isoform 3: relevance for HIV associated neurocognitive disorders. *PLoS One* **7**, e37604 (2012).

26. L. Yang *et al.*, SP-R210 (Myo18A) Isoforms as Intrinsic Modulators of Macrophage Priming and Activation. *PLoS One* **10**, e0126576 (2015).

27. A. F. Karim *et al.*, Hemophilia A Inhibitor Subjects Show Unique PBMC Gene Expression Profiles That Include Up-Regulated Innate Immune Modulators. *Front Immunol* **11**, 1219 (2020).

28. X. X. Chen *et al.*, Paralemmin-3 contributes to lipopolysaccharide-induced inflammatory response and is involved in lipopolysaccharide-Toll-like receptor-4 signaling in alveolar macrophages. *Int J Mol Med* **40**, 1921-1931 (2017).

29. E. Garcia, D. S. Nikolic, V. Piguet, HIV-1 replication in dendritic cells occurs through a tetraspanin-containing compartment enriched in AP-3. *Traffic* **9**, 200-214 (2008).

30. M. E. Olsen, J. H. Connor, Hypusination of eIF5A as a Target for Antiviral Therapy. *DNA Cell Biol* **36**, 198-201 (2017).

31. Y. Li, L. Wang, E. E. Rivera-Serrano, X. Chen, S. M. Lemon, TNRC6 proteins modulate hepatitis C virus replication by spatially regulating the binding of miR-122/Ago2 complexes to viral RNA. *Nucleic Acids Res* **47**, 6411-6424 (2019).

32. M. R. Lopez-Huertas *et al.*, Protein kinase Ctheta is a specific target for inhibition of the HIV type 1 replication in CD4+ T lymphocytes. *J Biol Chem* **286**, 27363-27377 (2011).

33. G. O. Pilarowski *et al.*, Abnormal Peyer patch development and B-cell gut homing drive IgA deficiency in Kabuki syndrome. *J Allergy Clin Immunol* **145**, 982-992 (2020).

34. M. L. DeDiego, A. Nogales, L. Martinez-Sobrido, D. J. Topham, Interferon-Induced Protein 44 Interacts with Cellular FK506-Binding Protein 5, Negatively Regulates Host Antiviral Responses, and Supports Virus Replication. *mBio* **10**, (2019).

35. W. Hao, L. Wang, S. Li, FKBP5 Regulates RIG-I-Mediated NF-kappaB Activation and Influenza A Virus Infection. *Viruses* **12**, (2020).

36. J. C. Beltra *et al.*, IL2Rbeta-dependent signals drive terminal exhaustion and suppress memory development during chronic viral infection. *Proc Natl Acad Sci U S A* **113**, E5444-5453 (2016).

37. K. M. Choi *et al.*, C1q/TNF-related protein-3 (CTRP-3) and pigment epithelium-derived factor (PEDF) concentrations in patients with type 2 diabetes and metabolic syndrome. *Diabetes* **61**, 2932-2936 (2012).

38. Y. Fan *et al.*, USP21 negatively regulates antiviral response by acting as a RIG-I deubiquitinase. *J Exp Med* **211**, 313-328 (2014).

39. E. G. Viktorova *et al.*, A Redundant Mechanism of Recruitment Underlies the Remarkable Plasticity of the Requirement of Poliovirus Replication for the Cellular ArfGEF GBF1. *J Virol* **93**, (2019).

40. A. Al-Shami *et al.*, The adaptor protein Sh2d3c is critical for marginal zone B cell development and function. *J Immunol* **185**, 327-334 (2010).

41. B. T. McCune *et al.*, Noroviruses Co-opt the Function of Host Proteins VAPA and VAPB for Replication via a Phenylalanine-Phenylalanine-Acidic-Tract-Motif Mimic in Nonstructural Viral Protein NS1/2. *mBio* **8**, e00668-00617 (2017).

42. N. Ali *et al.*, Inflammatory and oncogenic roles of a tumor stem cell marker doublecortin-like kinase (DCLK1) in virus-induced chronic liver diseases. *Oncotarget* **6**, 20327-20344 (2015).

43. Y. Bo, S. Qiu, R. P. Mulloy, M. Cote, Filoviruses Use the HOPS Complex and UVRAG To Traffic to Niemann-Pick C1 Compartments during Viral Entry. *J Virol* **94**, (2020).

44. F. Huang *et al.*, Hepatitis E virus infection activates signal regulator protein alpha to down-regulate type I interferon. *Immunol Res* **64**, 115-122 (2016).

45. J. Demaret *et al.*, Identification of CD177 as the most dysregulated parameter in a microarray study of purified neutrophils from septic shock patients. *Immunol Lett* **178**, 122-130 (2016).

46. T. Watanabe *et al.*, The hematopoietic cell-specific Rho GTPase inhibitor ARHGDIB/D4GDI limits HIV type 1 replication. *AIDS Res Hum Retroviruses* **28**, 913-922 (2012).
47. L. Kaufman *et al.*, The homophilic adhesion molecule sidekick-1 contributes to augmented podocyte aggregation in HIV-associated nephropathy. *FASEB J* **21**, 1367-1375 (2007).
48. B. Kroczynska *et al.*, Regulatory effects of SKAR in interferon alpha signaling and its role in the generation of type I IFN responses. *Proc Natl Acad Sci U S A* **111**, 11377-11382 (2014).
49. N. Suzuki *et al.*, Robust Enhancement of Lentivirus Production by Promoter Activation. *Sci Rep* **8**, 15036 (2018).
50. G. Enyindah-Asonye *et al.*, Overexpression of CD6 and PD-1 Identifies Dysfunctional CD8(+) T-Cells During Chronic SIV Infection of Rhesus Macaques. *Front Immunol* **10**, 3005 (2019).
51. V. A. van der Mark *et al.*, Phospholipid flippases attenuate LPS-induced TLR4 signaling by mediating endocytic retrieval of Toll-like receptor 4. *Cell Mol Life Sci* **74**, 715-730 (2017).
52. H. Lerat *et al.*, Hepatitis C virus induces a prediabetic state by directly impairing hepatic glucose metabolism in mice. *J Biol Chem* **292**, 12860-12873 (2017).
53. J. H. Jeong *et al.*, Over-expression of p190RhoGEF enhances B-cell activation and germinal center formation in T-cell-dependent humoral immune responses. *Immunol Cell Biol* **97**, 877-887 (2019).
54. A. Lee, S. Liu, T. Wang, Identification of novel human kinases that suppress hepatitis C virus infection. *J Viral Hepat* **21**, 716-726 (2014).
55. E. Esashi, M. Bao, Y. H. Wang, W. Cao, Y. J. Liu, PACSIN1 regulates the TLR7/9-mediated type I interferon response in plasmacytoid dendritic cells. *Eur J Immunol* **42**, 573-579 (2012).
56. W. Shen *et al.*, Host protein atlastin-1 promotes human immunodeficiency virus (HIV-1) replication. *Virol Sin* **32**, 338-341 (2017).
57. B. Monel *et al.*, Atlastin Endoplasmic Reticulum-Shaping Proteins Facilitate Zika Virus Replication. *J Virol* **93**, e01047-01019 (2019).
58. X. Xie, P. S. Liu, P. Percipalle, Analysis of Global Transcriptome Change in Mouse Embryonic Fibroblasts After dsDNA and dsRNA Viral Mimic Stimulation. *Front Immunol* **10**, 836 (2019).
59. I. Nevo-Yassaf *et al.*, Role for TBC1D20 and Rab1 in hepatitis C virus replication via interaction with lipid droplet-bound nonstructural protein 5A. *J Virol* **86**, 6491-6502 (2012).
60. D. Nachmias, E. H. Sklan, M. Ehrlich, E. Bacharach, Human immunodeficiency virus type 1 envelope proteins traffic toward virion assembly sites via a TBC1D20/Rab1-regulated pathway. *Retrovirology* **9**, 7 (2012).

61. G. L. Sica *et al.*, RELT, a new member of the tumor necrosis factor receptor superfamily, is selectively expressed in hematopoietic tissues and activates transcription factor NF-kappaB. *Blood* **97**, 2702-2707 (2001).

62. N. Abeywickrama-Samarakoon *et al.*, Hepatitis Delta Virus histone mimicry drives the recruitment of chromatin remodelers for viral RNA replication. *Nat Commun* **11**, 419 (2020).

63. A. Marteyn *et al.*, Modulation of the Innate Immune Response by Human Neural Precursors Prevails over Oligodendrocyte Progenitor Remyelination to Rescue a Severe Model of Pelizaeus-Merzbacher Disease. *Stem Cells* **34**, 984-996 (2016).

64. F. M. Collier *et al.*, Identification and characterization of a lymphocytic Rho-GTPase effector: rhotekin-2. *Biochem Biophys Res Commun* **324**, 1360-1369 (2004).

65. K. Wang *et al.*, Alkaline ceramidase 3 deficiency aggravates colitis and colitis-associated tumorigenesis in mice by hyperactivating the innate immune system. *Cell Death Dis* **7**, e2124 (2016).

66. T. Malcolm, J. Kam, P. S. Pour, I. Sadowski, Specific interaction of TFII-I with an upstream element on the HIV-1 LTR regulates induction of latent provirus. *FEBS Lett* **582**, 3903-3908 (2008).

67. E. Svedin *et al.*, A Link Between a Common Mutation in CFTR and Impaired Innate and Adaptive Viral Defense. *J Infect Dis* **216**, 1308-1317 (2017).

68. S. Glatigny, C. A. Wagner, E. Bettelli, Cutting Edge: Integrin alpha4 Is Required for Regulatory B Cell Control of Experimental Autoimmune Encephalomyelitis. *J Immunol* **196**, 3542-3546 (2016).

69. Y. C. Yang, L. K. Chang, Role of TAF4 in transcriptional activation by Rta of Epstein-Barr Virus. *PLoS One* **8**, e54075 (2013).

70. M. Rienks *et al.*, A novel 72-kDa leukocyte-derived osteoglycin enhances the activation of toll-like receptor 4 and exacerbates cardiac inflammation during viral myocarditis. *Cell Mol Life Sci* **74**, 1511-1525 (2017).

71. E. Gusho *et al.*, Murine AKAP7 has a 2',5'-phosphodiesterase domain that can complement an inactive murine coronavirus ns2 gene. *mBio* **5**, e01312-01314 (2014).

72. M. N. Krishnan *et al.*, RNA interference screen for human genes associated with West Nile virus infection. *Nature* **455**, 242-245 (2008).

73. D. Zhao *et al.*, H3K4me3 Demethylase Kdm5a Is Required for NK Cell Activation by Associating with p50 to Suppress SOCS1. *Cell Rep* **15**, 288-299 (2016).

74. T. So, N. Ishii, The TNF-TNFR Family of Co-signal Molecules. *Adv Exp Med Biol* **1189**, 53-84 (2019).

75. Y. Tang *et al.*, Macrophage scavenger receptor 1 contributes to pathogenesis of fulminant hepatitis via neutrophil-mediated complement activation. *J Hepatol* **68**, 733-743 (2018).

76. D. Ding *et al.*, Recurrent targeted genes of hepatitis B virus in the liver cancer genomes identified by a next-generation sequencing-based approach. *PLoS Genet* **8**, e1003065 (2012).

77. S. Strunze *et al.*, Kinesin-1-mediated capsid disassembly and disruption of the nuclear pore complex promote virus infection. *Cell Host Microbe* **10**, 210-223 (2011).

78. S. Ding *et al.*, STAG2 deficiency induces interferon responses via cGAS-STING pathway and restricts virus infection. *Nat Commun* **9**, 1485 (2018).

79. P. N. Paradkar, J. B. Duchemin, J. Rodriguez-Andres, L. Trinidad, P. J. Walker, Cullin4 Is Pro-Viral during West Nile Virus Infection of Culex Mosquitoes. *PLoS Pathog* **11**, e1005143 (2015).

80. X. Jia *et al.*, Label-free Proteomic Analysis of Exosomes Derived from Inducible Hepatitis B Virus-Replicating HepAD38 Cell Line. *Mol Cell Proteomics* **16**, S144-S160 (2017).

81. A. Karnowski *et al.*, B and T cells collaborate in antiviral responses via IL-6, IL-21, and transcriptional activator and coactivator, Oct2 and OBF-1. *J Exp Med* **209**, 2049-2064 (2012).

82. M. J. Rahman *et al.*, Restoration of the type I IFN-IL-1 balance through targeted blockade of PTGER4 inhibits autoimmunity in NOD mice. *JCI Insight* **3**, e97843 (2018).

83. G. Huang *et al.*, Profile of acute immune response in Chinese amphioxus upon Staphylococcus aureus and Vibrio parahaemolyticus infection. *Dev Comp Immunol* **31**, 1013-1023 (2007).

84. K. A. Staats *et al.*, Genetic ablation of IP3 receptor 2 increases cytokines and decreases survival of SOD1G93A mice. *Hum Mol Genet* **25**, 3491-3499 (2016).

85. D. Hotter *et al.*, IFI16 Targets the Transcription Factor Sp1 to Suppress HIV-1 Transcription and Latency Reactivation. *Cell Host Microbe* **25**, 858-872 e813 (2019).

86. P. Manivannan, V. Reddy, S. Mukherjee, K. N. Clark, K. Malathi, RNase L Induces Expression of A Novel Serine/Threonine Protein Kinase, DRAK1, to Promote Apoptosis. *Int J Mol Sci* **20**, 3535 (2019).

87. X. Zhang *et al.*, Epigenetically regulated miR-449a enhances hepatitis B virus replication by targeting cAMP-responsive element binding protein 5 and modulating hepatocytes phenotype. *Sci Rep* **6**, 25389 (2016).

88. C. H. Tsai *et al.*, CEACAM3 decreases asthma exacerbations and modulates respiratory syncytial virus latent infection in children. *Thorax* **75**, 725-734 (2020).

89. L. Seifert *et al.*, The Most N-Terminal Region of THSD7A Is the Predominant Target for Autoimmunity in THSD7A-Associated Membranous Nephropathy. *J Am Soc Nephrol* **29**, 1536-1548 (2018).

90. A. Boeck *et al.*, Ca(2+) and innate immune pathways are activated and differentially expressed in childhood asthma phenotypes. *Pediatr Allergy Immunol* **29**, 823-833 (2018).

91. E. C. Chan *et al.*, Regulator of G protein signaling 5 restricts neutrophil chemotaxis and trafficking. *J Biol Chem* **293**, 12690-12702 (2018).

92. F. Perugi *et al.*, Human Discs Large is a new negative regulator of human immunodeficiency virus-1 infectivity. *Mol Biol Cell* **20**, 498-508 (2009).

93. M. Lin *et al.*, USP38 Inhibits Type I Interferon Signaling by Editing TBK1 Ubiquitination through NLRP4 Signalosome. *Mol Cell* **64**, 267-281 (2016).

94. M. W. Woods *et al.*, Interferon-induced HERC5 is evolving under positive selection and inhibits HIV-1 particle production by a novel mechanism targeting Rev/RRE-dependent RNA nuclear export. *Retrovirology* **11**, 27 (2014).

95. Y. Liu *et al.*, Genetic variants in ERBB4 is associated with chronic hepatitis B virus infection. *Oncotarget* **7**, 4981-4992 (2016).

96. P. J. Hanson *et al.*, Cleavage and Sub-Cellular Redistribution of Nuclear Pore Protein 98 by Coxsackievirus B3 Protease 2A Impairs Cardioprotection. *Front Cell Infect Microbiol* **9**, 265 (2019).

97. H. Filippakis, D. A. Spandidos, G. Sourvinos, Herpesviruses: hijacking the Ras signaling pathway. *Biochim Biophys Acta* **1803**, 777-785 (2010).

98. P. J. McLaren *et al.*, Identification of potential HIV restriction factors by combining evolutionary genomic signatures with functional analyses. *Retrovirology* **12**, 41 (2015).

99. S. Liu, M. Dominska-Ngowe, D. M. Dykxhoorn, Target silencing of components of the conserved oligomeric Golgi complex impairs HIV-1 replication. *Virus Res* **192**, 92-102 (2014).

100. F. Facciotti *et al.*, Fine tuning by human CD1e of lipid-specific immune responses. *Proc Natl Acad Sci U S A* **108**, 14228-14233 (2011).

101. L. Kedzierski *et al.*, Suppressor of cytokine signaling 4 (SOCS4) protects against severe cytokine storm and enhances viral clearance during influenza infection. *PLoS Pathog* **10**, e1004134 (2014).

102. S. A. Gorski *et al.*, Expression of IL-5 receptor alpha by murine and human lung neutrophils. *PLoS One* **14**, e0221113 (2019).

103. T. Koga, A. Kawakami, The role of CaMK4 in immune responses. *Mod Rheumatol* **28**, 211-214 (2018).

104. M. H. Li *et al.*, Plasmacytoid Dendritic Cell Function and Cytokine Network Profiles in Patients with Acute or Chronic Hepatitis B Virus Infection. *Chin Med J (Engl)* **131**, 43-49 (2018).

105. Y. Kong *et al.*, VEGF-D: a novel biomarker for detection of COVID-19 progression. *Crit Care* **24**, 373 (2020).

106. K. L. Good, S. G. Tangye, Decreased expression of Kruppel-like factors in memory B cells induces the rapid response typical of secondary antibody responses. *Proc Natl Acad Sci U S A* **104**, 13420-13425 (2007).

107. A. Cui *et al.*, Dexamethasone-induced Kruppel-like factor 9 expression promotes hepatic gluconeogenesis and hyperglycemia. *J Clin Invest* **129**, 2266-2278 (2019).

108. M. Holzer *et al.*, Virus- and Interferon Alpha-Induced Transcriptomes of Cells from the Microbat Myotis daubentonii. *iScience* **19**, 647-661 (2019).

109. N. Abdelmagid *et al.*, The calcitonin receptor gene is a candidate for regulation of susceptibility to herpes simplex type 1 neuronal infection leading to encephalitis in rat. *PLoS Pathog* **8**, e1002753 (2012).

110. B. J. Williams *et al.*, The prototype gamma-2 herpesvirus nucleocytoplasmic shuttling protein, ORF 57, transports viral RNA through the cellular mRNA export pathway. *Biochem J* **387**, 295-308 (2005).

111. Y. Ji *et al.*, miR-155 harnesses Phf19 to potentiate cancer immunotherapy through epigenetic reprogramming of CD8(+) T cell fate. *Nat Commun* **10**, 2157 (2019).

112. J. Friesenhagen *et al.*, Highly pathogenic influenza viruses inhibit inflammatory response in monocytes via activation of rar-related orphan receptor RORalpha. *J Innate Immun* **5**, 505-518 (2013).

113. R. R. Dev *et al.*, Cytosine methylation by DNMT2 facilitates stability and survival of HIV-1 RNA in the host cell during infection. *Biochem J* **474**, 2009-2026 (2017).

114. L. A. Miles *et al.*, Anthrax toxin receptor 1 is the cellular receptor for Seneca Valley virus. *J Clin Invest* **127**, 2957-2967 (2017).

115. Q. Wang *et al.*, Condensin Smc4 promotes inflammatory innate immune response by epigenetically enhancing NEMO transcription. *J Autoimmun* **92**, 67-76 (2018).

116. N. Melnichuk, V. Kashuba, S. Rybalko, Z. Tkachuk, Complexes of Oligoribonucleotides with d-Mannitol Modulate the Innate Immune Response to Influenza A Virus H1N1 (A/FM/1/47) In Vivo. *Pharmaceuticals (Basel)* **11**, 73 (2018).

117. I. Korber *et al.*, Gene-Expression Profiling Suggests Impaired Signaling via the Interferon Pathway in Cstb-/- Microglia. *PLoS One* **11**, e0158195 (2016).

118. E. E. Kara *et al.*, Atypical chemokine receptor 4 shapes activated B cell fate. *J Exp Med* **215**, 801-813 (2018).

119. D. Marino *et al.*, APOBEC4 Enhances the Replication of HIV-1. *PLoS One* **11**, e0155422 (2016).

120. R. S. Harris, J. P. Dudley, APOBECs and virus restriction. *Virology* **479-480**, 131-145 (2015).

121. C. A. Jaeger-Ruckstuhl *et al.*, TNIK signaling imprints CD8(+) T cell memory formation early after priming. *Nat Commun* **11**, 1632 (2020).

122. W. M. Jiang, X. Y. Zhang, Y. Z. Zhang, L. Liu, H. Z. Lu, A high throughput RNAi screen reveals determinants of HIV-1 activity in host kinases. *Int J Clin Exp Pathol* **7**, 2229-2237 (2014).

123. L. Zhang *et al.*, Rab18 binds to classical swine fever virus NS5A and mediates viral replication and assembly in swine umbilical vein endothelial cells. *Virulence* **11**, 489-501 (2020).

124. P. Upla *et al.*, Calpain 1 and 2 are required for RNA replication of echovirus 1. *J Virol* **82**, 1581-1590 (2008).

125. J. Wu *et al.*, Exosomal MicroRNA-155 Inhibits Enterovirus A71 Infection by Targeting PICALM. *Int J Biol Sci* **15**, 2925-2935 (2019).

126. C. J. Carter, APP, APOE, complement receptor 1, clusterin and PICALM and their involvement in the herpes simplex life cycle. *Neurosci Lett* **483**, 96-100 (2010).

127. J. P. Bradfield *et al.*, A genome-wide meta-analysis of six type 1 diabetes cohorts identifies multiple associated loci. *PLoS Genet* **7**, e1002293 (2011).

128. G. Capalbo, T. Muller-Kuller, O. G. Ottmann, D. Hoelzer, U. J. Scheuring, HIV-1 infection suppresses expression of host cell cycle-associated gene PDS5A. *Intervirology* **55**, 263-275 (2012).

129. B. Han *et al.*, Long intergenic non-coding RNA GALMD3 in chicken Marek's disease. *Sci Rep* **7**, 10294 (2017).

130. S. Barik, Molecular Interactions between Pathogens and the Circadian Clock. *Int J Mol Sci* **20**, 5824 (2019).

131. M. M. E. Cruz, M. Miyazawa, D. Gozal, Putative contributions of circadian clock and sleep in the context of SARS-CoV-2 infection. *Eur Respir J* **55**, 2001023 (2020).

132. X. Jiang *et al.*, Proteomic Analysis of Zika Virus Infected Primary Human Fetal Neural Progenitors Suggests a Role for Doublecortin in the Pathological Consequences of Infection in the Cortex. *Front Microbiol* **9**, 1067 (2018).

133. E. Ish-Shalom *et al.*, Impaired SNX9 Expression in Immune Cells during Chronic Inflammation: Prognostic and Diagnostic Implications. *J Immunol* **196**, 156-167 (2016).

134. D. Long *et al.*, Identification of genetic variants associated with susceptibility to West Nile virus neuroinvasive disease. *Genes Immun* **17**, 298-304 (2016).

135. Q. Fu, B. Inankur, J. Yin, R. Striker, Q. Lan, Sterol Carrier Protein 2, a Critical Host Factor for Dengue Virus Infection, Alters the Cholesterol Distribution in Mosquito Aag2 Cells. *J Med Entomol* **52**, 1124-1134 (2015).

136. B. Vilagos *et al.*, Essential role of EBF1 in the generation and function of distinct mature B cell types. *J Exp Med* **209**, 775-792 (2012).

137. L. V. Glaser *et al.*, EBF1 binds to EBNA2 and promotes the assembly of EBNA2 chromatin complexes in B cells. *PLoS Pathog* **13**, e1006664 (2017).

138. E. Kip *et al.*, MALT1 Controls Attenuated Rabies Virus by Inducing Early Inflammation and T Cell Activation in the Brain. *J Virol* **92**, e02029-02017 (2018).

139. F. Victorino, S. Alper, Identifying novel spatiotemporal regulators of innate immunity. *Immunol Res* **55**, 3-9 (2013).

140. S. Bittner *et al.*, Endothelial TWIK-related potassium channel-1 (TREK1) regulates immune-cell trafficking into the CNS. *Nat Med* **19**, 1161-1165 (2013).

141. C. Schmidt *et al.*, Stretch-activated two-pore-domain (K2P) potassium channels in the heart: Focus on atrial fibrillation and heart failure. *Prog Biophys Mol Biol* **130**, 233-243 (2017).

142. S. Ruiz, E. Santos, X. R. Bustelo, RasGRF2, a guanosine nucleotide exchange factor for Ras GTPases, participates in T-cell signaling responses. *Mol Cell Biol* **27**, 8127-8142 (2007).

143. J. E. Fabre *et al.*, Decreased platelet aggregation, increased bleeding time and resistance to thromboembolism in P2Y1-deficient mice. *Nat Med* **5**, 1199-1202 (1999).

144. C. Feng, A. G. Mery, E. M. Beller, C. Favot, J. A. Boyce, Adenine nucleotides inhibit cytokine generation by human mast cells through a Gs-coupled receptor. *J Immunol* **173**, 7539-7547 (2004).

145. L. Ye *et al.*, USP49 negatively regulates cellular antiviral responses via deconjugating K63-linked ubiquitination of MITA. *PLoS Pathog* **15**, e1007680 (2019).

146. M. Soler Artigas *et al.*, Genome-wide association and large-scale follow up identifies 16 new loci influencing lung function. *Nat Genet* **43**, 1082-1090 (2011).

147. S. N. Waggoner, V. Kumar, Evolving role of 2B4/CD244 in T and NK cell responses during virus infection. *Front Immunol* **3**, 377 (2012).

148. H. Zhang *et al.*, Adenovirusmediated knockdown of activin A receptor type 2A attenuates immuneinduced hepatic fibrosis in mice and inhibits interleukin17induced activation of primary hepatic stellate cells. *Int J Mol Med* **42**, 279-289 (2018).

149. X. Y. Du *et al.*, The potential mechanism of INHBC and CSF1R in diabetic nephropathy. *Eur Rev Med Pharmacol Sci* **24**, 1970-1978 (2020).

150. A. Singhania *et al.*, Multitissue Transcriptomics Delineates the Diversity of Airway T Cell Functions in Asthma. *Am J Respir Cell Mol Biol* **58**, 261-270 (2018).

151. L. G. Gardinassi, A Cross-Study Biomarker Signature of Human Bronchial Epithelial Cells Infected with Respiratory Syncytial Virus. *Adv Virol* **2016**, 3605302 (2016).

152. F. Kiaee *et al.*, Clinical, Immunologic, and Molecular Spectrum of Patients with Immunodeficiency, Centromeric instability, and Facial anomalies (ICF) syndrome: a Systematic Review. *Endocr Metab Immune Disord Drug Targets* **20**, 000 (2020).

153. C. Chapat *et al.*, hCAF1/CNOT7 regulates interferon signalling by targeting STAT1. *EMBO J* **32**, 688-700 (2013).

154. C. Chiang *et al.*, The Human Papillomavirus E6 Oncoprotein Targets USP15 and TRIM25 To Suppress RIG-I-Mediated Innate Immune Signaling. *J Virol* **92**, e01737-01717 (2018).

155. A. X. Yang *et al.*, Molecular characterization of antigen-peptide pulsed dendritic cells: immature dendritic cells develop a distinct molecular profile when pulsed with antigen peptide. *PLoS One* **9**, e86306 (2014).

156. M. Niepmann, G. K. Gerresheim, Hepatitis C Virus Translation Regulation. *Int J Mol Sci* **21**, 2328 (2020).

157. S. Koks *et al.*, Psoriasis-Specific RNA Isoforms Identified by RNA-Seq Analysis of 173,446 Transcripts. *Front Med (Lausanne)* **3**, 46 (2016).

158. S. A. Wiltshire, G. A. Leiva-Torres, S. M. Vidal, Quantitative trait locus analysis, pathway analysis, and consomic mapping show genetic variants of Tnni3k, Fpgt, or H28 control susceptibility to viral myocarditis. *J Immunol* **186**, 6398-6405 (2011).

159. Z. Zhu *et al.*, Bioinformatics analysis on multiple Gene Expression Omnibus datasets of the hepatitis B virus infection and its response to the interferon-alpha therapy. *BMC Infect Dis* **20**, 84 (2020).

160. H. Dai *et al.*, Rictor deficiency in dendritic cells exacerbates acute kidney injury. *Kidney Int* **94**, 951-963 (2018).

161. S. K. Kuss-Duerkop *et al.*, Influenza virus differentially activates mTORC1 and mTORC2 signaling to maximize late stage replication. *PLoS Pathog* **13**, e1006635 (2017).

162. G. Zhou, T. Du, B. Roizman, The role of the CoREST/REST repressor complex in herpes simplex virus 1 productive infection and in latency. *Viruses* **5**, 1208-1218 (2013).

163. Y. Wu *et al.*, Selective autophagy controls the stability of transcription factor IRF3 to balance type I interferon production and immune suppression. *Autophagy*, 1-14 (2020).

164. F. Grey *et al.*, A viral microRNA down-regulates multiple cell cycle genes through mRNA 5'UTRs. *PLoS Pathog* **6**, e1000967 (2010).

165. D. Kanduc, Y. Shoenfeld, From HBV to HPV: Designing vaccines for extensive and intensive vaccination campaigns worldwide. *Autoimmun Rev* **15**, 1054-1061 (2016).

166. B. Khor *et al.*, The kinase DYRK1A reciprocally regulates the differentiation of Th17 and regulatory T cells. *Elife* **4**, e05920 (2015).

167. S. T. Hamilton *et al.*, Human cytomegalovirus utilises cellular dual-specificity tyrosine phosphorylation-regulated kinases during placental replication. *Placenta* **72-73**, 10-19 (2018).

168. C. Hutterer *et al.*, Inhibitors of dual-specificity tyrosine phosphorylation-regulated kinases (DYRK) exert a strong anti-herpesviral activity. *Antiviral Res* **143**, 113-121 (2017).

169. M. L. DeDiego, L. Martinez-Sobrido, D. J. Topham, Novel Functions of IFI44L as a Feedback Regulator of Host Antiviral Responses. *J Virol* **93**, e01159-01119 (2019).

170. J. A. Kerry, M. A. Priddy, T. L. Staley, T. R. Jones, R. M. Stenberg, The role of ATF in regulating the human cytomegalovirus DNA polymerase (UL54) promoter during viral infection. *J Virol* **71**, 2120-2126 (1997).

171. F. Licastro, E. Raschi, I. Carbone, E. Porcellini, Variants in Antiviral Genes are Risk Factors for Cognitive Decline and Dementia. *J Alzheimers Dis* **46**, 655-663 (2015).

172. S. J. Griffiths *et al.*, A systematic analysis of host factors reveals a Med23-interferon-lambda regulatory axis against herpes simplex virus type 1 replication. *PLoS Pathog* **9**, e1003514 (2013).

173. J. E. Shoemaker *et al.*, Integrated network analysis reveals a novel role for the cell cycle in 2009 pandemic influenza virus-induced inflammation in macaque lungs. *BMC Syst Biol* **6**, 117 (2012).

174. I. Liebscher *et al.*, Altered immune response in mice deficient for the G protein-coupled receptor GPR34. *J Biol Chem* **286**, 2101-2110 (2011).

175. P. Rawat, S. A. Spector, Development and characterization of a human microglia cell model of HIV-1 infection. *J Neurovirol* **23**, 33-46 (2017).

176. Y. Ma-Lauer *et al.*, p53 down-regulates SARS coronavirus replication and is targeted by the SARS-unique domain and PLpro via E3 ubiquitin ligase RCHY1. *Proc Natl Acad Sci U S A* **113**, E5192-5201 (2016).

177. T. P. Martin *et al.*, CaMKIIdelta interacts directly with IKKbeta and modulates NF-kappaB signalling in adult cardiac fibroblasts. *Cell Signal* **51**, 166-175 (2018).

178. W. W. T. Phua *et al.*, PPARbeta/delta Agonism Upregulates Forkhead Box A2 to Reduce Inflammation in C2C12 Myoblasts and in Skeletal Muscle. *Int J Mol Sci* **21**, 1747 (2020).

179. G. Qian *et al.*, NIRF, a Novel Ubiquitin Ligase, Inhibits Hepatitis B Virus Replication Through Effect on HBV Core Protein and H3 Histones. *DNA Cell Biol* **34**, 327-332 (2015).

180. C. Li *et al.*, The Immune Adaptor ADAP Regulates Reciprocal TGF-beta1-Integrin Crosstalk to Protect from Influenza Virus Infection. *PLoS Pathog* **11**, e1004824 (2015).

181. M. C. Horne *et al.*, Cyclin G2 is up-regulated during growth inhibition and B cell antigen receptor-mediated cell cycle arrest. *J Biol Chem* **272**, 12650-12661 (1997).

182. Y. Song *et al.*, E3 ligase FBXW7 is critical for RIG-I stabilization during antiviral responses. *Nat Commun* **8**, 14654 (2017).

183. N. Laguette *et al.*, Premature activation of the SLX4 complex by Vpr promotes G2/M arrest and escape from innate immune sensing. *Cell* **156**, 134-145 (2014).

184. L. Lundberg *et al.*, Venezuelan Equine Encephalitis Virus Capsid Implicated in Infection-Induced Cell Cycle Delay in vitro. *Front Microbiol* **9**, 3126 (2018).

185. Z. Zhu *et al.*, Zika Virus Targets Glioblastoma Stem Cells through a SOX2-Integrin alphavbeta5 Axis. *Cell Stem Cell* **26**, 187-204 e110 (2020).

186. R. Banos-Lara Mdel, B. Piao, A. Guerrero-Plata, Differential mucin expression by respiratory syncytial virus and human metapneumovirus infection in human epithelial cells. *Mediators Inflamm* **2015**, 347292 (2015).

187. X. Wang *et al.*, Diagnostic and prognostic value of mRNA expression of phospholipase C beta family genes in hepatitis B virusassociated hepatocellular carcinoma. *Oncol Rep* **41**, 2855-2875 (2019).

188. M. S. Shiels *et al.*, HIV Infection and Circulating Levels of Prosurfactant Protein B and Surfactant Protein D. *J Infect Dis* **217**, 413-417 (2018).

189. E. Ryu *et al.*, Cordycepin is a novel chemical suppressor of Epstein-Barr virus replication. *Oncoscience* **1**, 866-881 (2014).

190. U. Dixit *et al.*, FUSE Binding Protein 1 Facilitates Persistent Hepatitis C Virus Replication in Hepatoma Cells by Regulating Tumor Suppressor p53. *J Virol* **89**, 7905-7921 (2015).

191. T. Tzanavari *et al.*, Metformin protects against infection-induced myocardial dysfunction. *Metabolism* **65**, 1447-1458 (2016).

192. H. Huang *et al.*, Hepatitis C virus inhibits AKT-tuberous sclerosis complex (TSC), the mechanistic target of rapamycin (MTOR) pathway, through endoplasmic reticulum stress to induce autophagy. *Autophagy* **9**, 175-195 (2013).

193. A. Kapoor, M. Forman, R. Arav-Boger, Activation of nucleotide oligomerization domain 2 (NOD2) by human cytomegalovirus initiates innate immune responses and restricts virus replication. *PLoS One* **9**, e92704 (2014).

194. N. Drayman *et al.*, Dynamic Proteomics of Herpes Simplex Virus Infection. *mBio* **8**, e01612-01617 (2017).

195. X. Zhou, W. Y. Li, H. Y. Wang, The roles and mechanisms of MST1/2 in the innate immune response. *Yi Chuan* **39**, 642-649 (2017).

196. C. Atkins *et al.*, Global Human-Kinase Screening Identifies Therapeutic Host Targets against Influenza. *J Biomol Screen* **19**, 936-946 (2014).

197. W. Chen *et al.*, Human Papillomavirus 16 E6 Induces FoxM1B in Oral Keratinocytes through GRHL2. *J Dent Res* **97**, 795-802 (2018).

198. Y. Ji *et al.*, Serum from Chronic Hepatitis B Patients Promotes Growth and Proliferation via the IGF-II/IGF-IR/MEK/ERK Signaling Pathway in Hepatocellular Carcinoma Cells. *Cell Physiol Biochem* **47**, 39-53 (2018).

199. W. S. Polachek *et al.*, High-Throughput Small Interfering RNA Screening Identifies Phosphatidylinositol 3-Kinase Class II Alpha as Important for Production of Human Cytomegalovirus Virions. *J Virol* **90**, 8360-8371 (2016).

200. D. B. Hancock *et al.*, Meta-analyses of genome-wide association studies identify multiple loci associated with pulmonary function. *Nat Genet* **42**, 45-52 (2010).

201. A. Ranjan, A. Singh, G. K. Walia, M. P. Sachdeva, V. Gupta, Genetic underpinnings of lung function and COPD. *J Genet* **98**, 76 (2019).

202. E. Morita *et al.*, Identification of human MVB12 proteins as ESCRT-I subunits that function in HIV budding. *Cell Host Microbe* **2**, 41-53 (2007).

203. F. Salerno *et al.*, Translational repression of pre-formed cytokine-encoding mRNA prevents chronic activation of memory T cells. *Nat Immunol* **19**, 828-837 (2018).

204. C. E. Hamblet, S. L. Makowski, J. M. Tritapoe, J. L. Pomerantz, NK Cell Maturation and Cytotoxicity Are Controlled by the Intramembrane Aspartyl Protease SPPL3. *J Immunol* **196**, 2614-2626 (2016).

205. D. Lan, C. Tang, M. Li, H. Yue, Screening and identification of differentially expressed genes from chickens infected with Newcastle disease virus by suppression subtractive hybridization. *Avian Pathol* **39**, 151-159 (2010).

206. N. Kato *et al.*, Large-scale search of single nucleotide polymorphisms for hepatocellular carcinoma susceptibility genes in patients with hepatitis C. *Hepatology* **42**, 846-853 (2005).

207. M. Doi *et al.*, Gpr176 is a Gz-linked orphan G-protein-coupled receptor that sets the pace of circadian behaviour. *Nat Commun* **7**, 10583 (2016).

208. G. Li *et al.*, Insulin-Like Growth Factor 1 Regulates Acute Inflammatory Lung Injury Mediated by Influenza Virus Infection. *Front Microbiol* **10**, 2541 (2019).

209. W. Zhu *et al.*, Interleukin receptor activates a MYD88-ARNO-ARF6 cascade to disrupt vascular stability. *Nature* **492**, 252-255 (2012).

210. A. Fernandez-Gamba, M. C. Leal, L. Morelli, E. M. Castano, Insulin-degrading enzyme: structure-function relationship and its possible roles in health and disease. *Curr Pharm Des* **15**, 3644-3655 (2009).

211. Q. Li *et al.*, Insulin degrading enzyme induces a conformational change in varicella-zoster virus gE, and enhances virus infectivity and stability. *PLoS One* **5**, e11327 (2010).

212. C. Wu, G. Xu, S. A. Tsai, W. J. Freed, C. T. Lee, Transcriptional profiles of type 2 diabetes in human skeletal muscle reveal insulin resistance, metabolic defects, apoptosis, and molecular signatures of immune activation in response to infections. *Biochem Biophys Res Commun* **482**, 282-288 (2017).

213. H. Wang *et al.*, STAT3 Regulates the Type I IFN-Mediated Antiviral Response by Interfering with the Nuclear Entry of STAT1. *Int J Mol Sci* **20**, 4870 (2019).

214. J. T. Crowl, D. B. Stetson, SUMO2 and SUMO3 redundantly prevent a noncanonical type I interferon response. *Proc Natl Acad Sci U S A* **115**, 6798-6803 (2018).

215. A. Eyking, F. Ferber, S. Kohler, H. Reis, E. Cario, TRIM58 Restrains Intestinal Mucosal Inflammation by Negatively Regulating TLR2 in Myeloid Cells. *J Immunol* **203**, 1636-1649 (2019).

216. Y. Y. Lin *et al.*, Genome-wide association analysis identifies a GLUL haplotype for familial hepatitis B virus-related hepatocellular carcinoma. *Cancer* **123**, 3966-3976 (2017).

217. A. S. Gallouet *et al.*, Macrophage production and activation are dependent on TRIM33. *Oncotarget* **8**, 5111-5122 (2017).

218. S. P. Zhang *et al.*, FOXC1 up-regulates the expression of toll-like receptors in myocardial ischaemia. *J Cell Mol Med* **23**, 7566-7580 (2019).

219. Z. Xiang, S. Wang, Y. Xiang, Up-regulated microRNA499a by hepatitis B virus induced hepatocellular carcinogenesis via targeting MAPK6. *PLoS One* **9**, e111410 (2014).

220. M. Marquis *et al.*, The non-classical MAP kinase ERK3 controls T cell activation. *PLoS One* **9**, e86681 (2014).

221. E. R. Tammineni *et al.*, The beta4 subunit of Cav1.2 channels is required for an optimal interferon response in cardiac muscle cells. *Sci Signal* **11**, eaaj1676 (2018).

222. S. Seddighi *et al.*, SPARCL1 Accelerates Symptom Onset in Alzheimer's Disease and Influences Brain Structure and Function During Aging. *J Alzheimers Dis* **61**, 401-414 (2018).

223. V. Dhingra, X. Li, Y. Liu, Z. F. Fu, Proteomic profiling reveals that rabies virus infection results in differential expression of host proteins involved in ion homeostasis and synaptic physiology in the central nervous system. *J Neurovirol* **13**, 107-117 (2007).

224. Y. Qin *et al.*, TRIM9 short isoform preferentially promotes DNA and RNA virus-induced production of type I interferon by recruiting GSK3beta to TBK1. *Cell Res* **26**, 613-628 (2016).

225. M. Yuan *et al.*, ABCC4, ABCC5 and SLC28A1 polymorphisms: host genome on responses of chronic hepatitis B patients with entecavir treatment. *Antivir Ther* **21**, 689-696 (2016).

226. F. Potus, C. C. T. Hindmarch, K. J. Dunham-Snary, J. Stafford, S. L. Archer, Transcriptomic Signature of Right Ventricular Failure in Experimental Pulmonary Arterial Hypertension: Deep Sequencing Demonstrates Mitochondrial, Fibrotic, Inflammatory and Angiogenic Abnormalities. *Int J Mol Sci* **19**, 2730 (2018).

227. M. Wessling-Resnick, Nramp1 and Other Transporters Involved in Metal Withholding during Infection. *J Biol Chem* **290**, 18984-18990 (2015).

228. C. M. Karch, A. M. Goate, Alzheimer's disease risk genes and mechanisms of disease pathogenesis. *Biol Psychiatry* **77**, 43-51 (2015).

229. J. J. Chmielewska, B. Kuzniewska, J. Milek, K. Urbanska, M. Dziembowska, Neuroligin 1, 2, and 3 Regulation at the Synapse: FMRP-Dependent Translation and Activity-Induced Proteolytic Cleavage. *Mol Neurobiol* **56**, 2741-2759 (2019).

230. L. Stertz, V. Contreras-Shannon, N. Monroy-Jaramillo, J. Sun, C. Walss-Bass, BACE1-Deficient Mice Exhibit Alterations in Immune System Pathways. *Mol Neurobiol* **55**, 709-717 (2018).

231. S. Sil *et al.*, HIV-1 Tat-mediated astrocytic amyloidosis involves the HIF-1alpha/lncRNA BACE1-AS axis. *PLoS Biol* **18**, e3000660 (2020).

232. B. Readhead *et al.*, Multiscale Analysis of Independent Alzheimer's Cohorts Finds Disruption of Molecular, Genetic, and Clinical Networks by Human Herpesvirus. *Neuron* **99**, 64-82 e67 (2018).

233. I. Muylaert, P. Elias, Contributions of nucleotide excision repair, DNA polymerase eta, and homologous recombination to replication of UV-irradiated herpes simplex virus type 1. *J Biol Chem* **285**, 13761-13768 (2010).

234. P. Wolter *et al.*, GAS2L3, a target gene of the DREAM complex, is required for proper cytokinesis and genomic stability. *J Cell Sci* **125**, 2393-2406 (2012).

235. Y. Sharaby *et al.*, Gas2l3 is essential for brain morphogenesis and development. *Dev Biol* **394**, 305-313 (2014).

236. S. Stopp *et al.*, Deletion of Gas2l3 in mice leads to specific defects in cardiomyocyte cytokinesis during development. *Proc Natl Acad Sci U S A* **114**, 8029-8034 (2017).

237. M. Panos, G. P. Christophi, M. Rodriguez, I. A. Scarisbrick, Differential expression of multiple kallikreins in a viral model of multiple sclerosis points to unique roles in the innate and adaptive immune response. *Biol Chem* **395**, 1063-1073 (2014).

238. M. Tan *et al.*, Fatty Acid Metabolism in Immune Cells: A Bioinformatics Analysis of Genes Involved in Ulcerative Colitis. *DNA Cell Biol* **39**, 1573-1582 (2020).

239. D. L. Golej *et al.*, Long-chain acyl-CoA synthetase 4 modulates prostaglandin E(2) release from human arterial smooth muscle cells. *J Lipid Res* **52**, 782-793 (2011).

240. W. Shen *et al.*, Tomosyn regulates the small RhoA GTPase to control the dendritic stability of neurons and the surface expression of AMPA receptors. *J Neurosci Res* **98**, 1213-1231 (2020).

241. L. L. Zuo *et al.*, Cadherin 6 is activated by Epstein-Barr virus LMP1 to mediate EMT and metastasis as an interplay node of multiple pathways in nasopharyngeal carcinoma. *Oncogenesis* **6**, 402 (2017).

242. P. Rao, P. K. Suvas, A. D. Jerome, J. J. Steinle, S. Suvas, Role of Insulin-Like Growth Factor Binding Protein-3 in the Pathogenesis of Herpes Stromal Keratitis. *Invest Ophthalmol Vis Sci* **61**, 46 (2020).

243. J. Yan, M. C. Shun, Y. Zhang, C. Hao, J. Skowronski, HIV-1 Vpr counteracts HLTF-mediated restriction of HIV-1 infection in T cells. *Proc Natl Acad Sci U S A* **116**, 9568-9577 (2019).

244. C. C. Sheu *et al.*, Bioinformatic analysis of nextgeneration sequencing data to identify dysregulated genes in fibroblasts of idiopathic pulmonary fibrosis. *Int J Mol Med* **43**, 1643-1656 (2019).

245. F. Palmieri, Mitochondrial transporters of the SLC25 family and associated diseases: a review. *J Inherit Metab Dis* **37**, 565-575 (2014).

246. H. Chen *et al.*, Role of the integrin-linked kinase/PINCH1/alpha-parvin complex in cardiac myocyte hypertrophy. *Lab Invest* **85**, 1342-1356 (2005).

247. N. Zhang *et al.*, Leucine-rich repeat-containing G protein-coupled receptor 4 facilitates vesicular stomatitis virus infection by binding vesicular stomatitis virus glycoprotein. *J Biol Chem* **292**, 16527-16538 (2017).

248. C. K. Huang *et al.*, Lgr4 Governs a Pro-Inflammatory Program in Macrophages to Antagonize Post-Infarction Cardiac Repair. *Circ Res* **127**, 953-973 (2020).

249. H. L. Liu *et al.*, Gene signatures of SARS-CoV/SARS-CoV-2-infected ferret lungs in short- and long-term models. *Infect Genet Evol* **85**, 104438 (2020).

250. S. Kipper *et al.*, New host factors important for respiratory syncytial virus (RSV) replication revealed by a novel microfluidics screen for interactors of matrix (M) protein. *Mol Cell Proteomics* **14**, 532-543 (2015).

251. S. B. Drysdale *et al.*, Functional and genetic predisposition to rhinovirus lower respiratory tract infections in prematurely born infants. *Eur J Pediatr* **175**, 1943-1949 (2016).

252. A. S. Yeo *et al.*, Lack of clinical manifestations in asymptomatic dengue infection is attributed to broad down-regulation and selective up-regulation of host defence response genes. *PLoS One* **9**, e92240 (2014).

253. A. Guilmatre, G. Huguet, R. Delorme, T. Bourgeron, The emerging role of SHANK genes in neuropsychiatric disorders. *Dev Neurobiol* **74**, 113-122 (2014).

254. F. Guan *et al.*, Evaluation of the relationships of the WBP1L gene with schizophrenia and the general psychopathology scale based on a case-control study. *Am J Med Genet B Neuropsychiatr Genet* **183**, 164-171 (2020).

255. S. Biswas *et al.*, Differentially expressed host long intergenic noncoding RNA and mRNA in HIV-1 and HIV-2 infection. *Sci Rep* **8**, 2546 (2018).

256. T. Kelesidis, J. S. Currier, O. O. Yang, T. T. Brown, Role of RANKL-RANK/osteoprotegerin pathway in cardiovascular and bone disease associated with HIV infection. *AIDS Rev* **16**, 123-133 (2014).

257. D. Zheng *et al.*, Long noncoding RNA Crnde attenuates cardiac fibrosis via Smad3-Crnde negative feedback in diabetic cardiomyopathy. *FEBS J* **286**, 1645-1655 (2019).

258. A. A. Ajibade, H. Y. Wang, R. F. Wang, Cell type-specific function of TAK1 in innate immune signaling. *Trends Immunol* **34**, 307-316 (2013).

259. K. Rajasekaran *et al.*, Signaling by Fyn-ADAP via the Carma1-Bcl-10-MAP3K7 signalosome exclusively regulates inflammatory cytokine production in NK cells. *Nat Immunol* **14**, 1127-1136 (2013).

260. M. G. Grutter, J. Luban, TRIM5 structure, HIV-1 capsid recognition, and innate immune signaling. *Curr Opin Virol* **2**, 142-150 (2012).

261. L. Li *et al.*, HHEX: A Crosstalker between HCMV Infection and Proliferation of VSMCs. *Front Cell Infect Microbiol* **6**, 169 (2016).

262. Y. A. Kung, C. T. Hung, K. Y. Chien, S. R. Shih, Control of the negative IRES trans-acting factor KHSRP by ubiquitination. *Nucleic Acids Res* **45**, 271-287 (2017).

263. C. Shen *et al.*, mTOR- and SGK-Mediated Connexin 43 Expression Participates in Lipopolysaccharide-Stimulated Macrophage Migration through the iNOS/Src/FAK Axis. *J Immunol* **201**, 2986-2997 (2018).

264. M. Fang *et al.*, Transfection of Sox11 plasmid alleviates ventilator-induced lung injury via Sox11 and FAK. *Biochem Biophys Res Commun* **512**, 182-188 (2019).

265. H. Elbahesh, S. Bergmann, C. J. Russell, Focal adhesion kinase (FAK) regulates polymerase activity of multiple influenza A virus subtypes. *Virology* **499**, 369-374 (2016).

266. P. I. Chi *et al.*, Avian reovirus sigmaA-modulated suppression of lactate dehydrogenase and upregulation of glutaminolysis and the mTOC1/eIF4E/HIF-1alpha pathway to enhance glycolysis and the TCA cycle for virus replication. *Cell Microbiol* **20**, e12946 (2018).

267. A. Wallrapp *et al.*, The neuropeptide NMU amplifies ILC2-driven allergic lung inflammation. *Nature* **549**, 351-356 (2017).

268. V. Cardoso *et al.*, Neuronal regulation of type 2 innate lymphoid cells via neuromedin U. *Nature* **549**, 277-281 (2017).

269. N. F. Gonzalez-Cadavid *et al.*, Organization of the human myostatin gene and expression in healthy men and HIV-infected men with muscle wasting. *Proc Natl Acad Sci U S A* **95**, 14938-14943 (1998).

270. D. Wang *et al.*, Lower Circulating Folate Induced by a Fidgetin Intronic Variant Is Associated With Reduced Congenital Heart Disease Susceptibility. *Circulation* **135**, 1733-1748 (2017).

271. M. Abdel-Nour *et al.*, The heme-regulated inhibitor is a cytosolic sensor of protein misfolding that controls innate immune signaling. *Science* **365**, eaaw4144 (2019).

272. A. A. Elfiky, I. M. Ibrahim, Zika virus envelope - heat shock protein A5 (GRP78) binding site prediction. *J Biomol Struct Dyn*, 1-13 (2020).

273. Y. Guo *et al.*, PHLDA1 is a new therapeutic target of oxidative stress and ischemia reperfusion-induced myocardial injury. *Life Sci* **245**, 117347 (2020).

274. C. Han *et al.*, PHLDA1 promotes microglia-mediated neuroinflammation via regulating K63-linked ubiquitination of TRAF6. *Brain Behav Immun* **88**, 640-653 (2020).

275. M. Kainulainen, S. Lau, C. E. Samuel, V. Hornung, F. Weber, NSs Virulence Factor of Rift Valley Fever Virus Engages the F-Box Proteins FBXW11 and beta-TRCP1 To Degrade the Antiviral Protein Kinase PKR. *J Virol* **90**, 6140-6147 (2016).

276. S. Yuan *et al.*, Plasma Phospholipid Fatty Acids, FADS1 and Risk of 15 Cardiovascular Diseases: A Mendelian Randomisation Study. *Nutrients* **11**, 3001 (2019).

277. B. Lucas, J. Hardin, Mind the (sr)GAP - roles of Slit-Robo GAPs in neurons, brains and beyond. *J Cell Sci* **130**, 3965-3974 (2017).

278. D. Xu, L. Lin, X. Lin, Z. Huang, Z. Lei, Immunoregulation of autocrine prolactin: suppressing the expression of costimulatory molecules and cytokines in T lymphocytes by prolactin receptor knockdown. *Cell Immunol* **263**, 71-78 (2010).

279. E. Rouka, C. Hatzoglou, K. I. Gourgoulianis, S. G. Zarogiannis, Interactome networks between the human respiratory syncytial virus (HRSV), the human metapneumovirus (EtaMPV), and their host: In silico investigation and comparative functional enrichment analysis. *Microb Pathog* **141**, 104000 (2020).

280. K. L. O'Hagan, S. D. Miller, H. Phee, Pak2 is essential for the function of Foxp3+ regulatory T cells through maintaining a suppressive Treg phenotype. *Sci Rep* **7**, 17097 (2017).

281. P. Binder *et al.*, Pak2 as a Novel Therapeutic Target for Cardioprotective Endoplasmic Reticulum Stress Response. *Circ Res* **124**, 696-711 (2019).

282. W. Xie, S. Jin, J. Cui, The NEDD4-USP13 axis facilitates autophagy via deubiquitinating PIK3C3. *Autophagy* **16**, 1150-1151 (2020).

283. B. Biterge Sut, Molecular profiling of immune cell-enriched Severe Acute Respiratory Syndrome Coronavirus 2 (SARS-CoV-2) interacting protein USP13. *Life Sci* **258**, 118170 (2020).

284. B. Pang, C. Hu, G. Wu, Y. Zhang, G. Lin, Identification of Target Genes in Hypertension and Left Ventricular Remodeling. *Medicine (Baltimore)* **99**, e21195 (2020).

285. M. Hagmann, O. Georgiev, W. Schaffner, P. Douville, Transcription factors interacting with herpes simplex virus alpha gene promoters in sensory neurons. *Nucleic Acids Res* **23**, 4978-4985 (1995).

286. Y. Fan *et al.*, Temporal profile of the renal transcriptome of HIV-1 transgenic mice during disease progression. *PLoS One* **9**, e93019 (2014).

287. D. A. Ornelles, L. R. Gooding, M. L. Dickherber, M. Policard, C. Garnett-Benson, Limited but durable changes to cellular gene expression in a model of latent adenovirus infection are reflected in childhood leukemic cell lines. *Virology* **494**, 67-77 (2016).

288. J. E. Alford, M. Marongiu, G. L. Watkins, E. C. Anderson, Human Immunodeficiency Virus Type 2 (HIV-2) Gag Is Trafficked in an AP-3 and AP-5 Dependent Manner. *PLoS One* **11**, e0158941 (2016).

289. G. He *et al.*, Role of CLIC4 in the host innate responses to bacterial lipopolysaccharide. *Eur J Immunol* **41**, 1221-1230 (2011).

290. M. Muller *et al.*, Chikungunya virus requires cellular chloride channels for efficient genome replication. *PLoS Negl Trop Dis* **13**, e0007703 (2019).

291. G. Stakaityte *et al.*, The cellular chloride channels CLIC1 and CLIC4 contribute to virus-mediated cell motility. *J Biol Chem* **293**, 4582-4590 (2018).

292. T. Liuyu *et al.*, Induction of OTUD4 by viral infection promotes antiviral responses through deubiquitinating and stabilizing MAVS. *Cell Res* **29**, 67-79 (2019).

293. G. Malik, Y. Zhou, Innate Immune Sensing of Influenza A Virus. *Viruses* **12**, (2020).

294. D. F. van den Berg, A. A. Te Velde, Severe COVID-19: NLRP3 Inflammasome Dysregulated. *Front Immunol* **11**, 1580 (2020).

295. A. Paniri, H. Akhavan-Niaki, Emerging role of IL-6 and NLRP3 inflammasome as potential therapeutic targets to combat COVID-19: Role of lncRNAs in cytokine storm modulation. *Life Sci* **257**, 118114 (2020).

296. Z. Su *et al.*, ABIN-1 heterozygosity sensitizes to innate immune response in both RIPK1-dependent and RIPK1-independent manner. *Cell Death Differ* **26**, 1077-1088 (2019).

297. C. Fan *et al.*, Up-regulation of A20/ABIN1 contributes to inefficient M1 macrophage polarization during Hepatitis C virus infection. *Virol J* **12**, 147 (2015).

298. L. Gao *et al.*, ABIN1 protein cooperates with TAX1BP1 and A20 proteins to inhibit antiviral signaling. *J Biol Chem* **286**, 36592-36602 (2011).

299. R. Cozzolongo *et al.*, Serotonin gene polymorphisms and lifetime mood disorders in predicting interferon-induced depression in chronic hepatitis C. *J Affect Disord* **183**, 90-97 (2015).

300. D. Kim *et al.*, Suppression of Hepatitis C Virus Genome Replication and Particle Production by a Novel Diacylglycerol Acyltransferases Inhibitor. *Molecules* **23**, 2083 (2018).

301. J. M. Fletcher *et al.*, Congenic analysis of the NKT cell control gene Nkt2 implicates the peroxisomal protein Pxmp4. *J Immunol* **181**, 3400-3412 (2008).

302. D. Dewi Pamungkas Putri *et al.*, PtdIns3P phosphatases MTMR3 and MTMR4 negatively regulate innate immune responses to DNA through modulating STING trafficking. *J Biol Chem* **294**, 8412-8423 (2019).

303. L. Yang *et al.*, Diagnostic and prognostic values of the mRNA expression of excision repair cross-complementation enzymes in hepatitis B virus-related hepatocellular carcinoma. *Cancer Manag Res* **10**, 5313-5328 (2018).

304. L. P. Hayden *et al.*, Childhood asthma is associated with COPD and known asthma variants in COPDGene: a genome-wide association study. *Respir Res* **19**, 209 (2018).

305. Y. Li, J. Dai, M. Song, P. Fitzgerald-Bocarsly, M. Kiledjian, Dcp2 decapping protein modulates mRNA stability of the critical interferon regulatory factor (IRF) IRF-7. *Mol Cell Biol* **32**, 1164-1172 (2012).

306. J. C. Reed *et al.*, Formation of RNA Granule-Derived Capsid Assembly Intermediates Appears To Be Conserved between Human Immunodeficiency Virus Type 1 and the Nonprimate Lentivirus Feline Immunodeficiency Virus. *J Virol* **92**, e01761-01717 (2018).

307. E. D. Morrell *et al.*, Genetic Variation in MAP3K1 Associates with Ventilator-Free Days in Acute Respiratory Distress Syndrome. *Am J Respir Cell Mol Biol* **58**, 117-125 (2018).

308. R. Yoshida *et al.*, TRAF6 and MEKK1 play a pivotal role in the RIG-I-like helicase antiviral pathway. *J Biol Chem* **283**, 36211-36220 (2008).

309. Y. Guo *et al.*, During Aspergillus Infection, Monocyte-Derived DCs, Neutrophils, and Plasmacytoid DCs Enhance Innate Immune Defense through CXCR3-Dependent Crosstalk. *Cell Host Microbe* **28**, 104-116 e104 (2020).

310. W. G. Glass, K. Subbarao, B. Murphy, P. M. Murphy, Mechanisms of host defense following severe acute respiratory syndrome-coronavirus (SARS-CoV) pulmonary infection of mice. *J Immunol* **173**, 4030-4039 (2004).

311. P. Conti, A. Younes, Coronavirus COV-19/SARS-CoV-2 affects women less than men: clinical response to viral infection. *J Biol Regul Homeost Agents* **34**, 339-343 (2020).

312. A. Slonchak, R. P. Shannon, G. Pali, A. A. Khromykh, Human MicroRNA miR-532-5p Exhibits Antiviral Activity against West Nile Virus via Suppression of Host Genes SESTD1 and TAB3 Required for Virus Replication. *J Virol* **90**, 2388-2402 (2015).

313. S. B. Van Engelenburg *et al.*, Distribution of ESCRT machinery at HIV assembly sites reveals virus scaffolding of ESCRT subunits. *Science* **343**, 653-656 (2014).

314. P. Resa-Infante *et al.*, Importin-alpha7 is required for enhanced influenza A virus replication in the alveolar epithelium and severe lung damage in mice. *J Virol* **88**, 8166-8179 (2014).

315. T. M. Schwarz *et al.*, VP24-Karyopherin Alpha Binding Affinities Differ between Ebolavirus Species, Influencing Interferon Inhibition and VP24 Stability. *J Virol* **91**, e01715-01716 (2017).

316. L. Yang *et al.*, Karyopherin Alpha 6 Is Required for Replication of Porcine Reproductive and Respiratory Syndrome Virus and Zika Virus. *J Virol* **92**, e00072-00018 (2018).

317. S. Han *et al.*, Sec62 Suppresses Foot-and-Mouth Disease Virus Proliferation by Promotion of IRE1alpha-RIG-I Antiviral Signaling. *J Immunol* **203**, 429-440 (2019).

318. D. Saadoun *et al.*, Role of matrix metalloproteinases, proinflammatory cytokines, and oxidative stress-derived molecules in hepatitis C virus-associated mixed cryoglobulinemia vasculitis neuropathy. *Arthritis Rheum* **56**, 1315-1324 (2007).

319. G. Provenzano *et al.*, Comparative Gene Expression Analysis of Two Mouse Models of Autism: Transcriptome Profiling of the BTBR and En2 (-/-) Hippocampus. *Front Neurosci* **10**, 396 (2016).

320. N. Punia, M. Primon, G. R. Simpson, H. S. Pandha, R. Morgan, Membrane insertion and secretion of the Engrailed-2 (EN2) transcription factor by prostate cancer cells may induce antiviral activity in the stroma. *Sci Rep* **9**, 5138 (2019).

321. Y. Jiang *et al.*, Repression of Hox genes by LMP1 in nasopharyngeal carcinoma and modulation of glycolytic pathway genes by HoxC8. *Oncogene* **34**, 6079-6091 (2015).

322. I. Perez de Castro *et al.*, Mice deficient for N-ras: impaired antiviral immune response and T-cell function. *Cancer Res* **63**, 1615-1622 (2003).

323. T. Saxena *et al.*, Combined miRNA and mRNA signature identifies key molecular players and pathways involved in chikungunya virus infection in human cells. *PLoS One* **8**, e79886 (2013).

324. G. Osmak, N. Baulina, P. Koshkin, O. Favorova, Collapsing the list of myocardial infarction-related differentially expressed genes into a diagnostic signature. *J Transl Med* **18**, 231 (2020).

325. H. Krenzlin *et al.*, Cytomegalovirus promotes murine glioblastoma growth via pericyte recruitment and angiogenesis. *J Clin Invest* **129**, 1671-1683 (2019).

326. W. H. Lee *et al.*, Deduction of novel genes potentially involved in hypoxic AC16 human cardiomyocytes using next-generation sequencing and bioinformatics approaches. *Int J Mol Med* **42**, 2489-2502 (2018).

327. T. Feng *et al.*, Loss of TMEM106B and PGRN leads to severe lysosomal abnormalities and neurodegeneration in mice. *EMBO Rep* **21**, e50219 (2020).

328. W. Wang *et al.*, Zika virus infection induces host inflammatory responses by facilitating NLRP3 inflammasome assembly and interleukin-1beta secretion. *Nat Commun* **9**, 106 (2018).

329. R. Ren *et al.*, The H7N9 influenza A virus infection results in lethal inflammation in the mammalian host via the NLRP3-caspase-1 inflammasome. *Sci Rep* **7**, 7625 (2017).

330. L. A. de Castro-Jorge *et al.*, The NLRP3 inflammasome is involved with the pathogenesis of Mayaro virus. *PLoS Pathog* **15**, e1007934 (2019).

331. A. H. de Wilde *et al.*, A Kinome-Wide Small Interfering RNA Screen Identifies Proviral and Antiviral Host Factors in Severe Acute Respiratory Syndrome Coronavirus Replication, Including Double-Stranded RNA-Activated Protein Kinase and Early Secretory Pathway Proteins. *J Virol* **89**, 8318-8333 (2015).

332. B. E. Young *et al.*, Viral dynamics and immune correlates of COVID-19 disease severity. *Clin Infect Dis*, ciaa1280 (2020).

333. M. Yamada *et al.*, Signal-transducing adaptor molecules STAM1 and STAM2 are required for T-cell development and survival. *Mol Cell Biol* **22**, 8648-8658 (2002).

334. K. Pindolia, H. Li, C. Cardwell, B. Wolf, Characterization and functional analysis of cellular immunity in mice with biotinidase deficiency. *Mol Genet Metab* **112**, 49-56 (2014).

335. S. Agrawal, A. Agrawal, H. M. Said, Biotin deficiency enhances the inflammatory response of human dendritic cells. *Am J Physiol Cell Physiol* **311**, C386-391 (2016).

336. M. Nagao *et al.*, Coronary Disease-Associated Gene TCF21 Inhibits Smooth Muscle Cell Differentiation by Blocking the Myocardin-Serum Response Factor Pathway. *Circ Res* **126**, 517-529 (2020).

337. Q. Cheng *et al.*, Genetic Profiles Related to Pathogenesis in Sporadic Intracranial Aneurysm Patients. *World Neurosurg* **131**, e23-e31 (2019).

338. A. Ramos-Nascimento *et al.*, KIF13A mediates trafficking of influenza A virus ribonucleoproteins. *J Cell Sci* **130**, 4038-4050 (2017).

339. S. K. Fehling *et al.*, The microtubule motor protein KIF13A is involved in intracellular trafficking of the Lassa virus matrix protein Z. *Cell Microbiol* **15**, 315-334 (2013).

340. J. Sanchez-Ruiz, R. Mejias, M. Garcia-Belando, D. F. Barber, A. Gonzalez-Garcia, Ral GTPases regulate cell-mediated cytotoxicity in NK cells. *J Immunol* **187**, 2433-2441 (2011).

341. F. Zimprich, J. Winter, H. Wege, H. Lassmann, Coronavirus induced primary demyelination: indications for the involvement of a humoral immune response. *Neuropathol Appl Neurobiol* **17**, 469-484 (1991).

342. Y. C. Kwon, R. Ray, Complement Regulation and Immune Evasion by Hepatitis C Virus. *Methods Mol Biol* **1911**, 337-347 (2019).

343. M. Barathan *et al.*, Chronic hepatitis C virus infection triggers spontaneous differential expression of biosignatures associated with T cell exhaustion and apoptosis signaling in peripheral blood mononucleocytes. *Apoptosis* **20**, 466-480 (2015).

344. Y. Guo *et al.*, Single-nucleotide polymorphisms in the TSPYL-4 and NT5DC1 genes are associated with susceptibility to chronic obstructive pulmonary disease. *Mol Med Rep* **6**, 631-638 (2012).

345. S. S. Hwang, L. K. Kim, G. R. Lee, R. A. Flavell, Role of OCT-1 and partner proteins in T cell differentiation. *Biochim Biophys Acta* **1859**, 825-831 (2016).

346. V. Goffin *et al.*, Transcription factor binding sites in the pol gene intragenic regulatory region of HIV-1 are important for virus infectivity. *Nucleic Acids Res* **33**, 4285-4310 (2005).

347. M. L. Nogueira, V. E. Wang, D. Tantin, P. A. Sharp, T. M. Kristie, Herpes simplex virus infections are arrested in Oct-1-deficient cells. *Proc Natl Acad Sci U S A* **101**, 1473-1478 (2004).

348. H. Lin *et al.*, MARCH3 attenuates IL-1beta-triggered inflammation by mediating K48-linked polyubiquitination and degradation of IL-1RI. *Proc Natl Acad Sci U S A* **115**, 12483-12488 (2018).

349. L. Guo *et al.*, Kinase Suppressor of Ras 2 (KSR2) expression in the brain regulates energy balance and glucose homeostasis. *Mol Metab* **6**, 194-205 (2017).

350. C. Gil-Cayuela *et al.*, New Altered Non-Fibrillar Collagens in Human Dilated Cardiomyopathy: Role in the Remodeling Process. *PLoS One* **11**, e0168130 (2016).

351. Q. Wang, L. Su, Vpr Enhances HIV-1 Env Processing and Virion Infectivity in Macrophages by Modulating TET2-Dependent IFITM3 Expression. *mBio* **10**, e01344-01319 (2019).

352. F. Corponi *et al.*, Genetic basis of psychopathological dimensions shared between schizophrenia and bipolar disorder. *Prog Neuropsychopharmacol Biol Psychiatry* **89**, 23-29 (2019).

353. M. Mutso *et al.*, Mutation of CD2AP and SH3KBP1 Binding Motif in Alphavirus nsP3 Hypervariable Domain Results in Attenuated Virus. *Viruses* **10**, 226 (2018).

354. F. Zhang *et al.*, MicroRNA-132-3p suppresses type I IFN response through targeting IRF1 to facilitate H1N1 influenza A virus infection. *Biosci Rep* **39**, BSR20192769 (2019).

355. X. N. Zhang, J. X. Liu, Y. W. Hu, H. Chen, Z. H. Yuan, Hyper-activated IRF-1 and STAT1 contribute to enhanced interferon stimulated gene (ISG) expression by interferon alpha and gamma co-treatment in human hepatoma cells. *Biochim Biophys Acta* **1759**, 417-425 (2006).

356. J. L. Stern, J. Z. Cao, J. Xu, E. S. Mocarski, B. Slobedman, Repression of human cytomegalovirus major immediate early gene expression by the cellular transcription factor CCAAT displacement protein. *Virology* **378**, 214-225 (2008).

357. K. Nystrom *et al.*, Inosine Triphosphate Pyrophosphatase Dephosphorylates Ribavirin Triphosphate and Reduced Enzymatic Activity Potentiates Mutagenesis in Hepatitis C Virus. *J Virol* **92**, e01087-01018 (2018).

358. A. T. N. Tebbenkamp *et al.*, The 7q11.23 Protein DNAJC30 Interacts with ATP Synthase and Links Mitochondria to Brain Development. *Cell* **175**, 1088-1104 e1023 (2018).

359. L. Maksumova *et al.*, Protein tyrosine phosphatase alpha regulates Fyn activity and Cbp/PAG phosphorylation in thymocyte lipid rafts. *J Immunol* **175**, 7947-7956 (2005).

360. S. Z. Li *et al.*, Phosphorylation of MAVS/VISA by Nemo-like kinase (NLK) for degradation regulates the antiviral innate immune response. *Nat Commun* **10**, 3233 (2019).

361. A. Mohammadipoor, R. H. Lee, D. J. Prockop, T. J. Bartosh, Stanniocalcin-1 attenuates ischemic cardiac injury and response of differentiating monocytes/macrophages to inflammatory stimuli. *Transl Res* **177**, 127-142 (2016).

362. H. Fan *et al.*, Attachment and Postattachment Receptors Important for Hepatitis C Virus Infection and Cell-to-Cell Transmission. *J Virol* **91**, e00280-00217 (2017).

363. G. Long *et al.*, Antianemia Drug Roxadustat (FG-4592) Protects Against Doxorubicin-Induced Cardiotoxicity by Targeting Antiapoptotic and Antioxidative Pathways. *Front Pharmacol* **11**, 1191 (2020).

364. W. Wang, Y. Jin, N. Zeng, Q. Ruan, F. Qian, SOD2 Facilitates the Antiviral Innate Immune Response by Scavenging Reactive Oxygen Species. *Viral Immunol* **30**, 582-589 (2017).

365. B. Ghoujal, M. P. Milev, L. Ajamian, K. Abel, A. J. Mouland, ESCRT-II's involvement in HIV-1 genomic RNA trafficking and assembly. *Biol Cell* **104**, 706-721 (2012).

366. S. B. Hwang, J. P. Burbach, C. Chang, TR4 orphan receptor crosstalks to chicken ovalbumin upstream protein-transcription factor and thyroid hormone receptor to induce the transcriptional activity of the human immunodeficiency virus type 1 long-terminal repeat. *Endocrine* **8**, 169-175 (1998).

367. W. J. Lin *et al.*, Suppression of hepatitis B virus core promoter by the nuclear orphan receptor TR4. *J Biol Chem* **278**, 9353-9360 (2003).

368. B. Briard, D. E. Place, T. D. Kanneganti, DNA Sensing in the Innate Immune Response. *Physiology (Bethesda)* **35**, 112-124 (2020).

369. R. Matkovic *et al.*, The Host DHX9 DExH-Box Helicase Is Recruited to Chikungunya Virus Replication Complexes for Optimal Genomic RNA Translation. *J Virol* **93**, e01764-01718 (2019).

370. S. Brady *et al.*, Virion-associated, host-derived DHX9/RNA helicase A enhances the processivity of HIV-1 reverse transcriptase on genomic RNA. *J Biol Chem* **294**, 11473-11485 (2019).

371. C. Orlandi, G. Forlani, G. Tosi, R. S. Accolla, Molecular and cellular correlates of the CIITA-mediated inhibition of HTLV-2 Tax-2 transactivator function resulting in loss of viral replication. *J Transl Med* **9**, 106 (2011).

372. T. Guan *et al.*, ZEB1, ZEB2, and the miR-200 family form a counterregulatory network to regulate CD8(+) T cell fates. *J Exp Med* **215**, 1153-1168 (2018).

373. M. Shao *et al.*, Exogenous angiotensin (1-7) directly inhibits epithelial-mesenchymal transformation induced by transforming growth factor-beta1 in alveolar epithelial cells. *Biomed Pharmacother* **117**, 109193 (2019).

374. C. A. Stewart *et al.*, SARS-CoV-2 infection induces EMT-like molecular changes, including ZEB1-mediated repression of the viral receptor ACE2, in lung cancer models. *bioRxiv*, (2020).

375. V. Audrito, V. G. Messana, S. Deaglio, NAMPT and NAPRT: Two Metabolic Enzymes With Key Roles in Inflammation. *Front Oncol* **10**, 358 (2020).

376. W. Dantoft, K. A. Robertson, W. J. Watkins, B. Strobl, P. Ghazal, Metabolic Regulators Nampt and Sirt6 Serially Participate in the Macrophage Interferon Antiviral Cascade. *Front Microbiol* **10**, 355 (2019).

377. H. Pellkofer *et al.*, Modelling paraneoplastic CNS disease: T-cells specific for the onconeuronal antigen PNMA1 mediate autoimmune encephalomyelitis in the rat. *Brain* **127**, 1822-1830 (2004).

378. K. F. Robinson, S. D. Narasipura, J. Wallace, E. M. Ritz, L. Al-Harthi, Negative regulation of IL-8 in human astrocytes depends on beta-catenin while positive regulation is mediated by TCFs/LEF/ATF2 interaction. *Cytokine* **136**, 155252 (2020).

379. C. Gazin, ZFX transactivation of the HIV-1 LTR is cell specific and depends on core enhancer and TATA box sequences. *Nucleic Acids Res* **27**, 2156-2164 (1999).

380. E. Yoshihara *et al.*, Thioredoxin/Txnip: redoxisome, as a redox switch for the pathogenesis of diseases. *Front Immunol* **4**, 514 (2014).

381. J. A. Coleman *et al.*, Phospholipid flippase ATP8A2 is required for normal visual and auditory function and photoreceptor and spiral ganglion cell survival. *J Cell Sci* **127**, 1138-1149 (2014).

382. D. N. Zhou, Y. F. Deng, R. H. Li, P. Yin, C. S. Ye, Concurrent alterations of RAGE, RECK, and MMP9 protein expression are relevant to Epstein-Barr virus infection, metastasis, and survival in nasopharyngeal carcinoma. *Int J Clin Exp Pathol* **7**, 3245-3254 (2014).

383. S. T. Ong *et al.*, Phosphorylation of Rab5a protein by protein kinase C is crucial for T-cell migration. *J Biol Chem* **289**, 19420-19434 (2014).

384. M. Li *et al.*, Characterization of hepatitis B virus infection and viral DNA integration in non-Hodgkin lymphoma. *Int J Cancer* **147**, 2199-2209 (2020).

385. H. H. Wang *et al.*, Typical gene expression profile of pseudorabies virus reactivation from latency in swine trigeminal ganglion. *J Neurovirol* **26**, 687-695 (2020).

386. H. Boruchowicz, J. Hawkins, K. Cruz-Palomar, R. Lippe, The XPO6 Exportin Mediates Herpes Simplex Virus 1 gM Nuclear Release Late in Infection. *J Virol* **94**, e00753-00720 (2020).

387. A. S. Upadhyay *et al.*, Cellular requirements for iron-sulfur cluster insertion into the antiviral radical SAM protein viperin. *J Biol Chem* **292**, 13879-13889 (2017).

388. J. Li, Z. Xu, Y. Zheng, D. L. Johnson, J. H. Ou, Regulation of hepatocyte nuclear factor 1 activity by wild-type and mutant hepatitis B virus X proteins. *J Virol* **76**, 5875-5881 (2002).

389. A. J. Smith *et al.*, Host genes associated with HIV-1 replication in lymphatic tissue. *J Immunol* **185**, 5417-5424 (2010).

390. I. M. Dykes *et al.*, HIC2 is a novel dosage-dependent regulator of cardiac development located within the distal 22q11 deletion syndrome region. *Circ Res* **115**, 23-31 (2014).

391. E. Muscolino *et al.*, Herpesviruses induce aggregation and selective autophagy of host signalling proteins NEMO and RIPK1 as an immune-evasion mechanism. *Nat Microbiol* **5**, 331-342 (2020).

392. J. Xie, E. N. Heim, M. Crite, D. DiMaio, TBC1D5-Catalyzed Cycling of Rab7 Is Required for Retromer-Mediated Human Papillomavirus Trafficking during Virus Entry. *Cell Rep* **31**, 107750 (2020).

393. N. T. Hertz *et al.*, Neuronally Enriched RUFY3 Is Required for Caspase-Mediated Axon Degeneration. *Neuron* **103**, 412-422 e414 (2019).

394. J. Hsu *et al.*, Genetic Control of Left Atrial Gene Expression Yields Insights into the Genetic Susceptibility for Atrial Fibrillation. *Circ Genom Precis Med* **11**, e002107 (2018).

395. Y. Nakamura *et al.*, Altered expression of a novel cellular gene as a consequence of integration of human T cell lymphotropic virus type 1. *J Gen Virol* **75 (Pt 10)**, 2625-2633 (1994).

396. I. Rhee, M. C. Zhong, B. Reizis, C. Cheong, A. Veillette, Control of dendritic cell migration, T cell-dependent immunity, and autoimmunity by protein tyrosine phosphatase PTPN12 expressed in dendritic cells. *Mol Cell Biol* **34**, 888-899 (2014).

397. M. A. Yenari *et al.*, Calbindin d28k overexpression protects striatal neurons from transient focal cerebral ischemia. *Stroke* **32**, 1028-1035 (2001).

398. A. G. Diop, M. Lesort, F. Esclaire, M. Dumas, J. Hugon, Calbindin D28K-containing neurons, and not HSP70-expressing neurons, are more resistant to HIV-1 envelope (gp120) toxicity in cortical cell cultures. *J Neurosci Res* **42**, 252-258 (1995).

399. R. Wash *et al.*, Permissive and restricted virus infection of murine embryonic stem cells. *J Gen Virol* **93**, 2118-2130 (2012).

400. H. H. Xue *et al.*, The transcription factor GABP is a critical regulator of B lymphocyte development. *Immunity* **26**, 421-431 (2007).

401. N. Sun *et al.*, TRIM35 mediates protection against influenza infection by activating TRAF3 and degrading viral PB2. *Protein Cell* **11**, 894-914 (2020).

402. H. Ma *et al.*, A CRISPR-Based Screen Identifies Genes Essential for West-Nile-Virus-Induced Cell Death. *Cell Rep* **12**, 673-683 (2015).

403. R. L. Caldwell, K. B. Lane, V. L. Shepherd, HIV-1 Tat interaction with cyclin T1 represses mannose receptor and the bone morphogenetic protein receptor-2 transcription. *Arch Biochem Biophys* **449**, 27-33 (2006).

404. A. Hanna, N. G. Frangogiannis, The Role of the TGF-beta Superfamily in Myocardial Infarction. *Front Cardiovasc Med* **6**, 140 (2019).

405. T. E. Thayer *et al.*, The Role of Bone Morphogenetic Protein Signaling in Non-Alcoholic Fatty Liver Disease. *Sci Rep* **10**, 9831 (2020).

406. L. Xu *et al.*, High-Dose Dexamethasone Manipulates the Tumor Microenvironment and Internal Metabolic Pathways in Anti-Tumor Progression. *Int J Mol Sci* **21**, 184 (2020).

407. M. Butler *et al.*, Cyclin-Dependent Kinases 8 and 19 Regulate Host Cell Metabolism during Dengue Virus Serotype 2 Infection. *Viruses* **12**, 654 (2020).

408. S. Funakoshi *et al.*, BILL-cadherin/cadherin-17 contributes to the survival of memory B cells. *PLoS One* **10**, e0117566 (2015).

409. O. Sharmin *et al.*, Activation of GPR35 protects against cerebral ischemia by recruiting monocyte-derived macrophages. *Sci Rep* **10**, 9400 (2020).

410. K. Chen *et al.*, Inhibition of GPR35 Preserves Mitochondrial Function After Myocardial Infarction by Targeting Calpain 1/2. *J Cardiovasc Pharmacol* **75**, 556-563 (2020).

411. A. Takakuwa *et al.*, Butyric Acid and Leucine Induce alpha-Defensin Secretion from Small Intestinal Paneth Cells. *Nutrients* **11**, 2817 (2019).

412. G. Wang *et al.*, The G Protein-Coupled Receptor FFAR2 Promotes Internalization during Influenza A Virus Entry. *J Virol* **94**, e01707-01719 (2020).

413. C. Wang *et al.*, Sphingomyelin synthase 1 enhances BCR signaling to promote lupus-like autoimmune response. *EBioMedicine* **45**, 578-587 (2019).

414. M. Taniguchi *et al.*, Sphingomyelin generated by sphingomyelin synthase 1 is involved in attachment and infection with Japanese encephalitis virus. *Sci Rep* **6**, 37829 (2016).

415. M. Maghsoudloo, S. Azimzadeh Jamalkandi, A. Najafi, A. Masoudi-Nejad, An efficient hybrid feature selection method to identify potential biomarkers in common chronic lung inflammatory diseases. *Genomics* **112**, 3284-3293 (2020).

416. Q. Wang, T. M. Young, M. B. Mathews, T. Pe'ery, Developmental regulators containing the I-mfa domain interact with T cyclins and Tat and modulate transcription. *J Mol Biol* **367**, 630-646 (2007).

417. S. Liong, R. Lim, G. Barker, M. Lappas, Hepatitis A virus cellular receptor 2 (HAVCR2) is decreased with viral infection and regulates pro-labour mediators OA. *Am J Reprod Immunol* **78**, e12696 (2017).

418. R. Kim *et al.*, Genome-based identification of cancer genes by proviral tagging in mouse retrovirus-induced T-cell lymphomas. *J Virol* **77**, 2056-2062 (2003).

419. M. Adamiak, M. Z. Ratajczak, Innate Immunity and Mobilization of Hematopoietic Stem Cells. *Curr Stem Cell Rep* **3**, 172-180 (2017).

420. A. Saeed *et al.*, Mannan binding lectin-associated serine protease 1 is induced by hepatitis C virus infection and activates human hepatic stellate cells. *Clin Exp Immunol* **174**, 265-273 (2013).

421. H. L. Zenner, S. Yoshimura, F. A. Barr, C. M. Crump, Analysis of Rab GTPase-activating proteins indicates that Rab1a/b and Rab43 are important for herpes simplex virus 1 secondary envelopment. *J Virol* **85**, 8012-8021 (2011).

422. T. Pechenick Jowers *et al.*, RAB1A promotes Vaccinia virus replication by facilitating the production of intracellular enveloped virions. *Virology* **475**, 66-73 (2015).

423. J. Lin *et al.*, Rab1A is required for assembly of classical swine fever virus particle. *Virology* **514**, 18-29 (2018).

424. B. G. G. Pinto *et al.*, ACE2 Expression is Increased in the Lungs of Patients with Comorbidities Associated with Severe COVID-19. *medRxiv*, (2020).

425. M. de Oliveira *et al.*, Irisin modulates genes associated with severe coronavirus disease (COVID-19) outcome in human subcutaneous adipocytes cell culture. *Mol Cell Endocrinol* **515**, 110917 (2020).

426. K. Ishikawa-Sasaki, S. Nagashima, K. Taniguchi, J. Sasaki, Model of OSBP-Mediated Cholesterol Supply to Aichi Virus RNA Replication Sites Involving Protein-Protein Interactions among Viral Proteins, ACBD3, OSBP, VAP-A/B, and SAC1. *J Virol* **92**, e01952-01917 (2018).

427. C. Whitten-Bauer *et al.*, The Host Factor Erlin-1 is Required for Efficient Hepatitis C Virus Infection. *Cells* **8**, 1555 (2019).

428. Z. A. Felton-Edkins *et al.*, Epstein-Barr virus induces cellular transcription factors to allow active expression of EBER genes by RNA polymerase III. *J Biol Chem* **281**, 33871-33880 (2006).

429. M. D. J. Dicks *et al.*, Multiple components of the nuclear pore complex interact with the amino-terminus of MX2 to facilitate HIV-1 restriction. *PLoS Pathog* **14**, e1007408 (2018).

430. J. Fernandez *et al.*, Transportin-1 binds to the HIV-1 capsid via a nuclear localization signal and triggers uncoating. *Nat Microbiol* **4**, 1840-1850 (2019).

431. A. Darbyson, J. K. Ngsee, Oxysterol-binding protein ORP3 rescues the Amyotrophic Lateral Sclerosis-linked mutant VAPB phenotype. *Exp Cell Res* **341**, 18-31 (2016).

432. G. R. Juszczak, A. M. Stankiewicz, Glucocorticoids, genes and brain function. *Prog Neuropsychopharmacol Biol Psychiatry* **82**, 136-168 (2018).

433. Y. Gao, G. X. Qi, L. Guo, Y. X. Sun, Bioinformatics Analyses of Differentially Expressed Genes Associated with Acute Myocardial Infarction. *Cardiovasc Ther* **34**, 67-75 (2016).

434. J. A. Zepp *et al.*, TRAF4-SMURF2-mediated DAZAP2 degradation is critical for IL-25 signaling and allergic airway inflammation. *J Immunol* **194**, 2826-2837 (2015).

435. S. L. Fu *et al.*, Hippo signaling pathway in lung development, regeneration, and diseases. *Yi Chuan* **39**, 597-606 (2017).

436. T. Bertero *et al.*, Vascular stiffness mechanoactivates YAP/TAZ-dependent glutaminolysis to drive pulmonary hypertension. *J Clin Invest* **126**, 3313-3335 (2016).

437. D. Kandilya *et al.*, Zika virus alters DNA methylation status of genes involved in Hippo signaling pathway in human neural progenitor cells. *Epigenomics* **11**, 1143-1161 (2019).

438. L. S. Hall *et al.*, Cis-effects on gene expression in the human prenatal brain associated with genetic risk for neuropsychiatric disorders. *Mol Psychiatry*, (2020).

439. P. Zhou *et al.*, In vivo discovery of immunotherapy targets in the tumour microenvironment. *Nature* **506**, 52-57 (2014).

440. N. E. Savaskan, A. U. Brauer, R. Nitsch, Molecular cloning and expression regulation of PRG-3, a new member of the plasticity-related gene family. *Eur J Neurosci* **19**, 212-220 (2004).

441. F. El-Asmi *et al.*, Cross-talk between SUMOylation and ISGylation in response to interferon. *Cytokine* **129**, 155025 (2020).

442. T. J. LaRocca *et al.*, Pharmacological Silencing of MicroRNA-152 Prevents Pressure Overload-Induced Heart Failure. *Circ Heart Fail* **13**, e006298 (2020).

443. V. Bhatt *et al.*, Structural basis of host protein hijacking in human T-cell leukemia virus integration. *Nat Commun* **11**, 3121 (2020).

444. Y. Feng *et al.*, Inhibitory effect of HMGN2 protein on human hepatitis B virus expression and replication in the HepG2.2.15 cell line. *Antiviral Res* **81**, 277-282 (2009).

445. K. Dybkaer *et al.*, Genome wide transcriptional analysis of resting and IL2 activated human natural killer cells: gene expression signatures indicative of novel molecular signaling pathways. *BMC Genomics* **8**, 230 (2007).

446. N. Boutaoui *et al.*, Epigenome-wide effects of vitamin D on asthma bronchial epithelial cells. *Epigenetics* **14**, 844-849 (2019).

447. S. A. Snider *et al.*, Choline transport links macrophage phospholipid metabolism and inflammation. *J Biol Chem* **293**, 11600-11611 (2018).

448. K. Gobeil Odai *et al.*, In Vitro Hepatitis C Virus Infection and Hepatic Choline Metabolism. *Viruses* **12**, 108 (2020).

449. S. G. Tangye, K. E. Nichols, N. J. Hare, B. C. van de Weerdt, Functional requirements for interactions between CD84 and Src homology 2 domain-containing proteins and their contribution to human T cell activation. *J Immunol* **171**, 2485-2495 (2003).

450. S. F. Li *et al.*, Downregulation of beta1,4-galactosyltransferase 5 improves insulin resistance by promoting adipocyte commitment and reducing inflammation. *Cell Death Dis* **9**, 196 (2018).

451. Y. N. Dai *et al.*, Serum Proteomic Changes as Candidate Biomarkers of Intermediate Liver Fibrosis in Chronic Hepatitis B Infection. *OMICS* **23**, 167-179 (2019).

452. Z. Alli, Y. Chen, S. Abdul Wajid, B. Al-Saud, M. Abdelhaleem, A role for DHX32 in regulating T-cell apoptosis. *Anticancer Res* **27**, 373-377 (2007).

453. D. Lan *et al.*, Transcriptome-wide association study identifies genetically dysregulated genes in diabetic neuropathy. *Comb Chem High Throughput Screen* **24**, 319-325 (2020).

454. T. C. van der Pouw Kraan *et al.*, Metabolic changes in type 2 diabetes are reflected in peripheral blood cells, revealing aberrant cytotoxicity, a viral signature, and hypoxia inducible factor activity. *BMC Med Genomics* **8**, 20 (2015).

455. S. Wang *et al.*, FoxO1-mediated autophagy is required for NK cell development and innate immunity. *Nat Commun* **7**, 11023 (2016).

456. Y. Huang *et al.*, Autophagy Contributes to Host Immunity and Protection against Zika Virus Infection via Type I IFN Signaling. *Mediators Inflamm* **2020**, 9527147 (2020).

457. M. F. Del Greco *et al.*, Genome-wide association analysis and fine mapping of NT-proBNP level provide novel insight into the role of the MTHFR-CLCN6-NPPA-NPPB gene cluster. *Hum Mol Genet* **20**, 1660-1671 (2011).

458. Q. Zheng *et al.*, Exome-Wide Association Study Reveals Several Susceptibility Genes and Pathways Associated With Acute Coronary Syndromes in Han Chinese. *Front Genet* **11**, 336 (2020).

459. W. Ren *et al.*, Bromodomain protein Brd3 promotes Ifnb1 transcription via enhancing IRF3/p300 complex formation and recruitment to Ifnb1 promoter in macrophages. *Sci Rep* **7**, 39986 (2017).

460. Y. Liu *et al.*, KRAB-Zinc Finger Protein ZNF268a Deficiency Attenuates the Virus-Induced Pro-Inflammatory Response by Preventing IKK Complex Assembly. *Cells* **8**, 1604 (2019).

461. J. Hatterschide *et al.*, PTPN14 degradation by high-risk human papillomavirus E7 limits keratinocyte differentiation and contributes to HPV-mediated oncogenesis. *Proc Natl Acad Sci U S A* **116**, 7033-7042 (2019).

462. D. Aki, W. Zhang, Y. C. Liu, The E3 ligase Itch in immune regulation and beyond. *Immunol Rev* **266**, 6-26 (2015).

463. F. Parvaiz *et al.*, Hepatitis C virus infection: molecular pathways to insulin resistance. *Virol J* **8**, 474 (2011).

464. A. E. Papathanassiu *et al.*, BCAT1 controls metabolic reprogramming in activated human macrophages and is associated with inflammatory diseases. *Nat Commun* **8**, 16040 (2017).

465. M. Honda *et al.*, Malnutrition impairs interferon signaling through mTOR and FoxO pathways in patients with chronic hepatitis C. *Gastroenterology* **141**, 128-140, 140 e121-122 (2011).

466. L. P. Sutton *et al.*, Orphan receptor GPR158 controls stress-induced depression. *Elife* **7**, e33273 (2018).

467. C. Vergara *et al.*, Multi-Ancestry Genome-Wide Association Study of Spontaneous Clearance of Hepatitis C Virus. *Gastroenterology* **156**, 1496-1507 e1497 (2019).

468. Y. C. Patel, Somatostatin and its receptor family. *Front Neuroendocrinol* **20**, 157-198 (1999).

469. H. Zhao *et al.*, Elovl6 Deficiency Improves Glycemic Control in Diabetic db/db Mice by Expanding beta-Cell Mass and Increasing Insulin Secretory Capacity. *Diabetes* **66**, 1833-1846 (2017).

470. K. M. Edenborough *et al.*, Dendritic Cells Generated From Mops condylurus, a Likely Filovirus Reservoir Host, Are Susceptible to and Activated by Zaire Ebolavirus Infection. *Front Immunol* **10**, 2414 (2019).

471. J. Xu, F. Liu, Y. Li, L. Shen, A 1p/19q Codeletion-Associated Immune Signature for Predicting Lower Grade Glioma Prognosis. *Cell Mol Neurobiol*, (2020).

472. J. M. Petrosino, D. Disilvestro, O. Ziouzenkova, Aldehyde dehydrogenase 1A1: friend or foe to female metabolism? *Nutrients* **6**, 950-973 (2014).

473. S. Peter *et al.*, AMPAR Auxiliary Protein SHISA6 Facilitates Purkinje Cell Synaptic Excitability and Procedural Memory Formation. *Cell Rep* **31**, 107515 (2020).

474. J. Cao *et al.*, HDAC11 regulates type I interferon signaling through defatty-acylation of SHMT2. *Proc Natl Acad Sci U S A* **116**, 5487-5492 (2019).

475. I. Khan *et al.*, Modulation of hepatitis C virus genome replication by glycosphingolipids and four-phosphate adaptor protein 2. *J Virol* **88**, 12276-12295 (2014).

476. K. A. Davis, M. Morelli, J. T. Patton, Rotavirus NSP1 Requires Casein Kinase II-Mediated Phosphorylation for Hijacking of Cullin-RING Ligases. *mBio* **8**, (2017).

477. S. Langer *et al.*, The E3 Ubiquitin-Protein Ligase Cullin 3 Regulates HIV-1 Transcription. *Cells* **9**, 2010 (2020).

478. M. Acosta-Herrera *et al.*, Genome-wide meta-analysis reveals shared new loci in systemic seropositive rheumatic diseases. *Ann Rheum Dis* **78**, 311-319 (2019).

479. X. Zhong *et al.*, ZFYVE1 negatively regulates MDA5- but not RIG-I-mediated innate antiviral response. *PLoS Pathog* **16**, e1008457 (2020).

480. T. Seya, H. Oshiumi, M. Sasai, T. Akazawa, M. Matsumoto, TICAM-1 and TICAM-2: toll-like receptor adapters that participate in induction of type 1 interferons. *Int J Biochem Cell Biol* **37**, 524-529 (2005).

481. L. E. Gralinski *et al.*, Allelic Variation in the Toll-Like Receptor Adaptor Protein Ticam2 Contributes to SARS-Coronavirus Pathogenesis in Mice. *G3 (Bethesda)* **7**, 1653-1663 (2017).

482. J. A. Harker, K. A. Wong, A. Dolgoter, E. I. Zuniga, Cell-Intrinsic gp130 Signaling on CD4+ T Cells Shapes Long-Lasting Antiviral Immunity. *J Immunol* **195**, 1071-1081 (2015).

483. F. Tomassoni-Ardori *et al.*, Rbfox1 up-regulation impairs BDNF-dependent hippocampal LTP by dysregulating TrkB isoform expression levels. *Elife* **8**, e49673 (2019).

484. Y. Mohamud *et al.*, Enteroviral Infection Inhibits Autophagic Flux via Disruption of the SNARE Complex to Enhance Viral Replication. *Cell Rep* **22**, 3292-3303 (2018).

485. E. Tijchon, J. Havinga, F. N. van Leeuwen, B. Scheijen, B-lineage transcription factors and cooperating gene lesions required for leukemia development. *Leukemia* **27**, 541-552 (2013).

486. A. C. Stabell *et al.*, Non-human Primate Schlafen11 Inhibits Production of Both Host and Viral Proteins. *PLoS Pathog* **12**, e1006066 (2016).

487. F. Jabot-Hanin *et al.*, An eQTL variant of ZXDC is associated with IFN-gamma production following Mycobacterium tuberculosis antigen-specific stimulation. *Sci Rep* **7**, 12800 (2017).

488. K. L. Fonseca *et al.*, Deficiency in the glycosyltransferase Gcnt1 increases susceptibility to tuberculosis through a mechanism involving neutrophils. *Mucosal Immunol* **13**, 836-848 (2020).

489. Y. Du *et al.*, Elevated semaphorin5A in systemic lupus erythematosus is in association with disease activity and lupus nephritis. *Clin Exp Immunol* **188**, 234-242 (2017).

490. A. Gaaya *et al.*, Plasticity-related gene-1 inhibits lysophosphatidic acid-induced vascular smooth muscle cell migration and proliferation and prevents neointima formation. *Am J Physiol Cell Physiol* **303**, C1104-1114 (2012).

491. R. Zhang *et al.*, Interaction of Epstein-Barr virus genes with human gastric carcinoma transcriptome. *Oncotarget* **8**, 38399-38412 (2017).

492. I. Panagoulias *et al.*, Ets-2 Acts As a Transcriptional Repressor of the Human Immunodeficiency Virus Type 1 through Binding to a Repressor-Activator Target Sequence of 5'-LTR. *Front Immunol* **8**, 1924 (2017).

493. A. Parcelier *et al.*, AF1q/MLLT11 regulates the emergence of human prothymocytes through cooperative interaction with the Notch signaling pathway. *Blood* **118**, 1784-1796 (2011).

494. D. A. Ruhl *et al.*, Synaptotagmin 17 controls neurite outgrowth and synaptic physiology via distinct cellular pathways. *Nat Commun* **10**, 3532 (2019).

495. B. Schnabl *et al.*, Zinc finger protein 267 is up-regulated during the activation process of human hepatic stellate cells and functions as a negative transcriptional regulator of MMP-10. *Biochem Biophys Res Commun* **335**, 87-96 (2005).

496. J. Sanchez-Garrido, A. R. Shenoy, Regulation and repurposing of nutrient sensing and autophagy in innate immunity. *Autophagy*, 1-21 (2020).

497. V. Yuferov *et al.*, Expression of ephrin receptors and ligands in postmortem brains of HIV-infected subjects with and without cognitive impairment. *J Neuroimmune Pharmacol* **8**, 333-344 (2013).

498. H. M. Walline *et al.*, Integration of high-risk human papillomavirus into cellular cancer-related genes in head and neck cancer cell lines. *Head Neck* **39**, 840-852 (2017).

499. O. A. Makeeva *et al.*, Genomic Study of Cardiovascular Continuum Comorbidity. *Acta Naturae* **7**, 89-99 (2015).

500. L. Perrin-Cocon *et al.*, The current landscape of coronavirus-host protein-protein interactions. *J Transl Med* **18**, 319 (2020).

501. J. Zhou *et al.*, Investigation of gene-gene interactions in cardiac traits and serum fatty acid levels in the LURIC Health Study. *PLoS One* **15**, e0238304 (2020).

502. D. Bai *et al.*, Porcine deltacoronavirus (PDCoV) modulates calcium influx to favor viral replication. *Virology* **539**, 38-48 (2020).

503. M. Paquette, M. Fantino, S. Bernard, A. Baass, The ZPR1 genotype predicts myocardial infarction in patients with familial hypercholesterolemia. *J Clin Lipidol* **14**, 660-666 (2020).

504. J. Mei *et al.*, Construction of an immune-related gene signature for prediction of prognosis in patients with cervical cancer. *Int Immunopharmacol* **88**, 106882 (2020).

505. R. Mei *et al.*, Sustained viral response and treatment-induced cytopenia correlate with SLCs and KLF12 genotypes in interferon/ribavirin-treated Chinese chronic hepatitis C patients. *J Gastroenterol Hepatol* **31**, 1489-1497 (2016).

506. S. Jilek *et al.*, Immune responses to JC virus in patients with multiple sclerosis treated with natalizumab: a cross-sectional and longitudinal study. *Lancet Neurol* **9**, 264-272 (2010).

507. R. Tian *et al.*, DeepHPV: a deep learning model to predict human papillomavirus integration sites. *Brief Bioinform*, (2020).

508. R. Kurata, A. Tajima, T. Yonezawa, H. Inoko, TRIM39R, but not TRIM39B, regulates type I interferon response. *Biochem Biophys Res Commun* **436**, 90-95 (2013).

509. Y. Chen, W. Gong, H. Wei, W. Dai, S. Xu, 2019-nCoV may create complications in colon cancer patients with ACE2 expression. *Int J Clin Exp Pathol* **13**, 2305-2311 (2020).

510. N. Tongmuang *et al.*, Suppression of micro1 subunit of the adaptor protein complex 2 reduces dengue virus release. *Virus Genes* **56**, 27-36 (2020).

511. J. P. Lynch *et al.*, Plasmacytoid dendritic cells protect from viral bronchiolitis and asthma through semaphorin 4a-mediated T reg expansion. *J Exp Med* **215**, 537-557 (2018).

512. M. Durocher *et al.*, Inflammatory, regulatory, and autophagy co-expression modules and hub genes underlie the peripheral immune response to human intracerebral hemorrhage. *J Neuroinflammation* **16**, 56 (2019).

513. H. C. H. Yim, T. Y. Y. Leon, J. C. B. Li, MXD1 regulates the H9N2 and H1N1 influenza A virus-induced chemokine expression and their replications in human macrophage. *J Leukoc Biol* **108**, 1631-1640 (2020).

514. G. Vazquez-Ortiz *et al.*, Differentially expressed genes between high-risk human papillomavirus types in human cervical cancer cells. *Int J Gynecol Cancer* **17**, 484-491 (2007).

515. R. Uzhachenko *et al.*, Tumour suppressor Fus1 provides a molecular link between inflammatory response and mitochondrial homeostasis. *J Pathol* **227**, 456-469 (2012).

516. A. Mok *et al.*, Genome-wide profiling identifies associations between lupus nephritis and differential methylation of genes regulating tissue hypoxia and type 1 interferon responses. *Lupus Sci Med* **3**, e000183 (2016).

517. K. S. Yeo *et al.*, JMJD8 is a positive regulator of TNF-induced NF-kappaB signaling. *Sci Rep* **6**, 34125 (2016).

518. T. Michel *et al.*, Increased Th2 cytokine secretion, eosinophilic airway inflammation, and airway hyperresponsiveness in neurturin-deficient mice. *J Immunol* **186**, 6497-6504 (2011).

519. K. Goto *et al.*, Orchestration of Intracellular Circuits by G Protein-Coupled Receptor 39 for Hepatitis B Virus Proliferation. *Int J Mol Sci* **21**, 5661 (2020).

520. M. Frieman *et al.*, Severe acute respiratory syndrome coronavirus ORF6 antagonizes STAT1 function by sequestering nuclear import factors on the rough endoplasmic reticulum/Golgi membrane. *J Virol* **81**, 9812-9824 (2007).

521. Y. Xia *et al.*, Hepatitis B Virus Deregulates the Cell Cycle To Promote Viral Replication and a Premalignant Phenotype. *J Virol* **92**, e00722-00718 (2018).

522. S. Kobayashi *et al.*, Fatty acid-binding protein 3 regulates differentiation of IgM-producing plasma cells. *FEBS J* **288**, 1130-1141 (2020).

523. J. Lin, Y. T. Chen, J. Xia, Q. Yang, MiR674 inhibits the neuraminidase-stimulated immune response on dendritic cells via down-regulated Mbnl3. *Oncotarget* **7**, 48978-48994 (2016).

524. Y. Zhang, D. Y. Leung, E. Goleva, Vitamin D enhances glucocorticoid action in human monocytes: involvement of granulocyte-macrophage colony-stimulating factor and mediator complex subunit 14. *J Biol Chem* **288**, 14544-14553 (2013).

525. A. Ruiz *et al.*, Characterization of the influence of mediator complex in HIV-1 transcription. *J Biol Chem* **289**, 27665-27676 (2014).

526. H. Motaln *et al.*, Human mesenchymal stem cells exploit the immune response mediating chemokines to impact the phenotype of glioblastoma. *Cell Transplant* **21**, 1529-1545 (2012).

527. R. S. Beard, Jr. *et al.*, Palmitoyl acyltransferase DHHC21 mediates endothelial dysfunction in systemic inflammatory response syndrome. *Nat Commun* **7**, 12823 (2016).

528. Q. Zhou, L. Hao, W. Huang, Z. Cai, The Golgi-Associated Plant Pathogenesis-Related Protein GAPR-1 Enhances Type I Interferon Signaling Pathway in Response to Toll-Like Receptor 4. *Inflammation* **39**, 706-717 (2016).

529. L. Zhang, S. Qu, A. Liang, H. Jiang, H. Wang, Gene expression microarray analysis of the sciatic nerve of mice with diabetic neuropathy. *Int J Mol Med* **35**, 333-339 (2015).

530. D. J. Colacurcio, A. Yeager, D. L. Kolson, K. L. Jordan-Sciutto, C. Akay, Calpain-mediated degradation of MDMx/MDM4 contributes to HIV-induced neuronal damage. *Mol Cell Neurosci* **57**, 54-62 (2013).

531. P. Fais *et al.*, Phosphoinositide-specific phospholipase C in normal human liver and in alcohol abuse. *J Cell Biochem* **120**, 7907-7917 (2018).

532. X. Liu *et al.*, MiR-223-3p as a Novel MicroRNA Regulator of Expression of Voltage-Gated K+ Channel Kv4.2 in Acute Myocardial Infarction. *Cell Physiol Biochem* **39**, 102-114 (2016).

533. X. Xu *et al.*, GPCR-mediated PLCbetagamma/PKCbeta/PKD signaling pathway regulates the cofilin phosphatase slingshot 2 in neutrophil chemotaxis. *Mol Biol Cell* **26**, 874-886 (2015).

534. T. V. Varga *et al.*, Novel genetic loci associated with long-term deterioration in blood lipid concentrations and coronary artery disease in European adults. *Int J Epidemiol* **46**, 1211-1222 (2017).

535. M. Costa *et al.*, Lymphocyte gene expression signatures from patients and mouse models of hereditary hemochromatosis reveal a function of HFE as a negative regulator of CD8+ T-lymphocyte activation and differentiation in vivo. *PLoS One* **10**, e0124246 (2015).

536. Z. Pang, R. Raudonis, C. McCormick, Z. Cheng, Early Growth Response 1 Deficiency Protects the Host against Pseudomonas aeruginosa Lung Infection. *Infect Immun* **88**, e00678-00619 (2019).

537. J. Buehler *et al.*, Host signaling and EGR1 transcriptional control of human cytomegalovirus replication and latency. *PLoS Pathog* **15**, e1008037 (2019).

538. D. M. Newman *et al.*, Acetylation of the Cd8 Locus by KAT6A Determines Memory T Cell Diversity. *Cell Rep* **16**, 3311-3321 (2016).

539. Y. Zhang, K. Doyle, M. Bina, Interactions of HTF4 with E-box motifs in the long terminal repeat of human immunodeficiency virus type 1. *J Virol* **66**, 5631-5634 (1992).

540. R. K. Kandasamy *et al.*, A time-resolved molecular map of the macrophage response to VSV infection. *NPJ Syst Biol Appl* **2**, 16027 (2016).

541. S. Yamayoshi *et al.*, Ebola virus matrix protein VP40 uses the COPII transport system for its intracellular transport. *Cell Host Microbe* **3**, 168-177 (2008).

542. P. Yin, Y. Li, L. Zhang, Sec24C-Dependent Transport of Claudin-1 Regulates Hepatitis C Virus Entry. *J Virol* **91**, e00629-00617 (2017).

543. T. Pan *et al.*, Delayed Remote Ischemic Preconditioning ConfersRenoprotection against Septic Acute Kidney Injury via Exosomal miR-21. *Theranostics* **9**, 405-423 (2019).

544. K. P. Maremanda, I. K. Sundar, D. Li, I. Rahman, Age-Dependent Assessment of Genes Involved in Cellular Senescence, Telomere, and Mitochondrial Pathways in Human Lung Tissue of Smokers, COPD, and IPF: Associations With SARS-CoV-2 COVID-19 ACE2-TMPRSS2-Furin-DPP4 Axis. *Front Pharmacol* **11**, 584637 (2020).

545. R. Zou *et al.*, Bioinformatic gene analysis for potential biomarkers and therapeutic targets of atrial fibrillation-related stroke. *J Transl Med* **17**, 45 (2019).

546. G. Mjaess, A. Karam, F. Aoun, S. Albisinni, T. Roumeguere, COVID-19 and the male susceptibility: the role of ACE2, TMPRSS2 and the androgen receptor. *Prog Urol* **30**, 484-487 (2020).

547. G. Liu *et al.*, Calcium-sensing receptor in nutrient sensing: an insight into the modulation of intestinal homoeostasis. *Br J Nutr* **120**, 881-890 (2018).

548. E. K. Halvas *et al.*, HIV-1 viremia not suppressible by antiretroviral therapy can originate from large T cell clones producing infectious virus. *J Clin Invest* **130**, 5847-5857 (2020).

549. J. Zheng *et al.*, Molecular Changes of Lung Malignancy in HIV Infection. *Sci Rep* **8**, 13128 (2018).

550. Y. J. Xu *et al.*, Epstein-Barr virus-coded miR-BART13 promotes nasopharyngeal carcinoma cell growth and metastasis via targeting of the NKIRAS2/NF-kappaB pathway. *Cancer Lett* **447**, 33-40 (2019).

551. Y. Ding, L. Bi, J. Wang, MiR-1180 promotes cardiomyocyte cell cycle re-entry after injury through the NKIRAS2-NFkappaB pathway. *Biochem Cell Biol* **98**, 449-457 (2020).

552. N. Tripathi *et al.*, Human papillomavirus elevated genetic biomarker signature by statistical algorithm. *J Cell Physiol* **235**, 9922-9932 (2020).

553. Y. T. Chen, Y. C. Su, J. T. Kung, B Cell Development sans B Cell Receptor Responsiveness Due to Unfolded Protein Response-Triggered Mef2c Protein Degradation. *J Immunol* **201**, 2885-2898 (2018).

554. N. C. Gassen *et al.*, SKP2 attenuates autophagy through Beclin1-ubiquitination and its inhibition reduces MERS-Coronavirus infection. *Nat Commun* **10**, 5770 (2019).

555. L. Querol, J. Devaux, R. Rojas-Garcia, I. Illa, Autoantibodies in chronic inflammatory neuropathies: diagnostic and therapeutic implications. *Nat Rev Neurol* **13**, 533-547 (2017).

556. H. Dang, Y. Ye, X. Zhao, Y. Zeng, Identification of candidate genes in ischemic cardiomyopathy by gene expression omnibus database. *BMC Cardiovasc Disord* **20**, 320 (2020).

557. S. Bartel *et al.*, Pulmonary microRNA profiles identify involvement of Creb1 and Sec14l3 in bronchial epithelial changes in allergic asthma. *Sci Rep* **7**, 46026 (2017).

558. R. Sumpter, Jr. *et al.*, Fanconi Anemia Proteins Function in Mitophagy and Immunity. *Cell* **165**, 867-881 (2016).

559. X. Wen, L. Casey Klockow, M. Nekorchuk, H. J. Sharifi, C. M. de Noronha, The HIV1 protein Vpr acts to enhance constitutive DCAF1-dependent UNG2 turnover. *PLoS One* **7**, e30939 (2012).

560. P. F. Durrenberger *et al.*, Innate immunity in multiple sclerosis white matter lesions: expression of natural cytotoxicity triggering receptor 1 (NCR1). *J Neuroinflammation* **9**, 1 (2012).

561. X. Ye, T. Zeng, W. Kong, L. L. Chen, Integrative Analyses of Genes Associated with Fulminant Type 1 Diabetes. *J Immunol Res* **2020**, 1025857 (2020).

562. D. Millrine, M. Tei, Y. Gemechu, T. Kishimoto, Rabex-5 is a lenalidomide target molecule that negatively regulates TLR-induced type 1 IFN production. *Proc Natl Acad Sci U S A* **113**, 10625-10630 (2016).

563. A. Pizzini *et al.*, Impact of Vitamin D Deficiency on COVID-19-A Prospective Analysis from the CovILD Registry. *Nutrients* **12**, 2775 (2020).

564. N. Dziuba *et al.*, Identification of cellular proteins required for replication of human immunodeficiency virus type 1. *AIDS Res Hum Retroviruses* **28**, 1329-1339 (2012).

565. M. L. Zheng, X. P. Du, L. Zhao, X. C. Yang, Expression profile of circular RNAs in epicardial adipose tissue in heart failure. *Chin Med J (Engl)* **133**, 2565-2572 (2020).

566. C. Jia, F. Zhang, Y. Zhu, X. Qi, Y. Wang, Public data mining plus domestic experimental study defined involvement of the old-yet-uncharacterized gene matrix-remodeling associated 7 (MXRA7) in physiopathology of the eye. *Gene* **632**, 43-49 (2017).

567. D. Lin *et al.*, Matrix Remodeling Associated 7 Deficiency Alleviates Carbon Tetrachloride-Induced Acute Liver Injury in Mice. *Front Immunol* **9**, 773 (2018).

568. N. J. Lennemann, C. B. Coyne, Dengue and Zika viruses subvert reticulophagy by NS2B3-mediated cleavage of FAM134B. *Autophagy* **13**, 322-332 (2017).

569. M. Sertorio *et al.*, IL-22 and IL-22 binding protein (IL-22BP) regulate fibrosis and cirrhosis in hepatitis C virus and schistosome infections. *Hepatology* **61**, 1321-1331 (2015).

570. H. Li *et al.*, Zika Virus Protease Cleavage of Host Protein Septin-2 Mediates Mitotic Defects in Neural Progenitors. *Neuron* **101**, 1089-1098 e1084 (2019).

571. Z. Zhang, J. Li, T. He, J. Ding, Bioinformatics Identified 17 Immune Genes as Prognostic Biomarkers for Breast Cancer: Application Study Based on Artificial Intelligence Algorithms. *Front Oncol* **10**, 330 (2020).

572. J. Feng *et al.*, RIOK3 is an adaptor protein required for IRF3-mediated antiviral type I interferon production. *J Virol* **88**, 7987-7997 (2014).

573. S. E. Jorgensen *et al.*, STK4 Deficiency Impairs Innate Immunity and Interferon Production Through Negative Regulation of TBK1-IRF3 Signaling. *J Clin Immunol* **41**, 109-124 (2021).

574. M. Rigau *et al.*, Butyrophilin 2A1 is essential for phosphoantigen reactivity by gammadelta T cells. *Science* **367**, eaay5516 (2020).

575. J. G. Purdy, T. Shenk, J. D. Rabinowitz, Fatty acid elongase 7 catalyzes lipidome remodeling essential for human cytomegalovirus replication. *Cell Rep* **10**, 1375-1385 (2015).

576. T. Akagi, H. Ono, H. Nyunoya, K. Shimotohno, Characterization of peripheral blood T-lymphocytes transduced with HTLV-I Tax mutants with different trans-activating phenotypes. *Oncogene* **14**, 2071-2078 (1997).

577. S. Chen *et al.*, Identification of crucial genes in abdominal aortic aneurysm by WGCNA. *PeerJ* **7**, e7873 (2019).

578. J. D. Dougherty, J. P. White, R. E. Lloyd, Poliovirus-mediated disruption of cytoplasmic processing bodies. *J Virol* **85**, 64-75 (2011).

579. A. L. Blasius, P. Krebs, B. M. Sullivan, M. B. Oldstone, D. L. Popkin, Slc15a4, a gene required for pDC sensing of TLR ligands, is required to control persistent viral infection. *PLoS Pathog* **8**, e1002915 (2012).

580. K. Fung *et al.*, Genome-wide association study identifies loci for arterial stiffness index in 127,121 UK Biobank participants. *Sci Rep* **9**, 9143 (2019).

581. M. Gschweitl *et al.*, A SPOPL/Cullin-3 ubiquitin ligase complex regulates endocytic trafficking by targeting EPS15 at endosomes. *Elife* **5**, e13841 (2016).

582. T. Chavalit *et al.*, Hepatitis B Virus-Encoded MicroRNA (HBV-miR-3) Regulates Host Gene PPM1A Related to Hepatocellular Carcinoma. *Microrna* **9**, 232-239 (2020).

583. G. Zeng *et al.*, Cellular and viral miRNA expression in polyomavirus BK infection. *Transpl Infect Dis* **21**, e13159 (2019).

584. H. Lin *et al.*, Cloning and characterization of IL-1HY2, a novel interleukin-1 family member. *J Biol Chem* **276**, 20597-20602 (2001).

585. M. S. Miller *et al.*, Senataxin suppresses the antiviral transcriptional response and controls viral biogenesis. *Nat Immunol* **16**, 485-494 (2015).

586. N. Messal *et al.*, Differential role for CD277 as a co-regulator of the immune signal in T and NK cells. *Eur J Immunol* **41**, 3443-3454 (2011).

587. M. K. Viken *et al.*, Reproducible association with type 1 diabetes in the extended class I region of the major histocompatibility complex. *Genes Immun* **10**, 323-333 (2009).

588. J. Ampuero *et al.*, Fine-mapping butyrophilin family genes revealed several polymorphisms influencing viral genotype selection in hepatitis C infection. *Genes Immun* **16**, 297-300 (2015).

589. E. M. Eriksson *et al.*, Differential expression of CD96 surface molecule represents CD8(+) T cells with dissimilar effector function during HIV-1 infection. *PLoS One* **7**, e51696 (2012).

590. C. K. Rane *et al.*, Development of solitary chemosensory cells in the distal lung after severe influenza injury. *Am J Physiol Lung Cell Mol Physiol* **316**, L1141-L1149 (2019).

591. S. K. Iyengar *et al.*, Genome-Wide Association and Trans-ethnic Meta-Analysis for Advanced Diabetic Kidney Disease: Family Investigation of Nephropathy and Diabetes (FIND). *PLoS Genet* **11**, e1005352 (2015).

592. C. Turk, S. Turk, U. Y. Malkan, I. C. Haznedaroglu, Three critical clinicobiological phases of the human SARS-associated coronavirus infections. *Eur Rev Med Pharmacol Sci* **24**, 8606-8620 (2020).

593. M. Yamamoto *et al.*, Plexin-A4 negatively regulates T lymphocyte responses. *Int Immunol* **20**, 413-420 (2008).

594. S. J. van Vliet *et al.*, Human T cell activation results in extracellular signal-regulated kinase (ERK)-calcineurin-dependent exposure of Tn antigen on the cell surface and binding of the macrophage galactose-type lectin (MGL). *J Biol Chem* **288**, 27519-27532 (2013).

595. J. Yan *et al.*, HIV-1 Vpr Reprograms CLR4(DCAF1) E3 Ubiquitin Ligase to Antagonize Exonuclease 1-Mediated Restriction of HIV-1 Infection. *mBio* **9**, e01732-01718 (2018).

596. P. M. A. Sleiman, M. March, H. Hakonarson, The genetic basis of eosinophilic esophagitis. *Best Pract Res Clin Gastroenterol* **29**, 701-707 (2015).

597. S. R. Brenner, Erythropoietin-induced hemoglobin subunit beta may stimulate innate immune RNA virus pattern recognition, suppress reactive oxygen species, reduce ACE2 viral doorway opening, and neutrophil extracellular traps against COVID-19. *J Med Virol* **93**, 180-181 (2020).

598. M. H. Tsai, C. K. Lee, STAT3 Cooperates With Phospholipid Scramblase 2 to Suppress Type I Interferon Response. *Front Immunol* **9**, 1886 (2018).

599. W. Min *et al.*, Microarray analysis identifies differentially expressed genes induced by human papillomavirus type 18 E6 silencing RNA. *Int J Gynecol Cancer* **19**, 547-563 (2009).

600. A. Terranegra *et al.*, Glucagon-like peptide-1 receptor and sarcoglycan delta genetic variants can affect cardiovascular risk in chronic kidney disease patients under hemodialysis. *Clin Kidney J* **13**, 666-673 (2020).

601. I. D. Aziati *et al.*, PATZ1 is required for efficient HIV-1 infection. *Biochem Biophys Res Commun* **514**, 538-544 (2019).

602. R. M. El-Sheikh *et al.*, Carbamoyl phosphate synthetase 1 (CPS1) as a prognostic marker in chronic hepatitis C infection. *APMIS* **127**, 93-105 (2019).

603. L. L. Su, H. Iwai, J. T. Lin, C. G. Fathman, The transmembrane E3 ligase GRAIL ubiquitinates and degrades CD83 on CD4 T cells. *J Immunol* **183**, 438-444 (2009).

604. G. Song *et al.*, E3 ubiquitin ligase RNF128 promotes innate antiviral immunity through K63-linked ubiquitination of TBK1. *Nat Immunol* **17**, 1342-1351 (2016).

605. S. You *et al.*, Therapeutic use of a selective S1P1 receptor modulator ponesimod in autoimmune diabetes. *PLoS One* **8**, e77296 (2013).

606. N. G. Bazan, Docosanoids and elovanoids from omega-3 fatty acids are pro-homeostatic modulators of inflammatory responses, cell damage and neuroprotection. *Mol Aspects Med* **64**, 18-33 (2018).

607. J. Wang *et al.*, Retinoblastoma binding protein 4 represses HIV-1 long terminal repeat-mediated transcription by recruiting NR2F1 and histone deacetylase. *Acta Biochim Biophys Sin (Shanghai)* **51**, 934-944 (2019).

608. J. Gilissen, F. Jouret, B. Pirotte, J. Hanson, Insight into SUCNR1 (GPR91) structure and function. *Pharmacol Ther* **159**, 56-65 (2016).

609. X. Li *et al.*, GPR91, a critical signaling mechanism in modulating pathophysiologic processes in chronic illnesses. *FASEB J* **34**, 13091-13105 (2020).

610. J. H. Girsch *et al.*, Exocytosis of Progeny Infectious Varicella-Zoster Virus Particles via a Mannose-6-Phosphate Receptor Pathway without Xenophagy following Secondary Envelopment. *J Virol* **94**, e00800-00820 (2020).

611. M. A. Diaz-Salinas, L. A. Casorla, T. Lopez, S. Lopez, C. F. Arias, Most rotavirus strains require the cation-independent mannose-6-phosphate receptor, sortilin-1, and cathepsins to enter cells. *Virus Res* **245**, 44-51 (2018).

612. J. Dheekollu, H. S. Chen, K. M. Kaye, P. M. Lieberman, Timeless-dependent DNA replication-coupled recombination promotes Kaposi's Sarcoma-associated herpesvirus episome maintenance and terminal repeat stability. *J Virol* **87**, 3699-3709 (2013).

613. L. Yong, B. Guang, L. Yan, Bioinformatic analysis of differentially expressed genes involved in the hepatitis B virus-associated acute liver failure. *Acta Gastroenterol Belg* **81**, 288-294 (2018).

614. X. Wang *et al.*, Pulmonary vascular endothelial injury and acute pulmonary hypertension caused by COVID-19: the fundamental cause of refractory hypoxemia? *Cardiovasc Diagn Ther* **10**, 892-897 (2020).

615. R. Zhang *et al.*, Differentially expressed lncRNAs, miRNAs and mRNAs with associated ceRNA networks in a mouse model of myocardial ischemia/reperfusion injury. *Mol Med Rep* **22**, 2487-2495 (2020).

616. Y. Lee *et al.*, ATXN1 protein family and CIC regulate extracellular matrix remodeling and lung alveolarization. *Dev Cell* **21**, 746-757 (2011).

617. C. Zhu *et al.*, EFTUD2 Is a Novel Innate Immune Regulator Restricting Hepatitis C Virus Infection through the RIG-I/MDA5 Pathway. *J Virol* **89**, 6608-6618 (2015).

618. M. F. Chedid *et al.*, Interaction of HSD11B1 and H6PD polymorphisms in subjects with type 2 diabetes are protective factors against obesity: a cross-sectional study. *Diabetol Metab Syndr* **11**, 78 (2019).

619. M. L. Janket, J. S. DeRicco, L. Borowski, V. Ayyavoo, Human immunodeficiency virus (HIV-1) Vpr induced downregulation of NHE1 induces alteration in intracellular pH and loss of ERM complex in target cells. *Virus Res* **126**, 76-85 (2007).

620. B. C. Carvalho *et al.*, Data in support of Rap2a GTPase expression, activation and effects in LPS-mediated innate immune response and NF-kappaB activation. *Data Brief* **24**, 103965 (2019).

621. G. F. Ren, L. L. Xiao, X. J. Ma, Y. S. Yan, P. F. Jiao, Metformin Decreases Insulin Resistance in Type 1 Diabetes Through Regulating p53 and RAP2A in vitro and in vivo. *Drug Des Devel Ther* **14**, 2381-2392 (2020).

622. X. Ye *et al.*, HIV-1 Tat inhibits EAAT-2 through AEG-1 upregulation in models of HIV-associated neurocognitive disorder. *Oncotarget* **8**, 39922-39934 (2017).

623. C. H. van den Kieboom *et al.*, Nasopharyngeal gene expression, a novel approach to study the course of respiratory syncytial virus infection. *Eur Respir J* **45**, 718-725 (2015).

624. P. Mendoza *et al.*, R-Ras2 is required for germinal center formation to aid B cells during energetically demanding processes. *Sci Signal* **11**, eaal1506 (2018).

625. G. D. Kim, H. P. Ng, E. R. Chan, G. H. Mahabeleshwar, Kruppel-like factor 6 promotes macrophage inflammatory and hypoxia response. *FASEB J* **34**, 3209-3223 (2020).

626. A. Bakre *et al.*, Human respiratory syncytial virus non-structural protein NS1 modifies miR-24 expression via transforming growth factor-beta. *J Gen Virol* **96**, 3179-3191 (2015).

627. G. M. Liu, H. D. Zeng, C. Y. Zhang, J. W. Xu, Key genes associated with diabetes mellitus and hepatocellular carcinoma. *Pathol Res Pract* **215**, 152510 (2019).

628. A. Boeske *et al.*, Direct binding to GABARAP family members is essential for HIV-1 Nef plasma membrane localization. *Sci Rep* **7**, 5979 (2017).

629. Z. Ma, Y. Liu, Z. Hao, X. Hua, W. Li, DNA hypermethylation of aurora kinase A in hepatitis C viruspositive hepatocellular carcinoma. *Mol Med Rep* **20**, 2519-2532 (2019).

630. J. Pang *et al.*, Discovery and Validation of Prognostic Biomarker Models to Guide Triage among Adult Dengue Patients at Early Infection. *PLoS One* **11**, e0155993 (2016).

631. A. Alkelai *et al.*, A role for TENM1 mutations in congenital general anosmia. *Clin Genet* **90**, 211-219 (2016).

632. L. Cao *et al.*, HIPK2 is necessary for type I interferon-mediated antiviral immunity. *Sci Signal* **12**, eaau4604 (2019).

633. A. Diab *et al.*, FOXM1 drives HPV+ HNSCC sensitivity to WEE1 inhibition. *Proc Natl Acad Sci U S A* **117**, 28287-28296 (2020).

634. X. Wei *et al.*, NCOA2 promotes lytic reactivation of Kaposi's sarcoma-associated herpesvirus by enhancing the expression of the master switch protein RTA. *PLoS Pathog* **15**, e1008160 (2019).

635. Y. Yang *et al.*, Development Of A Three-Gene Prognostic Signature For Hepatitis B Virus Associated Hepatocellular Carcinoma Based On Integrated Transcriptomic Analysis. *J Cancer* **9**, 1989-2002 (2018).

636. S. Cao *et al.*, The Gut Epithelial Receptor LRRC19 Promotes the Recruitment of Immune Cells and Gut Inflammation. *Cell Rep* **14**, 695-707 (2016).

637. A. Y. F. Li Yim *et al.*, Peripheral blood methylation profiling of female Crohn's disease patients. *Clin Epigenetics* **8**, 65 (2016).

638. G. C. Ippolito *et al.*, Dendritic cell fate is determined by BCL11A. *Proc Natl Acad Sci U S A* **111**, E998-1006 (2014).

639. S. Shrivastav *et al.*, HIV-1 Vpr enhances PPARbeta/delta-mediated transcription, increases PDK4 expression, and reduces PDC activity. *Mol Endocrinol* **27**, 1564-1576 (2013).

640. S. C. Verma, Q. Cai, E. Kreider, J. Lu, E. S. Robertson, Comprehensive analysis of LANA interacting proteins essential for viral genome tethering and persistence. *PLoS One* **8**, e74662 (2013).

641. P. E. Lapinski, J. A. Oliver, J. N. Bodie, F. Marti, P. D. King, The T-cell-specific adapter protein family: TSAd, ALX, and SH2D4A/SH2D4B. *Immunol Rev* **232**, 240-254 (2009).

642. A. Kuhl *et al.*, Myofibrillar myopathy with arrhythmogenic right ventricular cardiomyopathy 7: corroboration and narrowing of the critical region on 10q22.3. *Eur J Hum Genet* **16**, 367-373 (2008).

643. B. Yeganeh *et al.*, Autophagy activation is required for influenza A virus-induced apoptosis and replication. *Biochim Biophys Acta Mol Cell Res* **1865**, 364-378 (2018).

644. A. Lepelley *et al.*, Mutations in COPA lead to abnormal trafficking of STING to the Golgi and interferon signaling. *J Exp Med* **217**, (2020).

645. Q. Liang *et al.*, Integrative identification of Epstein-Barr virus-associated mutations and epigenetic alterations in gastric cancer. *Gastroenterology* **147**, 1350-1362 e1354 (2014).

646. E. Chaudhuri *et al.*, The HIV-1 capsid-binding host factor CPSF6 is post-transcriptionally regulated by the cellular microRNA miR-125b. *J Biol Chem* **295**, 5081-5094 (2020).

647. C. Csortos *et al.*, TIMAP is a positive regulator of pulmonary endothelial barrier function. *Am J Physiol Lung Cell Mol Physiol* **295**, L440-450 (2008).

648. H. Okado, Nervous system regulated by POZ domain Kruppel-like zinc finger (POK) family transcription repressor RP58. *Br J Pharmacol* **178**, 813-826 (2021).

649. T. B. Lear *et al.*, Kelch-like protein 42 is a profibrotic ubiquitin E3 ligase involved in systemic sclerosis. *J Biol Chem* **295**, 4171-4180 (2020).

650. L. J. Sznajder *et al.*, Loss of MBNL1 induces RNA misprocessing in the thymus and peripheral blood. *Nat Commun* **11**, 2022 (2020).

651. A. Banerjee, S. J. Czinn, R. J. Reiter, T. G. Blanchard, Crosstalk between endoplasmic reticulum stress and anti-viral activities: A novel therapeutic target for COVID-19. *Life Sci* **255**, 117842 (2020).

652. R. L. Kuo *et al.*, Proteomics analysis of EV71-infected cells reveals the involvement of host protein NEDD4L in EV71 replication. *J Proteome Res* **14**, 1818-1830 (2015).

653. H. Aga *et al.*, Identification of Novel Potential Type 2 Diabetes Genes Mediating beta-Cell Loss and Hyperglycemia Using Positional Cloning. *Front Genet* **11**, 567191 (2020).

654. S. Perez *et al.*, Dysregulation of the cohesin subunit RAD21 by Hepatitis C virus mediates host-virus interactions. *Nucleic Acids Res* **47**, 2455-2471 (2019).

655. D. A. Chistiakov *et al.*, The TAF5L gene on chromosome 1q42 is associated with type 1 diabetes in Russian affected patients. *Autoimmunity* **38**, 283-293 (2005).

656. H. F. Wang, L. Chen, J. Luo, H. X. He, KLF5 is involved in regulation of IFITM1, 2, and 3 genes during H5N1 virus infection in A549 cells. *Cell Mol Biol (Noisy-le-grand)* **62**, 65-70 (2016).

657. C. V. Forst *et al.*, Integrative gene network analysis identifies key signatures, intrinsic networks and host factors for influenza virus A infections. *NPJ Syst Biol Appl* **3**, 35 (2017).

658. N. Li, J. N. Zhou, Screening and validating the immune-related gene expression signatures in peripheral blood mononuclear cells of nonischaemic cardiomyopathy. *Math Biosci Eng* **17**, 2330-2347 (2020).

659. D. Xiang-Chun *et al.*, Alpha-enolase regulates hepatitis B virus replication through suppression of the interferon signalling pathway. *J Viral Hepat* **25**, 289-295 (2018).

660. C. W. Yang *et al.*, Regulation of T Cell Receptor Signaling by DENND1B in TH2 Cells and Allergic Disease. *Cell* **164**, 141-155 (2016).

661. J. Y. Lee *et al.*, Associations between Genetic Variants and Angiographic Characteristics in Patients with Coronary Artery Disease. *J Atheroscler Thromb* **22**, 363-371 (2015).

662. M. Yu *et al.*, Lack of bcr and abr promotes hypoxia-induced pulmonary hypertension in mice. *PLoS One* **7**, e49756 (2012).

663. K. D. D. Gorrepati *et al.*, An SCF(FBXO28) E3 Ligase Protects Pancreatic beta-Cells from Apoptosis. *Int J Mol Sci* **19**, 975975 (2018).

664. Y. C. Kwon *et al.*, Promotion of Cancer Stem-Like Cell Properties in Hepatitis C Virus-Infected Hepatocytes. *J Virol* **89**, 11549-11556 (2015).

665. K. S. Burrack *et al.*, Myeloid Cell Arg1 Inhibits Control of Arthritogenic Alphavirus Infection by Suppressing Antiviral T Cells. *PLoS Pathog* **11**, e1005191 (2015).

666. S. B. Wortmann, J. A. Mayr, Choline-related-inherited metabolic diseases-A mini review. *J Inherit Metab Dis* **42**, 237-242 (2019).

667. K. Zhu, T. Zheng, X. Chen, H. Wang, Bioinformatic Analyses of Renal Ischaemia-Reperfusion Injury Models: Identification of Key Genes Involved in the Development of Kidney Disease. *Kidney Blood Press Res* **43**, 1898-1907 (2018).

668. M. Magnusson *et al.*, Dimethylglycine Deficiency and the Development of Diabetes. *Diabetes* **64**, 3010-3016 (2015).

669. M. Mennesson *et al.*, Kainate receptor auxiliary subunit NETO2 is required for normal fear expression and extinction. *Neuropsychopharmacology* **44**, 1855-1866 (2019).

670. Q. Ding *et al.*, ZEB2 Attenuates LPS-Induced Inflammation by the NF-kappaB Pathway in HK-2 Cells. *Inflammation* **41**, 722-731 (2018).

671. M. M. Minor *et al.*, Hepatitis B Virus HBx Protein Mediates the Degradation of Host Restriction Factors through the Cullin 4 DDB1 E3 Ubiquitin Ligase Complex. *Cells* **9**, (2020).

672. J. Fu *et al.*, The tumor suppressor gene WWOX links the canonical and noncanonical NF-kappaB pathways in HTLV-I Tax-mediated tumorigenesis. *Blood* **117**, 1652-1661 (2011).

673. S. Singla *et al.*, Loss of lung WWOX expression causes neutrophilic inflammation. *Am J Physiol Lung Cell Mol Physiol* **312**, L903-L911 (2017).

674. J. Trischler *et al.*, Immune Modulation of the T Cell Response in Asthma through Wnt10b. *Am J Respir Cell Mol Biol* **54**, 584-593 (2016).

675. J. Shen-Gunther, Q. Xia, W. Stacey, H. B. Asusta, Molecular Pap Smear: Validation of HPV Genotype and Host Methylation Profiles of ADCY8, CDH8, and ZNF582 as a Predictor of Cervical Cytopathology. *Front Microbiol* **11**, 595902 (2020).

676. H. Lin, X. Cao, Nuclear innate sensors for nucleic acids in immunity and inflammation. *Immunol Rev* **297**, 162-173 (2020).

677. L. Wang, M. Wen, X. Cao, Nuclear hnRNPA2B1 initiates and amplifies the innate immune response to DNA viruses. *Science* **365**, (2019).

678. T. Kessler *et al.*, Association of the coronary artery disease risk gene GUCY1A3 with ischaemic events after coronary intervention. *Cardiovasc Res* **115**, 1512-1518 (2019).

679. J. Xu *et al.*, Genome-Wide Profiling of Cervical RNA-Binding Proteins Identifies Human Papillomavirus Regulation of RNASEH2A Expression by Viral E7 and E2F1. *mBio* **10**, e02687-02618 (2019).

680. S. A. Newland *et al.*, The novel inhibitory receptor G6B is expressed on the surface of platelets and attenuates platelet function in vitro. *Blood* **109**, 4806-4809 (2007).

681. S. Curreli, Z. Arany, R. Gerardy-Schahn, D. Mann, N. M. Stamatos, Polysialylated neuropilin-2 is expressed on the surface of human dendritic cells and modulates dendritic cell-T lymphocyte interactions. *J Biol Chem* **282**, 30346-30356 (2007).

682. X. Liu *et al.*, The complex genetics of hypoplastic left heart syndrome. *Nat Genet* **49**, 1152-1159 (2017).

683. J. A. Lopez *et al.*, Bi-Allelic Mutations in STXBP2 Reveal a Complementary Role for STXBP1 in Cytotoxic Lymphocyte Killing. *Front Immunol* **9**, 529 (2018).

684. J. Choi *et al.*, Regulation of B cell receptor-dependent NF-kappaB signaling by the tumor suppressor KLHL14. *Proc Natl Acad Sci U S A* **117**, 6092-6102 (2020).

685. I. J. Chen *et al.*, Targeting the 15-keto-PGE2-PTGR2 axis modulates systemic inflammation and survival in experimental sepsis. *Free Radic Biol Med* **115**, 113-126 (2018).

686. L. Yan *et al.*, Peptidomic Analysis of Female Reproductive Tract Secretion to Identify Putative Anti-Infection Peptides in the Female Genital System via Nanotechnologies. *J Biomed Nanotechnol* **14**, 215-226 (2018).

687. G. Capalbo *et al.*, Inhibition of HIV-1 replication by small interfering RNAs directed against glioma pathogenesis related protein (GliPR) expression. *Retrovirology* **7**, 26 (2010).

688. G. S. Jung *et al.*, Pyruvate dehydrogenase kinase regulates hepatitis C virus replication. *Sci Rep* **6**, 30846 (2016).

689. B. Oehl-Jaschkowitz *et al.*, Deletions in 14q24.1q24.3 are associated with congenital heart defects, brachydactyly, and mild intellectual disability. *Am J Med Genet A* **164A**, 620-626 (2014).

690. Y. Wang *et al.*, Mitochondria-localised ZNFX1 functions as a dsRNA sensor to initiate antiviral responses through MAVS. *Nat Cell Biol* **21**, 1346-1356 (2019).

691. M. K. Brahma *et al.*, Increased Glucose Availability Attenuates Myocardial Ketone Body Utilization. *J Am Heart Assoc* **9**, e013039 (2020).

692. A. Gasparini, S. C. E. Tosatto, A. Murgia, E. Leonardi, Dynamic scaffolds for neuronal signaling: in silico analysis of the TANC protein family. *Sci Rep* **7**, 6829 (2017).

693. M. T. Heinonen, K. Kanduri, H. J. Lahdesmaki, R. Lahesmaa, T. A. Henttinen, Tubulin- and actin-associating GIMAP4 is required for IFN-gamma secretion during Th cell differentiation. *Immunol Cell Biol* **93**, 158-166 (2015).

694. M. T. Heinonen *et al.*, GIMAP GTPase family genes: potential modifiers in autoimmune diabetes, asthma, and allergy. *J Immunol* **194**, 5885-5894 (2015).

695. K. Takano *et al.*, An X-linked channelopathy with cardiomegaly due to a CLIC2 mutation enhancing ryanodine receptor channel activity. *Hum Mol Genet* **21**, 4497-4507 (2012).

696. S. Liu *et al.*, DNAH11 variants and its association with congenital heart disease and heterotaxy syndrome. *Sci Rep* **9**, 6683 (2019).

697. X. Zhao *et al.*, Zfyve16 regulates the proliferation of B-lymphoid cells. *Front Med* **12**, 559-565 (2018).

698. J. Wang *et al.*, RNA Binding Motif Protein RBM45 Regulates Expression of the 11-Kilodalton Protein of Parvovirus B19 through Binding to Novel Intron Splicing Enhancers. *mBio* **11**, e00192-00120 (2020).

699. F. Papaccio *et al.*, HGF/MET and the Immune System: Relevance for Cancer Immunotherapy. *Int J Mol Sci* **19**, 3595 (2018).

700. Y. Ito *et al.*, Influenza induces IL-8 and GM-CSF secretion by human alveolar epithelial cells through HGF/c-Met and TGF-alpha/EGFR signaling. *Am J Physiol Lung Cell Mol Physiol* **308**, L1178-1188 (2015).

701. D. Westermann *et al.*, Selective PDE5A inhibition with sildenafil rescues left ventricular dysfunction, inflammatory immune response and cardiac remodeling in angiotensin II-induced heart failure in vivo. *Basic Res Cardiol* **107**, 308 (2012).

702. A. Tanabe, J. Konno, K. Tanikawa, H. Sahara, Transcriptional machinery of TNF-alpha-inducible YTH domain containing 2 (YTHDC2) gene. *Gene* **535**, 24-32 (2014).

703. C. Y. Yu *et al.*, Dengue Virus Impairs Mitochondrial Fusion by Cleaving Mitofusins. *PLoS Pathog* **11**, e1005350 (2015).

704. D. Kwon, E. Park, S. J. Kang, Stimulator of IFN genes-mediated DNA-sensing pathway is suppressed by NLRP3 agonists and regulated by mitofusin 1 and TBC1D15, mitochondrial dynamics mediators. *FASEB J* **31**, 4866-4878 (2017).

705. J. L. J. Coleman *et al.*, Orphan receptor GPR37L1 contributes to the sexual dimorphism of central cardiovascular control. *Biol Sex Differ* **9**, 14 (2018).

706. V. Figliuolo da Paz, F. K. Ghishan, P. R. Kiela, Emerging Roles of Disabled Homolog 2 (DAB2) in Immune Regulation. *Front Immunol* **11**, 580302 (2020).

707. S. Bhattacharyya, T. J. Hope, J. A. Young, Differential requirements for clathrin endocytic pathway components in cellular entry by Ebola and Marburg glycoprotein pseudovirions. *Virology* **419**, 1-9 (2011).

708. R. Li *et al.*, Olfactomedin 1 Deficiency Leads to Defective Olfaction and Impaired Female Fertility. *Endocrinology* **156**, 3344-3357 (2015).

709. W. Zhang *et al.*, ERK/c-Jun Recruits Tet1 to Induce Zta Expression and Epstein-Barr Virus Reactivation through DNA Demethylation. *Sci Rep* **6**, 34543 (2016).

710. X. Li *et al.*, Methyltransferase Dnmt3a upregulates HDAC9 to deacetylate the kinase TBK1 for activation of antiviral innate immunity. *Nat Immunol* **17**, 806-815 (2016).

711. U. H. Beier, T. Akimova, Y. Liu, L. Wang, W. W. Hancock, Histone/protein deacetylases control Foxp3 expression and the heat shock response of T-regulatory cells. *Curr Opin Immunol* **23**, 670-678 (2011).

712. A. Broer, J. A. Cavanaugh, J. E. Rasko, S. Broer, The molecular basis of neutral aminoacidurias. *Pflugers Arch* **451**, 511-517 (2006).

713. D. L. Lin *et al.*, Dengue Virus Hijacks a Noncanonical Oxidoreductase Function of a Cellular Oligosaccharyltransferase Complex. *mBio* **8**, e00939-00917 (2017).

714. C. H. Yeh *et al.*, Mutation of epigenetic regulators TET2 and MLL3 in patients with HTLV-I-induced acute adult T-cell leukemia. *Mol Cancer* **15**, 15 (2016).

715. E. Iio *et al.*, TLL1 variant associated with development of hepatocellular carcinoma after eradication of hepatitis C virus by interferon-free therapy. *J Gastroenterol* **54**, 339-346 (2019).

716. K. A. Robertson *et al.*, An Interferon Regulated MicroRNA Provides Broad Cell-Intrinsic Antiviral Immunity through Multihit Host-Directed Targeting of the Sterol Pathway. *PLoS Biol* **14**, e1002364 (2016).

717. U. Singh, B. Westermark, CGGBP1--an indispensable protein with ubiquitous cytoprotective functions. *Ups. J. Med. Sci* **120**, 219-232 (2015).

718. D. Panda, D. J. Fernandez, M. Lal, E. Buehler, B. Moss, Triad of human cellular proteins, IRF2, FAM111A, and RFC3, restrict replication of orthopoxvirus SPI-1 host-range mutants. *Proc Natl Acad Sci U S A* **114**, 3720-3725 (2017).

719. M. Takano, T. Takeuchi, S. Kuriyama, R. Yumoto, Role of peptide transporter 2 and MAPK signaling pathways in the innate immune response induced by bacterial peptides in alveolar epithelial cells. *Life Sci* **229**, 173-179 (2019).

720. T. S. Fung, D. X. Liu, The ER stress sensor IRE1 and MAP kinase ERK modulate autophagy induction in cells infected with coronavirus infectious bronchitis virus. *Virology* **533**, 34-44 (2019).

721. R. Tiwari *et al.*, In silico and in vitro studies reveal complement system drives coagulation cascade in SARS-CoV-2 pathogenesis. *Comput Struct Biotechnol J* **18**, 3734-3744 (2020).

722. J. Dheekollu, P. M. Lieberman, The replisome pausing factor Timeless is required for episomal maintenance of latent Epstein-Barr virus. *J Virol* **85**, 5853-5863 (2011).

723. S. Hanzelmann *et al.*, Thrombin stimulates insulin secretion via protease-activated receptor-3. *Islets* **7**, e1118195 (2015).

724. A. Dharan *et al.*, Bicaudal D2 facilitates the cytoplasmic trafficking and nuclear import of HIV-1 genomes during infection. *Proc Natl Acad Sci U S A* **114**, E10707-E10716 (2017).

725. H. Fan *et al.*, DNA demethylation induces SALL4 gene re-expression in subgroups of hepatocellular carcinoma associated with Hepatitis B or C virus infection. *Oncogene* **36**, 2435-2445 (2017).

726. Y. Zhang, W. Knight, S. Chen, A. Mohan, C. Yan, Multiprotein Complex With TRPC (Transient Receptor Potential-Canonical) Channel, PDE1C (Phosphodiesterase 1C), and A2R (Adenosine A2 Receptor) Plays a Critical Role in Regulating Cardiomyocyte cAMP and Survival. *Circulation* **138**, 1988-2002 (2018).

727. B. Ramkhelawon *et al.*, Hypoxia induces netrin-1 and Unc5b in atherosclerotic plaques: mechanism for macrophage retention and survival. *Arterioscler Thromb Vasc Biol* **33**, 1180-1188 (2013).

728. M. Shimura *et al.*, Epigenetic displacement of HP1 from heterochromatin by HIV-1 Vpr causes premature sister chromatid separation. *J Cell Biol* **194**, 721-735 (2011).

729. M. Saha *et al.*, Consequences of MEGF10 deficiency on myoblast function and Notch1 interactions. *Hum Mol Genet* **26**, 2984-3000 (2017).

730. E. D. Hottz *et al.*, Platelet activation and platelet-monocyte aggregate formation trigger tissue factor expression in patients with severe COVID-19. *Blood* **136**, 1330-1341 (2020).

731. Z. Yu *et al.*, Lys29-linkage of ASK1 by Skp1-Cullin 1-Fbxo21 ubiquitin ligase complex is required for antiviral innate response. *Elife* **5**, e14087 (2016).

732. G. Ray, P. T. Schmitt, A. P. Schmitt, Angiomotin-Like 1 Links Paramyxovirus M Proteins to NEDD4 Family Ubiquitin Ligases. *Viruses* **11**, 128 (2019).

733. V. Vijayan *et al.*, A New Immunomodulatory Role for Peroxisomes in Macrophages Activated by the TLR4 Ligand Lipopolysaccharide. *J Immunol* **198**, 2414-2425 (2017).

734. S. Li *et al.*, Human endogenous retrovirus W family envelope gene activates the small conductance Ca2+-activated K+ channel in human neuroblastoma cells through CREB. *Neuroscience* **247**, 164-174 (2013).

735. Y. Cui, K. W. Liu, Y. Liang, M. S. Ip, J. C. Mak, Inhibition of monoamine oxidase-B by selegiline reduces cigarette smoke-induced oxidative stress and inflammation in airway epithelial cells. *Toxicol Lett* **268**, 44-50 (2017).

736. F. Anwar *et al.*, Targeting COVID-19 in Parkinson's patients: Drugs repurposed. *Curr Med Chem*, (2020).

737. M. M. Hu *et al.*, TRIM38 Negatively Regulates TLR3/4-Mediated Innate Immune and Inflammatory Responses by Two Sequential and Distinct Mechanisms. *J Immunol* **195**, 4415-4425 (2015).

738. M. M. Hu, H. B. Shu, Multifaceted roles of TRIM38 in innate immune and inflammatory responses. *Cell Mol Immunol* **14**, 331-338 (2017).

739. C. F. Moita, A. Chora, N. Hacohen, L. F. Moita, RNAi screen for kinases and phosphatases that play a role in antigen presentation by dendritic cells. *Eur J Immunol* **42**, 1843-1849 (2012).

740. N. Yamada *et al.*, Mutant KCNJ3 and KCNJ5 Potassium Channels as Novel Molecular Targets in Bradyarrhythmias and Atrial Fibrillation. *Circulation* **139**, 2157-2169 (2019).

741. J. O. Hendrickx *et al.*, GRK5 - A Functional Bridge Between Cardiovascular and Neurodegenerative Disorders. *Front Pharmacol* **9**, 1484 (2018).

742. M. L. H. Medel *et al.*, Prolactin Induces IL-2 Associated TRAIL Expression on Natural Killer Cells from Chronic Hepatitis C Patients In vivo and In vitro. *Endocr Metab Immune Disord Drug Targets* **19**, 975-984 (2019).

743. R. Ghaoui *et al.*, TOR1AIP1 as a cause of cardiac failure and recessive limb-girdle muscular dystrophy. *Neuromuscul Disord* **26**, 500-503 (2016).

744. Y. J. van de Vegte, B. S. Tegegne, N. Verweij, H. Snieder, P. van der Harst, Genetics and the heart rate response to exercise. *Cell Mol Life Sci* **76**, 2391-2409 (2019).

745. L. Dai *et al.*, IL-27 inhibits HIV-1 infection in human macrophages by down-regulating host factor SPTBN1 during monocyte to macrophage differentiation. *J Exp Med* **210**, 517-534 (2013).

746. G. Tarone, M. Brancaccio, The muscle-specific chaperone protein melusin is a potent cardioprotective agent. *Basic Res Cardiol* **110**, 10 (2015).

747. T. Ito *et al.*, Sprouty-Related Ena/Vasodilator-Stimulated Phosphoprotein Homology 1-Domain-Containing Protein-2 Critically Regulates Influenza A Virus-Induced Pneumonia. *Crit Care Med* **44**, e530-543 (2016).

748. S. M. Christensen *et al.*, Host and parasite responses in human diffuse cutaneous leishmaniasis caused by L. amazonensis. *PLoS Negl Trop Dis* **13**, e0007152 (2019).

749. L. Cao *et al.*, The Nuclear Matrix Protein SAFA Surveils Viral RNA and Facilitates Immunity by Activating Antiviral Enhancers and Super-enhancers. *Cell Host Microbe* **26**, 369-384 e368 (2019).

750. X. Jiang *et al.*, Interplay between HGAL and Grb2 proteins regulates B-cell receptor signaling. *Blood Adv* **3**, 2286-2297 (2019).

751. S. Lauttia *et al.*, Prokineticins and Merkel cell polyomavirus infection in Merkel cell carcinoma. *Br J Cancer* **110**, 1446-1455 (2014).

752. A. Grifoni *et al.*, Cutting Edge: Transcriptional Profiling Reveals Multifunctional and Cytotoxic Antiviral Responses of Zika Virus-Specific CD8(+) T Cells. *J Immunol* **201**, 3487-3491 (2018).

753. D. Burgner *et al.*, A genome-wide association study identifies novel and functionally related susceptibility Loci for Kawasaki disease. *PLoS Genet* **5**, e1000319 (2009).

754. R. Shaheen *et al.*, Positional mapping of PRKD1, NRP1 and PRDM1 as novel candidate disease genes in truncus arteriosus. *J Med Genet* **52**, 322-329 (2015).

755. H. C. Selinka, A. Wolde, M. Sauter, R. Kandolf, K. Klingel, Virus-receptor interactions of coxsackie B viruses and their putative influence on cardiotropism. *Med Microbiol Immunol* **193**, 127-131 (2004).

756. N. Scheller *et al.*, Translation and replication of hepatitis C virus genomic RNA depends on ancient cellular proteins that control mRNA fates. *Proc Natl Acad Sci U S A* **106**, 13517-13522 (2009).

757. Y. Cai *et al.*, Indispensable role of the Ubiquitin-fold modifier 1-specific E3 ligase in maintaining intestinal homeostasis and controlling gut inflammation. *Cell Discov* **5**, 7 (2019).

758. D. Li *et al.*, DDX56 inhibits type I interferon by disrupting assembly of IRF3-IPO5 to inhibit IRF3 nucleus import. *J Cell Sci* **133**, jcs230409 (2019).

759. Z. Xu, R. Anderson, T. C. Hobman, The capsid-binding nucleolar helicase DDX56 is important for infectivity of West Nile virus. *J Virol* **85**, 5571-5580 (2011).

760. K. C. Kim *et al.*, Identification of novel genes associated with HIV-1 latency by analysis of histone modifications. *Hum Genomics* **11**, 9 (2017).

761. M. Larsson *et al.*, Molecular signatures of T-cell inhibition in HIV-1 infection. *Retrovirology* **10**, 31 (2013).

762. C. Schultheiss *et al.*, Next-Generation Sequencing of T and B Cell Receptor Repertoires from COVID-19 Patients Showed Signatures Associated with Severity of Disease. *Immunity* **53**, 442-455 e444 (2020).

763. M. S. Gilardini Montani *et al.*, EBV reduces autophagy, intracellular ROS and mitochondria to impair monocyte survival and differentiation. *Autophagy* **15**, 652-667 (2019).

764. P. Gao *et al.*, ALG13 Deficiency Associated with Increased Seizure Susceptibility and Severity. *Neuroscience* **409**, 204-221 (2019).

765. R. R. Bradley, M. Terajima, Vaccinia virus K1L protein mediates host-range function in RK-13 cells via ankyrin repeat and may interact with a cellular GTPase-activating protein. *Virus Res* **114**, 104-112 (2005).

766. H. T. Kim *et al.*, Myh10 deficiency leads to defective extracellular matrix remodeling and pulmonary disease. *Nat Commun* **9**, 4600 (2018).

767. B. Taye *et al.*, Inter-Species Host Gene Expression Differences in Response to Human and Avian Influenza A Virus Strains. *Int J Mol Sci* **18**, 2295 (2017).

768. T. Zhang *et al.*, Transcript Profiling Identifies Early Response Genes against FMDV Infection in PK-15 Cells. *Viruses* **10**, 364 (2018).

769. M. Awazawa *et al.*, Deregulation of pancreas-specific oxidoreductin ERO1beta in the pathogenesis of diabetes mellitus. *Mol Cell Biol* **34**, 1290-1299 (2014).

770. M. Miranda *et al.*, Human subcutaneous adipose tissue LPIN1 expression in obesity, type 2 diabetes mellitus, and human immunodeficiency virus--associated lipodystrophy syndrome. *Metabolism* **56**, 1518-1526 (2007).

771. L. Mingorance *et al.*, Host phosphatidic acid phosphatase lipin1 is rate limiting for functional hepatitis C virus replicase complex formation. *PLoS Pathog* **14**, e1007284 (2018).

772. A. Joshi, H. Garg, K. Nagashima, J. S. Bonifacino, E. O. Freed, GGA and Arf proteins modulate retrovirus assembly and release. *Mol Cell* **30**, 227-238 (2008).

773. X. Wu *et al.*, pelo is required for high efficiency viral replication. *PLoS Pathog* **10**, e1004034 (2014).

774. T. Maruyama *et al.*, Loss of DDHD2, whose mutation causes spastic paraplegia, promotes reactive oxygen species generation and apoptosis. *Cell Death Dis* **9**, 797 (2018).

775. S. Fekrvand, R. Yazdani, H. Abolhassani, J. Ghaffari, A. Aghamohammadi, The First Purine Nucleoside Phosphorylase Deficiency Patient Resembling IgA Deficiency and a Review of the Literature. *Immunol Invest* **48**, 410-430 (2019).

776. Y. Zhang *et al.*, Binding of Avibirnavirus VP3 to the PIK3C3-PDPK1 complex inhibits autophagy by activating the AKT-MTOR pathway. *Autophagy* **16**, 1697-1710 (2020).

777. R. K. Mallampalli *et al.*, Targeting F box protein Fbxo3 to control cytokine-driven inflammation. *J Immunol* **191**, 5247-5255 (2013).

778. M. Kainulainen *et al.*, Virulence factor NSs of rift valley fever virus recruits the F-box protein FBXO3 to degrade subunit p62 of general transcription factor TFIIH. *J. Virol* **88**, 3464-3473 (2014).

779. S. S. Folmsbee, C. J. Gottardi, Cardiomyocytes of the Heart and Pulmonary Veins: Novel Contributors to Asthma? *Am J Respir Cell Mol Biol* **57**, 512-518 (2017).

780. F. Granberg, C. Svensson, U. Pettersson, H. Zhao, Adenovirus-induced alterations in host cell gene expression prior to the onset of viral gene expression. *Virology* **353**, 1-5 (2006).

781. C. D. Meshram *et al.*, Multiple Host Factors Interact with the Hypervariable Domain of Chikungunya Virus nsP3 and Determine Viral Replication in Cell-Specific Mode. *J Virol* **92**, e00838-00818 (2018).

782. O. M. Howard *et al.*, Histidyl-tRNA synthetase and asparaginyl-tRNA synthetase, autoantigens in myositis, activate chemokine receptors on T lymphocytes and immature dendritic cells. *J Exp Med* **196**, 781-791 (2002).

783. L. Lai *et al.*, MicroRNA-33 Regulates the Innate Immune Response via ATP Binding Cassette Transporter-mediated Remodeling of Membrane Microdomains. *J Biol Chem* **291**, 19651-19660 (2016).

784. H. Jiang *et al.*, Retinoic acid and liver X receptor agonist synergistically inhibit HIV infection in CD4+ T cells by up-regulating ABCA1-mediated cholesterol efflux. *Lipids Health Dis* **11**, 69 (2012).

785. N. Mukhamedova *et al.*, Analysis of ABCA1 and Cholesterol Efflux in HIV-Infected Cells. *Methods Mol Biol* **1354**, 281-292 (2016).

786. E. F. Griffin, X. Yan, K. A. Caldwell, G. A. Caldwell, Distinct functional roles of Vps41-mediated neuroprotection in Alzheimer's and Parkinson's disease models of neurodegeneration. *Hum Mol Genet* **27**, 4176-4193 (2018).

787. A. Dumas *et al.*, The HIV-1 protein Vpr impairs phagosome maturation by controlling microtubule-dependent trafficking. *J Cell Biol* **211**, 359-372 (2015).

788. A. G. Shabgah, E. Fattahi, F. Z. Shahneh, Interleukin-17 in human inflammatory diseases. *Postepy Dermatol Alergol* **31**, 256-261 (2014).

789. X. Z. Liu *et al.*, Hearing loss and PRPS1 mutations: Wide spectrum of phenotypes and potential therapy. *Int J Audiol* **52**, 23-28 (2013).

790. H. Xian, S. Yang, S. Jin, Y. Zhang, J. Cui, LRRC59 modulates type I interferon signaling by restraining the SQSTM1/p62-mediated autophagic degradation of pattern recognition receptor DDX58/RIG-I. *Autophagy* **16**, 408-418 (2020).

791. J. H. Kim, S. M. Park, J. H. Park, S. J. Keum, S. K. Jang, eIF2A mediates translation of hepatitis C viral mRNA under stress conditions. *EMBO J* **30**, 2454-2464 (2011).

792. B. Salek Esfahani *et al.*, Down-regulation of ERMN expression in relapsing remitting multiple sclerosis. *Metab Brain Dis* **34**, 1261-1266 (2019).

793. M. P. Buffon, D. A. Sortica, F. Gerchman, D. Crispim, L. H. Canani, FRMD3 gene: its role in diabetic kidney disease. A narrative review. *Diabetol Metab Syndr* **7**, 118 (2015).

794. S. H. Mozhgani, M. Zarei Ghobadi, M. Behnam Rad, M. Farzanehpour, F. Behzadian, Reconnaissance of the candidate genes involved in the pathogenesis of human immunodeficiency virus and targeted by antiretroviral therapy. *J Med Virol* **91**, 2134-2141 (2019).

795. P. Mehrbod *et al.*, The roles of apoptosis, autophagy and unfolded protein response in arbovirus, influenza virus, and HIV infections. *Virulence* **10**, 376-413 (2019).

796. T. S. Fung, M. Huang, D. X. Liu, Coronavirus-induced ER stress response and its involvement in regulation of coronavirus-host interactions. *Virus Res* **194**, 110-123 (2014).

797. X. Wang *et al.*, MiR-375 Has Contrasting Effects on Newcastle Disease Virus Growth Depending on the Target Gene. *Int J Biol Sci* **15**, 44-57 (2019).

798. N. R. Manchala, R. Dungdung, P. Trivedi, U. Unniyampurath, R. Pilankatta, Mycophenolic acid (MPA) modulates host cellular autophagy progression in sub genomic dengue virus-2 replicon cells. *Microb Pathog* **137**, 103762 (2019).

799. T. R. Reddy *et al.*, Specific interaction of HTLV tax protein and a human type IV neuronal intermediate filament protein. *Proc Natl Acad Sci U S A* **95**, 702-707 (1998).

800. Y. Ouyang *et al.*, Downregulated Gene Expression Spectrum and Immune Responses Changed During the Disease Progression in Patients With COVID-19. *Clin Infect Dis* **71**, 2052-2060 (2020).

801. J. H. Tan *et al.*, EMC6 regulates acinar apoptosis via APAF1 in acute and chronic pancreatitis. *Cell Death Dis* **11**, 966 (2020).

802. D. A. Costello, M. A. Lynch, Toll-like receptor 3 activation modulates hippocampal network excitability, via glial production of interferon-beta. *Hippocampus* **23**, 696-707 (2013).

803. A. M. Kamal *et al.*, Clinical importance of pharmacogenetics in the treatment of hepatitis C virus infection. *Rom J Morphol Embryol* **57**, 675-680 (2016).

804. M. Yu *et al.*, Signal inhibition by the dual-specific phosphatase 4 impairs T cell-dependent B-cell responses with age. *Proc Natl Acad Sci U S A* **109**, E879-888 (2012).

805. D. Deidda *et al.*, Antifungal, antibacterial, antiviral and cytotoxic activity of novel thio- and seleno-azoles. *Pharmacol Res* **36**, 193-197 (1997).

806. F. Allantaz-Frager *et al.*, Identification of biomarkers of response to IFNg during endotoxin tolerance: application to septic shock. *PLoS One* **8**, e68218 (2013).

807. A. W. Fischer *et al.*, PID1 regulates insulin-dependent glucose uptake by controlling intracellular sorting of GLUT4-storage vesicles. *Biochim Biophys Acta Mol Basis Dis* **1865**, 1592-1603 (2019).

808. R. Meazza *et al.*, Expression of HOXC4 homeoprotein in the nucleus of activated human lymphocytes. *Blood* **85**, 2084-2090 (1995).

809. D. Shimizu *et al.*, Infection of endotheliotropic human cytomegalovirus of trabecular meshwork cells. *Jpn J Ophthalmol* **62**, 667-676 (2018).

810. J. C. Cronk *et al.*, Methyl-CpG Binding Protein 2 Regulates Microglia and Macrophage Gene Expression in Response to Inflammatory Stimuli. *Immunity* **42**, 679-691 (2015).

811. J. C. Cronk *et al.*, Influenza A induces dysfunctional immunity and death in MeCP2-overexpressing mice. *JCI Insight* **2**, e88257 (2017).

812. J. Liu *et al.*, C5aR, TNF-alpha, and FGL2 contribute to coagulation and complement activation in virus-induced fulminant hepatitis. *J Hepatol* **62**, 354-362 (2015).

813. I. Shalev *et al.*, The Role of FGL2 in the Pathogenesis and Treatment of Hepatitis C Virus Infection. *Rambam Maimonides Med J* **1**, e0004 (2010).

814. H. Nishitsuji, M. Abe, R. Sawada, H. Takaku, ZBRK1 represses HIV-1 LTR-mediated transcription. *FEBS Lett* **586**, 3562-3568 (2012).

815. L. F. Monteiro, P. Y. M. Ferruzo, L. C. Russo, J. O. Farias, F. L. Forti, DUSP3/VHR: A Druggable Dual Phosphatase for Human Diseases. *Rev Physiol Biochem Pharmacol* **176**, 1-35 (2019).

816. K. Matsuura, M. Isogawa, Y. Tanaka, Host genetic variants influencing the clinical course of hepatitis B virus infection. *J Med Virol* **88**, 371-379 (2016).

817. I. F. Ueki *et al.*, Respiratory virus-induced EGFR activation suppresses IRF1-dependent interferon lambda and antiviral defense in airway epithelium. *J Exp Med* **210**, 1929-1936 (2013).

818. M. Takahama *et al.*, The RAB2B-GARIL5 Complex Promotes Cytosolic DNA-Induced Innate Immune Responses. *Cell Rep* **20**, 2944-2954 (2017).

819. P. Xia *et al.*, Glutamylation of the DNA sensor cGAS regulates its binding and synthase activity in antiviral immunity. *Nat Immunol* **17**, 369-378 (2016).

820. J. O'Prey, S. Wilkinson, K. M. Ryan, Tumor antigen LRRC15 impedes adenoviral infection: implications for virus-based cancer therapy. *J Virol* **82**, 5933-5939 (2008).

821. Y. Kubo *et al.*, Rab3a-Bound CD63 Is Degraded and Rab3a-Free CD63 Is Incorporated into HIV-1 Particles. *Front Microbiol* **8**, 1653 (2017).

822. Y. Yang, Y. H. Hu, Y. Liu, Wdfy1 deficiency impairs Tlr3-mediated immune responses in vivo. *Cell Mol Immunol* **17**, 1014-1016 (2020).

823. C. I. Real *et al.*, Identification of proteins that mediate the pro-viral functions of the interferon stimulated gene 15 in hepatitis C virus replication. *Antiviral Res* **100**, 654-661 (2013).

824. D. L. Mallery *et al.*, Cellular IP6 Levels Limit HIV Production while Viruses that Cannot Efficiently Package IP6 Are Attenuated for Infection and Replication. *Cell Rep* **29**, 3983-3996 e3984 (2019).

825. E. Wustenhagen *et al.*, The Cytoskeletal Adaptor Obscurin-Like 1 Interacts with the Human Papillomavirus 16 (HPV16) Capsid Protein L2 and Is Required for HPV16 Endocytosis. *J Virol* **90**, 10629-10641 (2016).
