# Supplementary material for: Leveraging publicly available coronavirus data to identify new therapeutic targets for COVID-19
Source: PLoS One. 2021 Sep 29;16(9):e0257965. doi: 10.1371/journal.pone.0257965 (PMC8480897; doi:10.1371/journal.pone.0257965)
Supplement: Attachment — Submitted filename: Response to Reviewers Covid MS PlosOne 6July2021.pdf [file pone.0257965.s002.pdf]

utmb Health

DEPARTMENT OF ANESTHESIOLOGY

Prasenjit Mitra, MD
Academic Editor
PLOS ONE

6 July, 2021

MS: PONE-D-21-09155
Leveraging publicly available coronavirus data to identify new therapeutic targets for COVID-19

Dear Dr. Mitra,

On behalf of my coauthors, we thank you and the reviewer very much for the positive review of our manuscript. We have addressed the reviewer's critiques to the best of our ability and hope that these changes address most of these concerns.

We would like to explain some extenuating circumstances concerning this study. Given our experience with bioinformatic analysis of miRNA datasets in the Gene Expression Omnibus and our recent study in PlosOne showing that this type of analysis can identify biomarkers of many human diseases (Sell et al., 2020, https://pubmed.ncbi.nlm.nih.gov/32502186/), in February, 2020, given the urgency in understanding the pathogenesis of the global Covid-19 pandemic, we thought we could perform a similar *in silico* analysis of existing coronavirus data in GEO and find novel insights into the deadly mechanisms of SARS-CoV-2 infection. We initiated this analysis during the Covid-19 lockdown at our institution, the University of Texas Medical Branch and because the lockdown with severe restrictions was extended over 15 months due to the huge numbers of Covid-19 patients at the hospital, all interactions among the co-authors were virtual and all literature cited were obtained via web access. As a consequence of the lockdown and related issues with departmental funding, two of the coauthors are no longer working full time at UTMB and one of the coauthors is working in another state; thus, due to the lack of funding (no external funding was available for this study) and lack of other resources, no further analysis of these datasets is possible and neither are extensive revisions of the manuscript. The senior author had the sole responsibility for completing the revisions and the response to reviewers. Thus, in the responses to each of the reviewer's queries, some of the issues raised could not be fully addressed. Nonetheless, we believe the substance of the analysis, although much is inferred from the literature and could be considered speculative (as we indicate in the manuscript), is based on a thorough examination of the available literature on the coronavirus-induced gene targets and provides a basis for future studies of coronavirus-host defense interactions.

We have addressed all of the journal requirements as stated in your email. We have added a couple new citations to the manuscript and the original list of citations in the manuscript and supplemental references have been checked for accuracy. Because this was an *in silico* analysis of existing datasets in GEO, we need to point out that all the data that we downloaded are already publicly available and in the public domain and was acknowledged as such in our original manuscript.

Reviewer #1: *The authors have undertaken bioinformatic analysis of four coronavirus datasets to assess the differential expression of various anti-viral and pro-viral genes. The results have been discussed appropriately taking into consideration the numerous gene obtained from the analysis.*

The following points may be addressed:
1. A short clinico-pathological comparative description of MERS to COVID & SARS CoV 1 to SARS CoV 2 indicating the probable relevance of the genes discussed would be appropriate.

Response: Respectfully, we would like to point out that the original intent of this *in silico* analysis was to identify biologically relevant host genes affected by the viral pathogens from previously deposited coronavirus datasets that may prove to be novel therapeutic targets and infer from published reports their functional relevance to host defenses. Our literature analysis showed that many of these pro-viral or antiviral host response genes were also targeted by many diverse viral pathogens known to infect human hosts. We did not intend to describe a comprehensive comparison of MERS, SARS-CoV-1 and SARS-COV-2 since hundreds of such comparisons have been published in the past year and a half since the beginning of the Covid-19 pandemic; our focus was on identifying novel gene targets of these viruses. To our knowledge, our *in silico* analysis of the gene targets of coronavirus-induced host genes is among the first to show that these host response genes are also implicated in the pathogenesis of many other diverse viruses; the implication is that broad spectrum antiviral drugs that are efficacious for other viral pathogens may be effective for Covid-19 patients but this remains to be proven in future studies. We also believe that the functional relevance of the genes have been addressed throughout the manuscript and further discussion would veer upon more speculation than necessary.

2. Are there any original studies which have explored the temporal suppression of antiviral immune response in COVID when compared with other viral infections ? This may substantiate the temporal suppression of antiviral immune response observed in the analysis.

Response: During the time we did the *in silico* analysis of these coronavirus datasets and wrote the manuscript, there were some unpublished reports in preprint servers suggesting that SARS-CoV-2 did indeed suppress the antiviral immune response as our analysis indicated. Most did not address the temporal suppression as described in our manuscript. Only recently were such reports published in peer-reviewed journals. We now cite, in the manuscript, one such supporting study by Finkel et al., that was recently published in Nature (https://www.nature.com/articles/s41586-021-03610-3).

3. Line 254: How miR-139-3p expressed at low levels in both SARS-CoV-1 and SARS-CoV-2 cells as well as untreated control cells can be attributed to provide permissive environment ?

Response: In other words, since miRNA expression is inversely related to their gene targets, i.e. if high, their target genes are suppressed and if low, like miR-139-3p, their gene targets are highly expressed or upregulated, the naturally occurring expression of some host genes that are the targets of these miRNAs may help pathogenic viruses infect host cells. Since that was a

speculative comment on our part, we clarified this statement in the manuscript. What we meant was “ *viruses could exploit and coopt the endogenously expressed host proteins to facilitate entry into host cells and favor the replication of viral proteins*”.

4. Line 364: It is unclear how the present analysis identify genes involved in COVID-19 induced chronic symptoms, rather it will give insight into COVID associated complication.

Response: The chronic symptoms described in the manuscript have been extensively documented in many published studies of hundreds of Covid-19 patients this past year and some of those studies are cited in our manuscript. We believe, based on the literature analysis of the function of the coronavirus-induced host gene targets, that not only does our study provide insight into Covid associated complications but also insight into the chronic symptoms reported by so called “Covid long haul” patients. For example, loss of smell is a frequently reported chronic health problem in Covid-19 survivors and the persistent symptoms of myocarditis, diabetes and so-called brain fog in Covid-19 survivors are among the chronic symptoms that can be linked to the dysregulated expression of some of the coronavirus gene targets mentioned in our study. We would like to point out that this study was not meant to be a comprehensive treatise of Covid-19-associated gene changes in human hosts; our intent was to identify novel gene targets that could be therapeutically targeted with existing or new coronavirus drugs in development. The four coronavirus datasets analyzed in this study represent the three recent and current causes of severe coronavirus outbreaks in human populations and were chosen without any other preconceived bias; we only present examples from the literature supporting the likely functional roles of the viral gene targets and we will leave it up to the scientific readership to infer the association of these genes with reported Covid symptoms based on the analysis presented in our study.

Thank you very much for the opportunity to submit our revised manuscript.

Regards,

Helen Lee Hellmich, PhD
Associate Professor
Department of Anesthesiology
University of Texas Medical Branch
